# Text Dialogue Analysis for Primary Screening of Mild Cognitive Impairment: Development and Validation Study

**DOI:** 10.2196/51501

**Published:** 2023-12-29

**Authors:** Changyu Wang, Siru Liu, Aiqing Li, Jialin Liu

**Affiliations:** 1 Department of Medical Informatics West China Medical School Sichuan University Chengdu China; 2 West China College of Stomatology Sichuan University Chengdu China; 3 Department of Biomedical Informatics Vanderbilt University Medical Center Nashville, TN United States; 4 Department of Neurology West China Hospital Sichuan University Chengdu China; 5 Information Center West China Hospital Sichuan University Chengdu China; 6 Department of Otolaryngology-Head and Neck Surgery West China Hospital Sichuan University Chengdu China

**Keywords:** artificial intelligence, AI, AI models, ChatGPT, primary screening, mild cognitive impairment, standardization, prompt design, design, artificial intelligence, cognitive impairment, screening, model, clinician, diagnosis

## Abstract

**Background:**

Artificial intelligence models tailored to diagnose cognitive impairment have shown excellent results. However, it is unclear whether large linguistic models can rival specialized models by text alone.

**Objective:**

In this study, we explored the performance of ChatGPT for primary screening of mild cognitive impairment (MCI) and standardized the design steps and components of the prompts.

**Methods:**

We gathered a total of 174 participants from the DementiaBank screening and classified 70% of them into the training set and 30% of them into the test set. Only text dialogues were kept. Sentences were cleaned using a macro code, followed by a manual check. The prompt consisted of 5 main parts, including character setting, scoring system setting, indicator setting, output setting, and explanatory information setting. Three dimensions of variables from published studies were included: vocabulary (ie, word frequency and word ratio, phrase frequency and phrase ratio, and lexical complexity), syntax and grammar (ie, syntactic complexity and grammatical components), and semantics (ie, semantic density and semantic coherence). We used R 4.3.0. for the analysis of variables and diagnostic indicators.

**Results:**

Three additional indicators related to the severity of MCI were incorporated into the final prompt for the model. These indicators were effective in discriminating between MCI and cognitively normal participants: tip-of-the-tongue phenomenon (*P*<.001), difficulty with complex ideas (*P*<.001), and memory issues (*P*<.001). The final GPT-4 model achieved a sensitivity of 0.8636, a specificity of 0.9487, and an area under the curve of 0.9062 on the training set; on the test set, the sensitivity, specificity, and area under the curve reached 0.7727, 0.8333, and 0.8030, respectively.

**Conclusions:**

ChatGPT was effective in the primary screening of participants with possible MCI. Improved standardization of prompts by clinicians would also improve the performance of the model. It is important to note that ChatGPT is not a substitute for a clinician making a diagnosis.

## Introduction

Alzheimer disease (AD) is the most common form of dementia, causing abnormal mental decline, involving thinking, memory, and language, and severely affecting the patient’s quality of life [1]. AD has always been a major health hazard to society and a huge burden to the health care system, and this burden will even increase in the future [2,3]. There is an urgent need for human society to carry out prevention and intervention for AD. Although AD is progressive and incurable, early detection, diagnosis, and treatment can effectively delay its progression and thus significantly improve the quality of life of patients [4]. Mild cognitive impairment (MCI) has been recognized as a strong predictor of AD and represents an early stage of AD; early diagnosis and intervention of MCI will be of great help to human society in the fight against AD [4-6]. However, neuropsychological testing for MCI is time-consuming and rigorous. To accommodate the potentially large population with MCI in society, a variety of validated brief cognitive tests, including language tests, memory tests, the Mini-Mental State Examination (MMSE), and the Montreal Cognitive Assessment (MoCA), are widely used for early screening of the disease [7-9]. Developments in artificial intelligence (AI) are likely to make this process more accessible, enabling a broader group of people to receive its benefit [10].

AI has emerged as a promising tool in health care, particularly in cognitive impairment diagnosis [11,12]. AI can provide a more accurate and standardized process for diagnosing and predicting diseases [13]. Thabtah et al [14] conducted a comprehensive analysis of mobile-based AD and MCI screening apps, highlighting the potential of AI applications for the early detection and diagnosis of dementia. The study also explored the use of AI to improve access to health care services. Kalafatis et al [15] demonstrated the convergent validity of the Integrated Cognitive Assessment (ICA) using cognitive tests, such as the MoCA [15]. The AI model of the ICA was able to detect cognitive impairment with an area under the curve (AUC) of 0.81 for patients with MCI and an AUC of 0.88 for patients with mild AD [15]. In contrast to custom-built AI models, we would like to see if large language models can be applied to this domain with good outcomes [16,17].

ChatGPT is a high-level large language model developed by OpenAI, designed to generate human-like text based on the input it receives [18]. GPT-4, the latest version available as of the September 2021 knowledge deadline, has a larger model size in terms of parameters and improved text generation capabilities than its predecessors [19,20]. The generative pretrained transformer (GPT) model is a neural network architecture for understanding data sequences such as text. ChatGPT is pretrained on a large corpus of text data, enabling it to understand and generate text that is not only grammatically correct but also contextually relevant and coherent over a long period of time [21]. With its powerful text analysis capabilities, we were ready to explore whether ChatGPT could be used for primary screening of MCI based on text conversation analysis under the supervision of neurologists [22]. We will discuss the process and components of a standardized prompt design [23].

It is crucial to emphasize that ChatGPT is only intended as a primary screening tool and does not have a diagnostic role [24]. This study aims to explore the feasibility of using ChatGPT for the primary screening of MCI through the analysis of text-based conversations. Furthermore, it seeks to establish a standardized protocol for prompt design in this specific application, following the guidance of expert neurologists.

## Methods

### Inclusion and Exclusion of Participants

**Figure 1:**
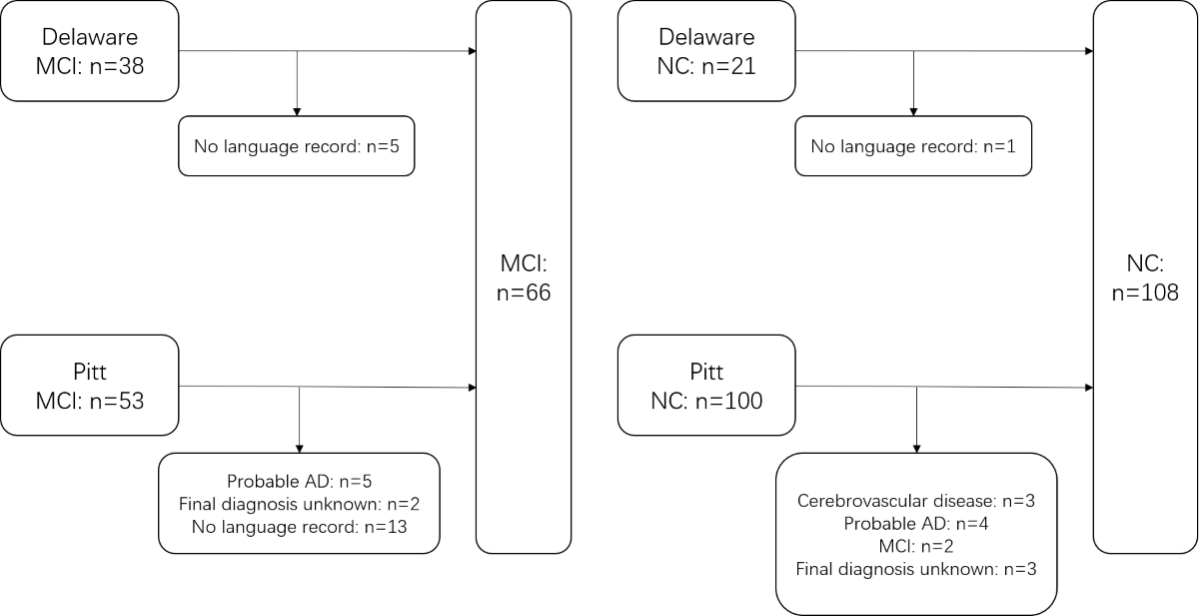
Participants inclusion and exclusion. AD: Alzheimer disease; MCI: mild cognitive impairment; NC: normal cognition.

We included a total of 174 participants from the DementiaBank English Protocol Delaware Corpus [25] and the DementiaBank English Pitt Corpus [6,26] (Figure 1). The initial data set included participants from the Delaware Corpus and the Pitt Corpus who were assessed using the Everyday Cognition Questionnaire, the Clinical Dementia Rating Scale, and the MMSE score to ensure that participants were accurately classified into the MCI category. Selection criteria for both corpora excluded other systemic diseases or brain disorders that could lead to cognitive decline (Multimedia Appendix 1 includes descriptions and selection criteria for both corpora).

A total of 66 participants with MCI were included. In the Delaware database, we excluded 5 patients with no language record data and finally included 33 participants. In the Pitt database, we finally included 33 participants; 30 of them were from the dementia group and 3 from the control group. The specific screening method was as follows: we screened 36 participants from the dementia group by initial diagnosis of MCI; we then excluded 5 participants with probable AD, 2 participants with unknown final diagnosis, and 13 participants with no language record data. We also included 3 participants with a final diagnosis of MCI from the control group.

A total of 108 cognitively normal participants were included. In the Delaware database, we excluded 1 patient with no language record data and finally included 20 participants with 20 language records obtained. In the Pitt database, there were a total of 100 participants in the control group with language record data, and we finally included 88 participants. The specific screening method was as follows: we excluded controls with cerebrovascular disease since participants were ultimately diagnosed with probable AD or MCI. Controls without a definitive diagnosis, controls with a final diagnosis of probable AD, controls with a final diagnosis of MCI, and controls with a final diagnosis of cerebrovascular disease were also excluded.

In the end, 66 participants with MCI and 108 participants with normal cognitive function were included.

### Ethical Considerations

All data used in this study were obtained from publicly available databases (DementiaBank data set), archived by TalkBank. TalkBank abides by its Code of Ethics, which complements but does not replace the commonly accepted professional codes, such as the American Psychological Association Code of Ethics and the American Anthropological Association Code of Ethics [27]. It should be emphasized that the data do not contain individual patient information, which obviates the need for ethical approval and individual patient consent. In addition, all protected health information was appropriately anonymized to ensure compliance with data protection regulations. Therefore, this study was exempt from ethics approval by the Bioethics Committee of the West China Hospital of Sichuan University.

### Data Cleaning and Distribution

First, only the text dialogues “PARTICIPANT” and “INVESTIGATOR” were kept. Then each sentence was cleaned using the macro codes (Multimedia Appendix 1). Finally, a manual check was performed by CW.

Random numbers were generated using the RAND and RANK functions to renumber all participants. We used these random numbers to split the data set randomly into 70% training data and 30% test data. The training set contained 44 participants with MCI and 78 participants with normal cognitive function, and the test set contained 22 participants with MCI and 30 participants with normal cognitive function (Multimedia Appendix 2).

### Model Prompt Design

#### Model 1 Prompt

We identified the basic indicators for analyzing language features based on published studies, including vocabulary features, syntax and grammar features, as well as semantics features in 3 main areas, with a total of 7 indicators (Table 1) [28-31].

**Table 1:** Analysis of indicators and evaluation of indicators.

Classification	Analysis of indicators	Evaluation of indicators
Vocabulary features	Word frequency and word ratio, phrase frequency and phrase ratio, and lexical complexity	Limited vocabulary, increased verbosity, overlearned phrases, improper use of pronouns, hesitation (word-finding difficulties), circumlocution (word-finding difficulties), tip-of-the-tongue phenomenon, and repetition
Syntax and grammar features	Syntactic complexity and grammatical components	simplified sentence structure and grammar, grammatical errors, and difficulty with complex ideas
Semantics features	Semantic density and semantic coherence	Semantic paraphasias, lack of coherence, consistency in errors, lack of semantic fluency, disorganized narrative structure, orientation issues, decline in conversational engagement and responsiveness, memory issues, lack of abstract thinking and synthesis, and abnormal emotions

#### Model 2 Prompt

At the end of the Model 1 training, participants received Feedback 1, which included more detailed MCI features. This information was used to design the new prompt.

#### Model 3 Prompt

We set a new prompt based on Feedback 2 generated by Model 2. Differently, we extracted 21 severity assessment indicators related to MCI and designed a scoring system (Table 1).

#### Model 4 Prompt

We allowed GPT-4 to design 3 prompts for itself based on the Model 3 prompt and integrated them. This helped to compare the differences between the manual and robotic designs and standardize the format of the subsequent prompts.

#### Model 5 Prompt

**Figure 2:**
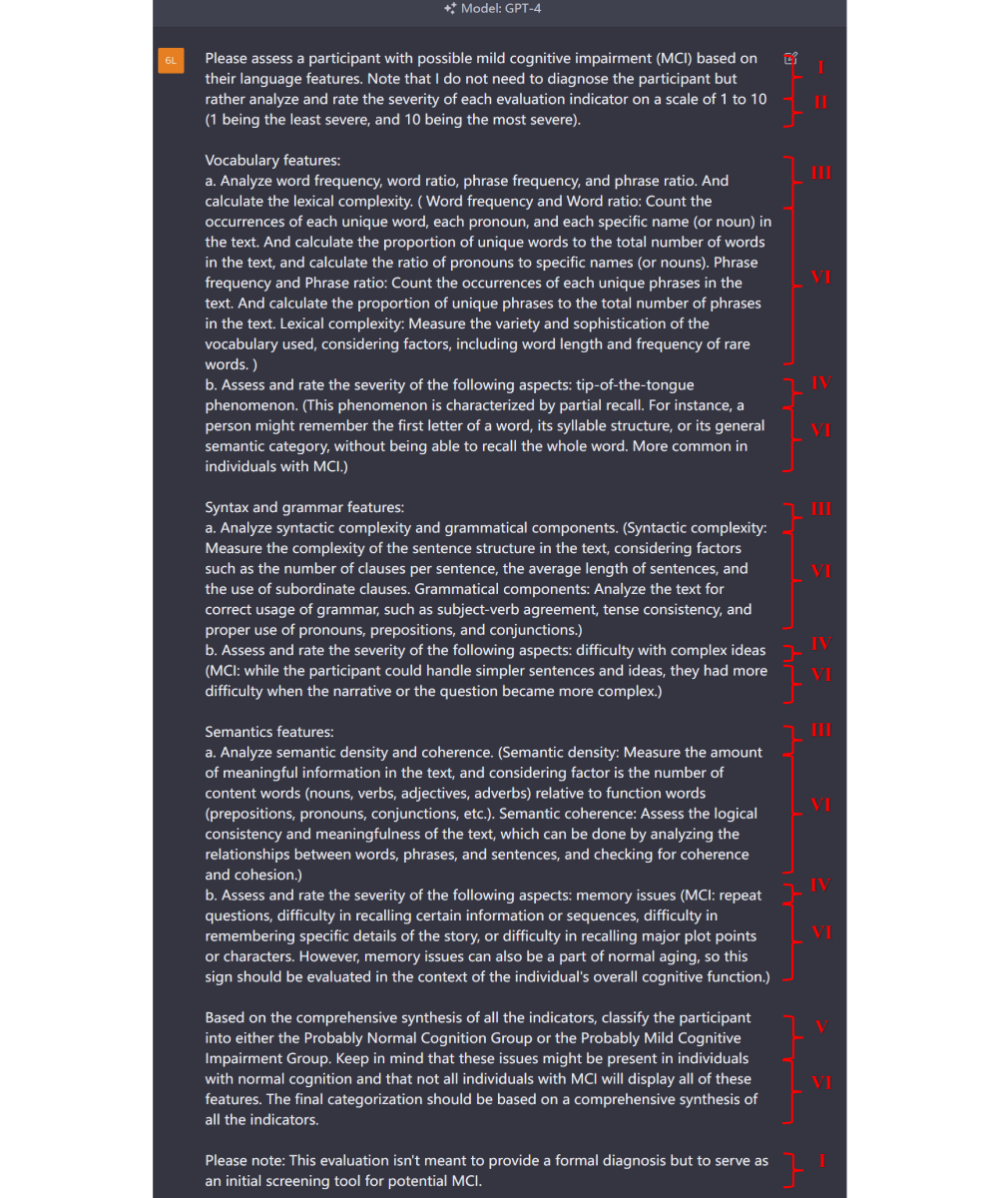
Prompt Design. The prompt consists of 5 main parts. The character setting is shown as Ⅰ. The scoring system setting is shown as Ⅱ. The indicator settings are shown as Ⅲ and Ⅳ. Indicators include those included based on literature (ie, shown as Ⅲ) and those included based on analysis (ie, shown as Ⅳ). The output setting is shown as Ⅴ. The explanatory information settings are shown as Ⅵ.

The 21 indicators obtained from the GPT-4 Model 3 to assess the severity of MCI were analyzed using statistical methods to select more statistically significant indicators and to reduce bias. The prompt format of Model 4 was then followed for the design. The prompt was designed in 4 steps. In the first step, high-quality literature (preferably high-quality systematic reviews) was found, and proven valid indicators were extracted and added to the prompts with an interpretation of these indicators. In the second step, ChatGPT was required to output the results by analyzing these indicators, while interpreting the association between each patient’s symptoms and the indicators. The new explanatory information was collected, organized, reviewed, and finally added to the prompt. In the third step, the indicators were scored, and these scores were statistically analyzed to select those that better discriminated between the experimental and control groups. Finally, by standardizing the format of the prompt, the indicators selected in step 3 were incorporated, and the information collected in step 2 was used to interpret them. The prompt consisted of 5 main parts (Figure 2). The first part was character setting (ie, designing a role for ChatGPT). In this research, ChatGPT was set up as a physician’s assistant who, by analyzing indicators, would first classify the participants into the probable MCI group and the probable normal cognitive function group; it would then provide detailed reasons for doing so, to facilitate the diagnosis by physicians. The second part was the establishment of a scoring system. Its purpose was to quantify the narrative indicators to facilitate subsequent statistical analysis. The third part was the definition of indicators. Indicators were divided into those included based on the literature and those included based on the analysis. The quality, rather than the quantity of the included indicators, was ensured. The fourth part was the output setting. This part required matching with the first part. The fifth part was the explanatory information setting. Providing valid explanatory information, derived from the literature or analysis for each indicator, could improve the performance of the models.

For clarity, our experiments were conducted from May 11, 2023, to June 1, 2023. During this period, we specifically used GPT-4 and GPT-3.5. This ensures that all responses analyzed were consistent in terms of the version’s capabilities and potential biases. All model prompts, feedback, and detailed explanations of each indicator are available in the Multimedia Appendix 1.

### Statistics Analysis

The outcome of our study is the diagnosis of MCI, and the independent variables are the responses generated by GPT-3.5 and GPT-4 to the prompts designed across different models.

Descriptive statistics, including the calculation of mean, SD, median, IQR, as well as minimum and maximum values were performed for each variable in each group. We then analyzed the normal distribution and the homogeneity of variance test of the data for all variables. On the one hand, if the variables were normally distributed and had a uniform variance, a one-way ANOVA was used for the 4 groups—true positive (TP), false positive (FP), false negative (FN), and true negative (TN)—and an independent samples *t* test (2-tailed) was used for the 2 groups—positive and negative. If there were differences in the results of the one-way ANOVA, a two-way comparison was performed using the Tukey Honestly Significant Difference test as a post hoc test. On the other hand, if the variables were not normally distributed, the Kruskal-Wallis *H* test was used for the 4 groups, and the Mann-Whitney *U* test was used for the 2 groups. If there were differences in the results of the Kruskal-Wallis *H* test, the Dunn test was applied as a post hoc test (Multimedia Appendix 2). Finally, the visualization of violin plots was used to compare the degree of concentration and dispersion of each variable.

Sensitivity, specificity, positive predictive value, negative predictive value, positive likelihood ratios, negative likelihood ratios, accuracy, receiver operating characteristics curve, and the AUC of the different models were compared (Multimedia Appendix 2). Analysis was performed using R 4.3.0 (R Foundation for Statistical Computing). All R codes can be found in Multimedia Appendix 1.

## Results

### Inclusion of Variables

**Figure 3:**
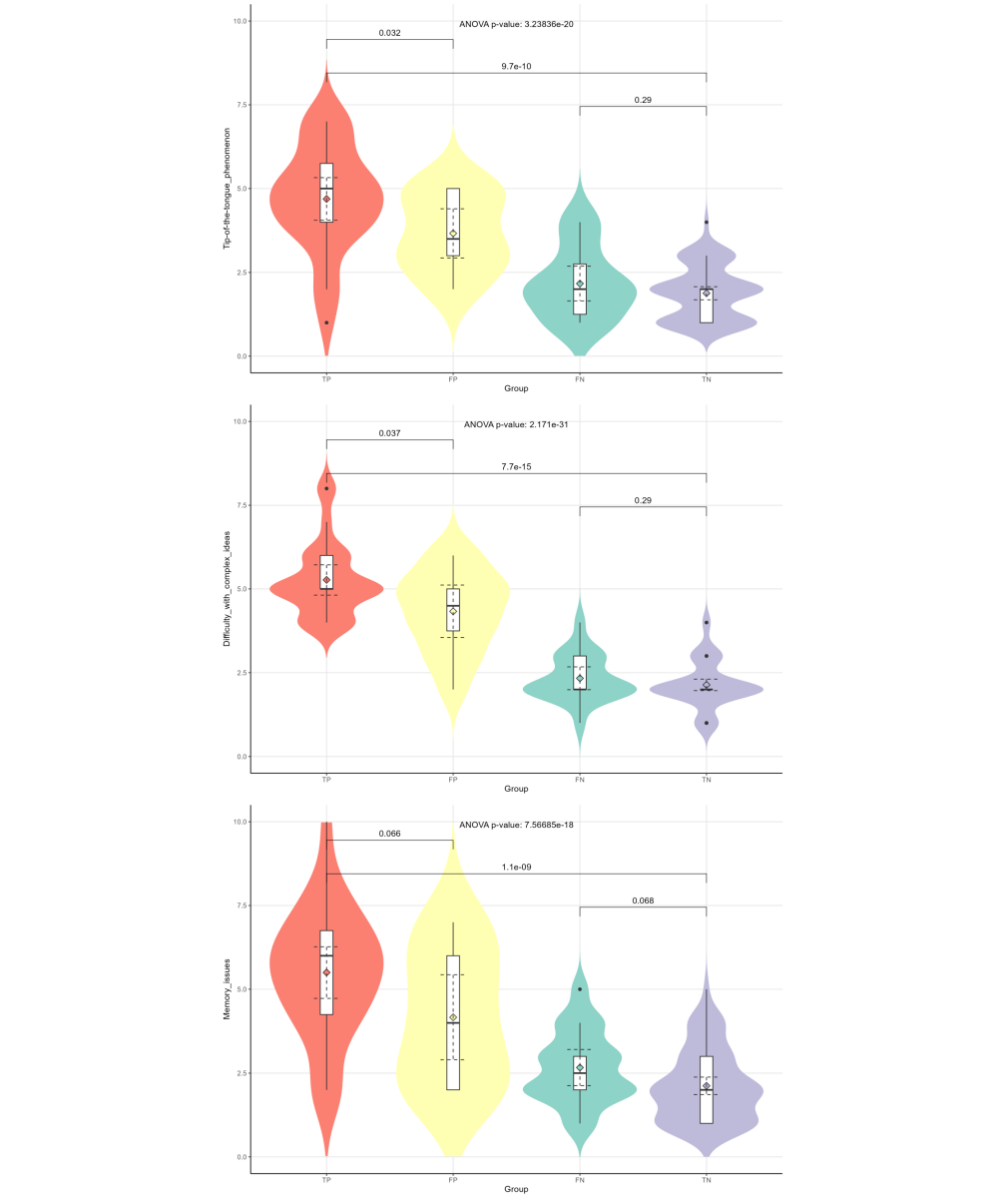
The violin plots of the selected evaluation metrics. Tip-of-the-tongue phenomenon, difficulty with complex ideas, and memory problems, 3 metrics for evaluating participants' cognitive functioning, were included in the prompt of Model 5. The violin plots include *P* values from one-way ANOVA and *P* values from the Tukey Honestly Significant Difference test without adjustment for multiple comparisons.

In addition to the 8 analytic indicators that had been explicitly included in the Model 5 prompts, we included 3 evaluative indicators in the final prompt. These 3 indicators were generated from 21 indicators (Table 1) that assessed the severity of MCI, and they were effective in discriminating between MCI and cognitively normal participants, namely tip-of-the-tongue phenomenon (*P*<.001), difficulty with complex ideas (*P*<.001), and memory problems (*P*<.001; Figure 3). Although most of the indicators were effective in distinguishing FP-FN, TP-FN, TN-FP, and TP-TN, only the tip-of-the-tongue phenomenon (without adjustment for multiple comparisons: *P*=.03; with adjustment for multiple comparisons: *P*=.04), difficulty with complex ideas (without adjustment for multiple comparisons: *P*=.04; with adjustment for multiple comparisons: *P*=.01), and memory problem (without adjustment for multiple comparisons: *P*=.07; with adjustment for multiple comparisons: *P*=.03) could effectively discriminate TP-FP (Figure 3). The statistical analysis of all indicators can be found in Multimedia Appendix 1.

### Model Comparison

**Figure 4:**
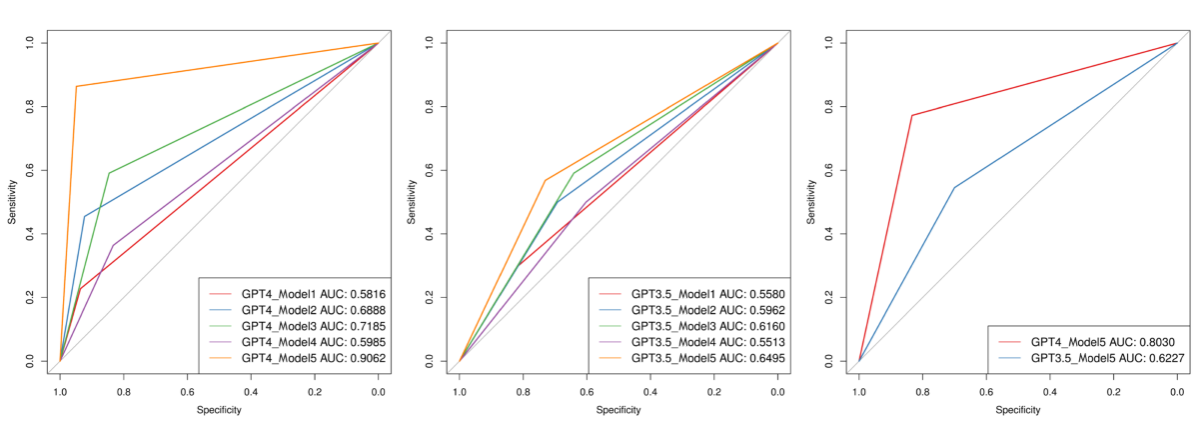
Receiver operating characteristic curve. In the training set, the final Model 5 achieves an area under the curve (AUC) of 0.9062 in GPT-4 and 0.6459 in GPT-3.5. Model 5 outperforms Models 1-4 in both GPT-4 and GPT-3.5. In the test set, Model 5 achieves an AUC of 0.8030 in GPT-4 and 0.6227 in GPT-3.5.

We found that ChatGPT had acceptable performance in screening for MCI by text analysis alone (Table 2 and Figure 4). On the training set, the sensitivity, specificity, and AUC of the GPT-4 Model 5 reached 0.8636, 0.9487, and 0.9062, respectively; on the test set, the sensitivity, specificity, and AUC reached 0.7727, 0.8333, and 0.8030, respectively. The prompt of Model 5 consisted of 5 main parts, including character setting, scoring system setting, indicator setting, output setting, and explanatory information setting.

**Table 2:** Diagnostic evaluation indicators.

Dataset	model	TP^a^	FN^b^	FP^c^	TN^d^	Sensitivity	Specificity	PPV^e^	NPV^f^	PLR^g^	NLR^h^	Accuracy
Training set	Gpt-4 Model 1	10	34	5	73	0.2273	0.9359	0.6667	0.6822	3.5455	0.8257	0.6803
Training set	GPT-4 Model 2	20	24	6	72	0.4545	0.9231	0.7692	0.7500	5.9091	0.5909	0.7541
Training set	GPT-4 Model 3	26	18	12	66	0.5909	0.8462	0.6842	0.7857	3.8409	0.4835	0.7541
Training set	GPT-4 Model 4	16	28	13	65	0.3636	0.8333	0.5517	0.6989	2.1818	0.7636	0.6639
Training set	GPT-4 Model 5	38	6	4	74	0.8636	0.9487	0.9048	0.9250	16.8409	0.1437	0.9180
Training set	GPT-3.5 Model 1	13	31	14	64	0.2955	0.8205	0.4815	0.6737	1.6461	0.8587	0.6311
Training set	GPT-3.5 Model 2	22	22	24	54	0.5000	0.6923	0.4783	0.7105	1.6250	0.7222	0.6230
Training set	GPT-3.5 Model 3	26	18	28	50	0.5909	0.6410	0.4815	0.7353	1.6461	0.6382	0.6230
Training set	GPT-3.5 Model 4	22	22	31	47	0.5000	0.6026	0.4151	0.6812	1.2581	0.8298	0.5656
Training set	GPT-3.5 Model 5	25	19	21	57	0.5682	0.7308	0.5435	0.7500	2.1104	0.5909	0.6721
Test set	GPT-4 Model 5	17	5	5	25	0.7727	0.8333	0.7727	0.8333	4.6364	0.2727	0.8077
Test set	GPT-3.5 Model 5	12	10	9	21	0.5455	0.7000	0.5714	0.6774	1.8182	0.6494	0.6346

^a^TP: true positive.

^b^FN: false negative.

^c^FP: false positive.

^d^TN: true negative.

^e^PPV: positive predictive value.

^f^NPV: negative predictive value.

^g^PLR: positive likelihood ratio.

^h^NLR: negative likelihood ratio.

By comparing Model 1, Model 2, and Model 3 (GPT-4 AUC for the models: 0.5816, 0.6888, and 0.7185, respectively; GPT-3.5 AUC for the models: 0.5580, 0.5962, and 0.6160, respectively), we found that providing ChatGPT with more detailed prompts would effectively improve its ability to screen for MCI.

In addition, comparing model 3 and model 4 (GPT-4 AUC: 0.7185 and 0.5985, respectively; GPT-3.5 AUC: 0.6160 and 0.5513, respectively) showed that the physician-designed prompt was superior to the ChatGPT designed prompt.

The analysis and screening of the included indicators would effectively improve the performance of the model, as shown by the comparison of model 3 and model 5 (GPT-4 AUC: 0.7185 and 0.9062, respectively; GPT-3.5 AUC: 0.6160 and 0.6495, respectively).

We also verified that GPT-4 outperformed GPT-3.5 in terms of logical ability, which was reflected in the following two points: first, the AUC of GPT-4 was consistently higher than that of GPT-3.5 under the same model in the same data set; second, the improvement in the prompt was more significant for the performance improvement of GPT-4 compared to GPT-3.5.

## Discussion

### Main Findings

In our study, we used a text-dialogue analysis approach to evaluate the suitability of ChatGPT for primary screening for MCI and developed a standardized methodology for the design of prompts in this application. Comparative analyses across various ChatGPT models revealed that the more detailed prompts crafted by medical professionals (Model 5) significantly outperformed the less detailed prompts generated by ChatGPT (Models 1-4). This underscored the valuable role of human expertise in enhancing model performance. Furthermore, GPT-4 consistently outperformed GPT-3.5 across all models, demonstrating its superior logical capabilities and responsiveness to cue enhancements.

In exploring the art of prompt creation, we proposed a comprehensive and multifaceted approach. First, we conducted a thorough literature search, focusing on systematic reviews, to systematically identify and understand reliable indicators. Next, ChatGPT played a crucial role in uncovering the relationships between these indicators and patient symptoms, helping us to incorporate this knowledge into the prompt. We then used statistical analyses to score and discriminate the key indicators that differentiate between the experimental and control groups. The outcome of this process was the development of a coherent prompt blueprint that prioritized consistency and clarity. To ensure the effectiveness of the prompt, we recommend incorporating 5 essential pillars, as follows:

1. Character definition: this pillar involves framing ChatGPT as a physician’s assistant, establishing a professional persona that aligns with the intended purpose.
2. Robust scoring mechanism for narrative indicators: developing a reliable system for quantitatively assessing narrative indicators.
3. Integration of literature-informed and analysis-driven indicators: combining indicators gleaned from the literature with those derived from statistical analysis, while emphasizing their quality and relevance.
4. Alignment with established character: ensuring that the output generated by ChatGPT resonates with the established character, maintaining consistency in the model’s responses.
5. Explanatory information: providing coherent explanatory information alongside each indicator to potentially enhance the model's diagnosis performance and comprehensibility.

### Comparison to Prior Work

The diagnostic process for MCI encompasses a multifaceted evaluation, integrating clinical observations, cognitive assessments, and feedback from both individuals and their family members regarding their daily functioning [32]. Language analysis can be a valuable tool, but it is just one aspect of the overall assessment [33]. Generally, individuals with MCI may exhibit subtle differences in their language use compared to those with normal cognitive abilities. Some potential indications of MCI in language use include 8 lexical features: limited vocabulary, increased verbosity, overlearned phrases, incorrect use of pronouns, hesitation (reflecting word-finding difficulties), circumlocution (indicative of word-finding difficulties), the tip-of-the-tongue phenomenon, and repetition [28,30,31,34]. Additionally, 3 syntactic and grammatical features include simplified sentence structures and grammar, grammatical errors, and challenges in handling complex ideas. Furthermore, 10 semantic features encompass semantic paraphasias, lack of coherence, consistent errors, diminished semantic fluency, disjointed narrative structure, orientation issues, declining engagement and responsiveness during conversation, memory-related issues, reduced capacity for abstract thinking and synthesis, and emotional aberrations [35-37]. Our research highlights the significance of paying attention to the tip-of-the-tongue phenomenon, difficulties with complex ideas, and memory-related issues when assessing individuals with MCI.

### Strengths of the Study

This study developed a standardized methodology for designing prompts for MCI screening using ChatGPT. The comparative analysis provided valuable insights into the capabilities of different ChatGPT models and versions, contributing to the understanding of their potential applications within the medical domain. Our research results highlighted the superior performance of prompts created by physicians compared to those generated by ChatGPT. Specifically, Model 4 outperformed both Model 3 and Model 5. This finding strongly suggests that, despite ChatGPT’s advanced abilities, human expertise in health care is essential for making effective decisions. This is especially evident in ChatGPT’s difficulty in determining which indicators are most important in the decision-making process.

The combination of prompts generated by human experts and the computational capabilities of ChatGPT shows great potential in clinical applications. This collaborative approach can be a valuable tool for physicians, offering initial insights and analyses based on relevant indicators. As a result, it can streamline the diagnostic process and potentially enhance overall clinical decision-making.

### Study Limitations

First, our investigation has uncovered a lack of valid indicators that can accurately distinguish between TNs and FNs. This finding raises concerns about the potential for ChatGPT to make mistakes in categorizing participants into the potentially cognitively normal group. Second, our study focused solely on comparing GPT-4 with GPT-3.5. Although the prompts were carefully reviewed by clinical experts and the model exhibited favorable statistical performance, additional validation is necessary to determine whether ChatGPT can be reliably used as a primary screening tool for MCI. In future research endeavors, we intend to expand upon this comparative analysis by evaluating the effectiveness of GPT-4 for clinicians with varying levels of expertise, aiming to elucidate its clinical utility in primary screening. Third, our test data set used never-trained data, all of which had to be requested and some of which were only published in 2022, but there was still no guarantee that ChatGPT had not learned them, which is a concern in the era of large models. To mitigate this concern, we augmented our data set with patient information from our institution to ensure complete novelty for ChatGPT. Finally, our study did not comprehensively address the consistency and variability of ChatGPT responses. Using ChatGPT to generate only 1 response may introduce bias. In the context of our study, the primary aim was to establish a conceptual framework for ChatGPT prompts. Consequently, a single prompt per model was considered sufficient to demonstrate the potential and trajectory of our immediate design evolution. In the subsequent study of GPT-4 comparisons with different levels of clinicians, we reassessed our results by sending each prompt multiple times to the GPT-4 Model 5 and used statistical methods to measure the distribution and concentration trends of ChatGPT responses to repeated prompts.

### Conclusions

In both the training and test sets, ChatGPT could effectively discriminate participants with possible MCI. Meanwhile, standardization of prompts by physicians would improve the performance of the model. It should be noted, however, that the use of ChatGPT must follow medical ethics and cannot replace doctors in diagnosis. Through the study, we hope to screen people who may have MCI and help them get to the hospital for diagnosis, so that early detection, early diagnosis, and early treatment can be achieved, delaying or even preventing the progression of MCI to AD.

## Appendix

Multimedia Appendix 1 Supplementary information.

Multimedia Appendix 2 Participant information.

## Abbreviations

AD: Alzheimer disease
AI: artificial intelligence
AUC: area under the curve
FN: false negative
FP: false positive
GPT: generative pretrained transformer
ICA: Integrated Cognitive Assessment
MCI: mild cognitive impairment
MMSE: Mini-Mental State Examination
MoCA: Montreal Cognitive Assessment
TN: true negative
TP: true positive

## Data Availability

The DementiaBank data set [38] used in this research is safeguarded by a password, and access is restricted to members of the DementiaBank Consortium Group. Permission to access this data set can be obtained by becoming a member of the DementiaBank Consortium Group. Informed consent was provided by all subjects according to the DementiaBank.

## References

[1] Alzheimer’s disease and related dementias. Centers for Disease Control and Prevention.

[2] Matthews KA, Xu W, Gaglioti AH, Holt JB, Croft JB, Mack D, McGuire LC (2019). Racial and ethnic estimates of Alzheimer's disease and related dementias in the United States (2015-2060) in adults aged ≥65 years. Alzheimers Dement.

[3] Mokdad AH, Ballestros K, Echko M, Glenn S, Olsen HE, Mullany E, Lee A, Khan AR, Ahmadi A, Ferrari AJ, Kasaeian A, Werdecker A, Carter A, Zipkin B, Sartorius B, Serdar B, Sykes BL, Troeger C, Fitzmaurice C, Rehm CD, Santomauro D, Kim D, Colombara D, Schwebel DC, Tsoi D, Kolte D, Nsoesie E, Nichols E, Oren E, Charlson FJ, Patton GC, Roth GA, Hosgood HD, Whiteford HA, Kyu H, Erskine HE, Huang H, Martopullo I, Singh JA, Nachega JB, Sanabria JR, Abbas K, Ong K, Tabb K, Krohn KJ, Cornaby L, Degenhardt L, Moses M, Farvid M, Griswold M, Criqui M, Bell M, Nguyen M, Wallin M, Mirarefin M, Qorbani M, Younis M, Fullman N, Liu P, Briant P, Gona P, Havmoller R, Leung R, Kimokoti R, Bazargan-Hejazi S, Hay SI, Yadgir S, Biryukov S, Vollset SE, Alam T, Frank T, Farid T, Miller T, Vos T, Bärnighausen Till, Gebrehiwot TT, Yano Y, Al-Aly Z, Mehari A, Handal A, Kandel A, Anderson B, Biroscak B, Mozaffarian D, Dorsey ER, Ding EL, Park E, Wagner G, Hu G, Chen H, Sunshine JE, Khubchandani J, Leasher J, Leung J, Salomon J, Unutzer J, Cahill L, Cooper L, Horino M, Brauer M, Breitborde N, Hotez P, Topor-Madry R, Soneji S, Stranges S, James S, Amrock S, Jayaraman S, Patel T, Akinyemiju T, Skirbekk V, Kinfu Y, Bhutta Z, Jonas JB, Murray CJL, US Burden of Disease Collaborators (2018). The state of US health, 1990-2016: burden of diseases, injuries, and risk factors among US states. JAMA.

[4] What is Alzheimer’s disease?. Alzheimer’s Association.

[5] Snowdon DA, Kemper SJ, Mortimer JA, Greiner LH, Wekstein DR, Markesbery WR (1996). Linguistic ability in early life and cognitive function and Alzheimer's disease in late life. Findings from the Nun Study. JAMA.

[6] Yang Q, Li X, Ding X, Xu F, Ling Z (2022). Deep learning-based speech analysis for Alzheimer's disease detection: a literature review. Alzheimers Res Ther.

[7] Hemmy LS, Linskens EJ, Silverman PC, Miller MA, Talley KMC, Taylor BC, Ouellette JM, Greer NL, Wilt TJ, Butler M, Fink HA (2020). Brief cognitive tests for distinguishing clinical Alzheimer-type dementia from mild cognitive impairment or normal cognition in older adults with suspected cognitive impairment. Ann Intern Med.

[8] Zhuang L, Yang Y, Gao J (2021). Cognitive assessment tools for mild cognitive impairment screening. J Neurol.

[9] Mattke S, Batie D, Chodosh J, Felten K, Flaherty E, Fowler NR, Kobylarz FA, O'Brien K, Paulsen R, Pohnert A, Possin KL, Sadak T, Ty D, Walsh A, Zissimopoulos JM (2023). Expanding the use of brief cognitive assessments to detect suspected early-stage cognitive impairment in primary care. Alzheimers Dement.

[10] Graham SA, Lee EE, Jeste DV, Van Patten R, Twamley EW, Nebeker C, Yamada Y, Kim H, Depp CA (2020). Artificial intelligence approaches to predicting and detecting cognitive decline in older adults: a conceptual review. Psychiatry Res.

[11] Sirilertmekasakul C, Rattanawong W, Gongvatana A, Srikiatkhachorn A (2023). The current state of artificial intelligence-augmented digitized neurocognitive screening test. Front Hum Neurosci.

[12] Lee J, Lim JM (2022). Factors associated with the experience of cognitive training apps for the prevention of dementia: cross-sectional study using an extended health belief model. J Med Internet Res.

[13] Merkin A, Krishnamurthi R, Medvedev ON (2022). Machine learning, artificial intelligence and the prediction of dementia. Curr Opin Psychiatry.

[14] Thabtah F, Peebles D, Retzler J, Hathurusingha C (2020). Dementia medical screening using mobile applications: a systematic review with a new mapping model. J Biomed Inform.

[15] Kalafatis C, Modarres MH, Apostolou P, Marefat H, Khanbagi M, Karimi H, Vahabi Z, Aarsland D, Khaligh-Razavi S (2021). Validity and cultural generalisability of a 5-minute AI-based, computerised cognitive assessment in mild cognitive impairment and Alzheimer's dementia. Front Psychiatry.

[16] Jiang LY, Liu XC, Nejatian NP, Nasir-Moin M, Wang D, Abidin A, Eaton K, Riina HA, Laufer I, Punjabi P, Miceli M, Kim NC, Orillac C, Schnurman Z, Livia C, Weiss H, Kurland D, Neifert S, Dastagirzada Y, Kondziolka D, Cheung ATM, Yang G, Cao M, Flores M, Costa AB, Aphinyanaphongs Y, Cho K, Oermann EK (2023). Health system-scale language models are all-purpose prediction engines. Nature.

[17] Zhong Y, Chen Y, Zhou Y, Lyu Y, Yin J, Gao Y (2023). The Artificial intelligence large language models and neuropsychiatry practice and research ethic. Asian J Psychiatr.

[18] Introducing ChatGPT. OpenAI.

[19] GPT-4 is OpenAI’s most advanced system, producing safer and more useful responses. OpenAI.

[20] OpenAI (2023). GPT-4 Technical Report. ArXiv.

[21] Language models can explain neurons in language models.

[22] Li R, Kumar A, Chen JH (2023). How chatbots and large language model artificial intelligence systems will reshape modern medicine: fountain of creativity or Pandora's box?. JAMA Intern Med.

[23] Giray L (2023). Prompt engineering with ChatGPT: a guide for academic writers. Ann Biomed Eng.

[24] Cadamuro J, Cabitza F, Debeljak Z, De Bruyne S, Frans G, Perez SM, Ozdemir H, Tolios A, Carobene A, Padoan A (2023). Potentials and pitfalls of ChatGPT and natural-language artificial intelligence models for the understanding of laboratory medicine test results. An assessment by the European Federation of Clinical Chemistry and Laboratory Medicine (EFLM) Working Group on Artificial Intelligence (WG-AI). Clin Chem Lab Med.

[25] Lanzi AM, Saylor AK, Fromm D, Liu H, MacWhinney B, Cohen ML (2023). DementiaBank: theoretical rationale, protocol, and illustrative analyses. Am J Speech Lang Pathol.

[26] Becker JT, Boller F, Lopez OL, Saxton J, McGonigle KL (1994). The natural history of Alzheimer's disease. Description of study cohort and accuracy of diagnosis. Arch Neurol.

[27] TalkBank.

[28] Vigo I, Coelho L, Reis S (2022). Speech- and language-based classification of Alzheimer's disease: a systematic review. Bioengineering (Basel).

[29] Colla D, Delsanto M, Agosto M, Vitiello B, Radicioni DP (2022). Semantic coherence markers: the contribution of perplexity metrics. Artif Intell Med.

[30] Balagopalan A, Eyre B, Robin J, Rudzicz F, Novikova J (2021). Comparing pre-trained and feature-based models for prediction of Alzheimer's disease based on speech. Front Aging Neurosci.

[31] Roark B, Mitchell M, Hosom J, Hollingshead K, Kaye J (2011). Spoken language derived measures for detecting mild cognitive impairment. IEEE Trans Audio Speech Lang Process.

[32] Weinstein AM, Gujral S, Butters MA, Bowie CR, Fischer CE, Flint AJ, Herrmann N, Kennedy JL, Mah L, Ovaysikia S, Pollock BG, Rajji TK, Mulsant BH (2022). Diagnostic precision in the detection of mild cognitive impairment: a comparison of two approaches. Am J Geriatr Psychiatry.

[33] Fraser KC, Meltzer JA, Rudzicz F (2016). Linguistic features identify Alzheimer's disease in narrative speech. J Alzheimers Dis.

[34] Haulcy R, Glass J (2020). Classifying Alzheimer's disease using audio and text-based representations of speech. Front Psychol.

[35] Taler V, Voronchikhina A, Gorfine G, Lukasik M (2016). Knowledge of semantic features in mild cognitive impairment. J Neurolinguistics.

[36] Joubert S, Gardy L, Didic M, Rouleau I, Barbeau EJ (2021). A meta-analysis of semantic memory in mild cognitive impairment. Neuropsychol Rev.

[37] Taler V, Monetta L, Sheppard C, Ohman A (2019). Semantic function in mild cognitive impairment. Front Psychol.

[38] DementiaBank. TalkBank.

