# Supplementary material for: Text Dialogue Analysis for Primary Screening of Mild Cognitive Impairment: Development and Validation Study
Source: J Med Internet Res. 2023 Dec 29;25:e51501. doi: 10.2196/51501 (PMC10787336; doi:10.2196/51501)
Supplement: Multimedia Appendix 1 [file jmir_v25i1e51501_app1.docx]

**Supplementary Information**

Catalog

**Macro code 2**

**Model prompts, Example inputs, and Feedbacks** **11**

**Model 1 prompt** 11

**Example input 1** 13

**Feedback 1** 16

**Model 2 prompt** 17

**Example input 2** 19

**Feedback 2** 22

**Model 3 prompt** 25

**Example input 3** 28

**Designed by GPT-4** 32

**Model 4 prompt** 38

**Example input 4** 39

**Model 5 prompt** 42

**Example input 5** 44

**R codes 47**

**R code 1** 47

**R code 2** 51

**R code 3** 53

**Statistical analysis 56**

**Statistical analysis 1: mean, standard deviation, median, interquartile spacing, minimum, maximum values** 56

**Statistical analysis 2: normal distribution, Homogeneity of variance test, Independent samples t-test for the two groups, Mann-Whitney U test for the two groups** 59

**Statistical analysis 3 and Violin plots: one-way ANOVA for the four groups (Tukey's Honestly Significant Difference test as a post hoc test), and Kruskal-Wallis H-test for the four groups (Dunn's test was applied as a post hoc test)** 60

**Introduction to corpus and selection criteria** 74

**Macro code**: Use the macro code to clean up each dialogue of "PARTICIPANT" and "INVESTIGATOR".

Sub macro 1()

'

' macro 1 macro

'

'

Selection.Find.ClearFormatting

Selection.Find.Replacement.ClearFormatting

With Selection.Find

.Text = "^#"

.Replacement.Text = ""

.Forward = True

.Wrap = wdFindContinue

.Format = False

.MatchCase = False

.MatchWholeWord = False

.MatchByte = True

.MatchWildcards = False

.MatchSoundsLike = False

.MatchAllWordForms = False

End With

Selection.Find.Execute Replace:=wdReplaceAll

With Selection.Find

.Text = Chr(21) & "_" & Chr(21)

.Replacement.Text = ""

.Forward = True

.Wrap = wdFindContinue

.Format = False

.MatchCase = False

.MatchWholeWord = False

.MatchByte = True

.MatchWildcards = False

.MatchSoundsLike = False

.MatchAllWordForms = False

End With

Selection.Find.Execute Replace:=wdReplaceAll

With Selection.Find

.Text = " _ "

.Replacement.Text = ""

.Forward = True

.Wrap = wdFindContinue

.Format = False

.MatchCase = False

.MatchWholeWord = False

.MatchByte = True

.MatchWildcards = False

.MatchSoundsLike = False

.MatchAllWordForms = False

End With

Selection.Find.Execute Replace:=wdReplaceAll

With Selection.Find

.Text = ChrW(8225)

.Replacement.Text = ""

.Forward = True

.Wrap = wdFindContinue

.Format = False

.MatchCase = False

.MatchWholeWord = False

.MatchByte = True

.MatchWildcards = False

.MatchSoundsLike = False

.MatchAllWordForms = False

End With

Selection.Find.Execute Replace:=wdReplaceAll

With Selection.Find

.Text = "+//"

.Replacement.Text = ""

.Forward = True

.Wrap = wdFindContinue

.Format = False

.MatchCase = False

.MatchWholeWord = False

.MatchByte = True

.MatchWildcards = False

.MatchSoundsLike = False

.MatchAllWordForms = False

End With

Selection.Find.Execute Replace:=wdReplaceAll

With Selection.Find

.Text = "+/"

.Replacement.Text = ""

.Forward = True

.Wrap = wdFindContinue

.Format = False

.MatchCase = False

.MatchWholeWord = False

.MatchByte = True

.MatchWildcards = False

.MatchSoundsLike = False

.MatchAllWordForms = False

End With

Selection.Find.Execute Replace:=wdReplaceAll

With Selection.Find

.Text = "+<"

.Replacement.Text = ""

.Forward = True

.Wrap = wdFindContinue

.Format = False

.MatchCase = False

.MatchWholeWord = False

.MatchByte = True

.MatchWildcards = False

.MatchSoundsLike = False

.MatchAllWordForms = False

End With

Selection.Find.Execute Replace:=wdReplaceAll

With Selection.Find

.Text = "+""/"

.Replacement.Text = ""

.Forward = True

.Wrap = wdFindContinue

.Format = False

.MatchCase = False

.MatchWholeWord = False

.MatchByte = True

.MatchWildcards = False

.MatchSoundsLike = False

.MatchAllWordForms = False

End With

Selection.Find.Execute Replace:=wdReplaceAll

With Selection.Find

.Text = "&=sighs"

.Replacement.Text = ""

.Forward = True

.Wrap = wdFindContinue

.Format = False

.MatchCase = False

.MatchWholeWord = False

.MatchByte = True

.MatchWildcards = False

.MatchSoundsLike = False

.MatchAllWordForms = False

End With

Selection.Find.Execute Replace:=wdReplaceAll

With Selection.Find

.Text = "&=coughs"

.Replacement.Text = ""

.Forward = True

.Wrap = wdFindContinue

.Format = False

.MatchCase = False

.MatchWholeWord = False

.MatchByte = True

.MatchWildcards = False

.MatchSoundsLike = False

.MatchAllWordForms = False

End With

Selection.Find.Execute Replace:=wdReplaceAll

With Selection.Find

.Text = "&=laughs"

.Replacement.Text = ""

.Forward = True

.Wrap = wdFindContinue

.Format = False

.MatchCase = False

.MatchWholeWord = False

.MatchByte = True

.MatchWildcards = False

.MatchSoundsLike = False

.MatchAllWordForms = False

End With

Selection.Find.Execute Replace:=wdReplaceAll

With Selection.Find

.Text = "[+ exc]"

.Replacement.Text = ""

.Forward = True

.Wrap = wdFindContinue

.Format = False

.MatchCase = False

.MatchWholeWord = False

.MatchByte = True

.MatchWildcards = False

.MatchSoundsLike = False

.MatchAllWordForms = False

End With

Selection.Find.Execute Replace:=wdReplaceAll

With Selection.Find

.Text = "[+ gram]"

.Replacement.Text = ""

.Forward = True

.Wrap = wdFindContinue

.Format = False

.MatchCase = False

.MatchWholeWord = False

.MatchByte = True

.MatchWildcards = False

.MatchSoundsLike = False

.MatchAllWordForms = False

End With

Selection.Find.Execute Replace:=wdReplaceAll

With Selection.Find

.Text = "*INV"

.Replacement.Text = "INVESTIGATOR"

.Forward = True

.Wrap = wdFindContinue

.Format = False

.MatchCase = False

.MatchWholeWord = False

.MatchByte = True

.MatchWildcards = False

.MatchSoundsLike = False

.MatchAllWordForms = False

End With

Selection.Find.Execute Replace:=wdReplaceAll

With Selection.Find

.Text = "*PAR"

.Replacement.Text = "PARTICIPANT"

.Forward = True

.Wrap = wdFindContinue

.Format = False

.MatchCase = False

.MatchWholeWord = False

.MatchByte = True

.MatchWildcards = False

.MatchSoundsLike = False

.MatchAllWordForms = False

End With

Selection.Find.Execute Replace:=wdReplaceAll

With Selection.Find

.Text = "[//]"

.Replacement.Text = "…"

.Forward = True

.Wrap = wdFindContinue

.Format = False

.MatchCase = False

.MatchWholeWord = False

.MatchByte = True

.MatchWildcards = False

.MatchSoundsLike = False

.MatchAllWordForms = False

End With

Selection.Find.Execute Replace:=wdReplaceAll

With Selection.Find

.Text = "[/]"

.Replacement.Text = "…"

.Forward = True

.Wrap = wdFindContinue

.Format = False

.MatchCase = False

.MatchWholeWord = False

.MatchByte = True

.MatchWildcards = False

.MatchSoundsLike = False

.MatchAllWordForms = False

End With

Selection.Find.Execute Replace:=wdReplaceAll

With Selection.Find

.Text = "&+"

.Replacement.Text = "…"

.Forward = True

.Wrap = wdFindContinue

.Format = False

.MatchCase = False

.MatchWholeWord = False

.MatchByte = True

.MatchWildcards = False

.MatchSoundsLike = False

.MatchAllWordForms = False

End With

Selection.Find.Execute Replace:=wdReplaceAll

With Selection.Find

.Text = "&-"

.Replacement.Text = "…"

.Forward = True

.Wrap = wdFindContinue

.Format = False

.MatchCase = False

.MatchWholeWord = False

.MatchByte = True

.MatchWildcards = False

.MatchSoundsLike = False

.MatchAllWordForms = False

End With

Selection.Find.Execute Replace:=wdReplaceAll

With Selection.Find

.Text = "&~"

.Replacement.Text = "…"

.Forward = True

.Wrap = wdFindContinue

.Format = False

.MatchCase = False

.MatchWholeWord = False

.MatchByte = True

.MatchWildcards = False

.MatchSoundsLike = False

.MatchAllWordForms = False

End With

Selection.Find.Execute Replace:=wdReplaceAll

With Selection.Find

.Text = "+"

.Replacement.Text = ""

.Forward = True

.Wrap = wdFindContinue

.Format = False

.MatchCase = False

.MatchWholeWord = False

.MatchByte = True

.MatchWildcards = False

.MatchSoundsLike = False

.MatchAllWordForms = False

End With

Selection.Find.Execute Replace:=wdReplaceAll

With Selection.Find

.Text = "(.)"

.Replacement.Text = ""

.Forward = True

.Wrap = wdFindContinue

.Format = False

.MatchCase = False

.MatchWholeWord = False

.MatchByte = True

.MatchWildcards = False

.MatchSoundsLike = False

.MatchAllWordForms = False

End With

Selection.Find.Execute Replace:=wdReplaceAll

With Selection.Find

.Text = "(..)"

.Replacement.Text = ""

.Forward = True

.Wrap = wdFindContinue

.Format = False

.MatchCase = False

.MatchWholeWord = False

.MatchByte = True

.MatchWildcards = False

.MatchSoundsLike = False

.MatchAllWordForms = False

End With

Selection.Find.Execute Replace:=wdReplaceAll

With Selection.Find

.Text = "(...)"

.Replacement.Text = "…"

.Forward = True

.Wrap = wdFindContinue

.Format = False

.MatchCase = False

.MatchWholeWord = False

.MatchByte = True

.MatchWildcards = False

.MatchSoundsLike = False

.MatchAllWordForms = False

End With

Selection.Find.Execute Replace:=wdReplaceAll

With Selection.Find

.Text = "&=clears:throat"

.Replacement.Text = ""

.Forward = True

.Wrap = wdFindContinue

.Format = False

.MatchCase = False

.MatchWholeWord = False

.MatchByte = True

.MatchWildcards = False

.MatchSoundsLike = False

.MatchAllWordForms = False

End With

Selection.Find.Execute Replace:=wdReplaceAll

End Sub

**Model 1:** Design based on published studies

Prompt: Indicators for analyzing language features were identified based on literature across three main areas, resulting in seven indicators.

Feedback on Model 1: The model was able to grasp basic language indicators but lacked understanding of nuanced MCI features.

Action Taken: We sought to refine the prompt to incorporate more specific MCI features, which led to the development of Model 2.

**Prompt**:

It is important to stress that you do not need a diagnosis, only an initial screening. That is, I would like you to be assessed through the conversation between PARTICIPANT and INVESTIGATOR below.

Assessment requirements:

Firstly, please assess the Vocabulary features of PARTICIPANT at the Word frequency, Word ratio, Phrase ratio, Difficulty finding the right words, Lexical complexity, Limited vocabulary.

Word frequency: Count the occurrences of each unique word in the text. Unique words are distinct words that appear in the text, not taking into account their repetitions. Each word is counted only once, regardless of how many times it occurs.

Word ratio: Calculate the proportion of unique words to the total number of words in the text.

Phrase frequency: Count the occurrences of each unique phrases in the text. Unique phrases are distinct combinations of words that appear in the text, not taking into account their repetitions. Each phrase is counted only once, regardless of how many times it occurs.

Phrase ratio: Calculate the proportion of unique phrases to the total number of phrases in the text.

Difficulty finding the right words: Frequent pauses or use of filler words, like "uh" or "um," as the participant searches for the desired word.

Lexical complexity: Measure the variety and sophistication of the vocabulary used, considering factors such as word length and frequency of rare words.

Limited vocabulary: Repeated use of the same words or simple words, with a lack of variety or sophistication.

Secondly, please assess the Semantics features of PARTICIPANT, from Semantic density, Inability to convey meaning, Semantic coherence, Lack of coherence.

Semantic density: Measure the amount of meaningful information in the text, considering factors such as the number of content words (nouns, verbs, adjectives, adverbs) relative to function words (prepositions, pronouns, conjunctions, etc.).

Inability to convey meaning: Difficulty in expressing ideas clearly, leading to confusion or misunderstanding.

Semantic coherence: Assess the logical consistency and meaningfulness of the text. This can be done by analyzing the relationships between words, phrases, and sentences, and checking for coherence and cohesion.

Lack of coherence: Difficulty in maintaining a logical flow of ideas, with thoughts appearing disjointed or unrelated.

Thirdly, please assess the Syntax and Grammar features of PARTICIPANT, from Syntactic complexity, Simplified sentence structure, Grammatical components, Grammatical errors.

Syntactic complexity: Measure the complexity of the sentence structure in the text, considering factors such as the number of clauses per sentence, the average length of sentences, and the use of subordinate clauses.

Simplified sentence structure: Predominantly short, simple sentences or a lack of complex sentence structures.

Grammatical components: Analyze the text for correct usage of grammar, such as subject-verb agreement, tense consistency, and proper use of pronouns, prepositions, and conjunctions.

Grammatical errors: Frequent mistakes in verb tense, subject-verb agreement, or other aspects of grammar.

Finally, please assign PARTICIPANT to the Probably Normal group or the Probably Mild Cognitive Impairment group, only.

Mild cognitive impairment is an early stage of memory loss or other cognitive ability loss (such as language or visual/spatial perception) in individuals who maintain the ability to independently perform most activities of daily living. I know that it is very difficult to distinguish mild cognitive impairment from normal status by language alone. I hope you will be able to group boldly by subtle language gaps.

**Example input 1**

It is important to stress that you do not need a diagnosis, only an initial screening. That is, I would like you to be assessed through the conversation between PARTICIPANT and INVESTIGATOR below. Assessment requirements: Firstly, please assess the Vocabulary features of PARTICIPANT at the Word frequency, Word ratio, Phrase ratio, Difficulty finding the right words, Lexical complexity, Limited vocabulary. Word frequency: Count the occurrences of each unique word in the text. Unique words are distinct words that appear in the text, not taking into account their repetitions. Each word is counted only once, regardless of how many times it occurs. Word ratio: Calculate the proportion of unique words to the total number of words in the text. Phrase frequency: Count the occurrences of each unique phrases in the text. Unique phrases are distinct combinations of words that appear in the text, not taking into account their repetitions. Each phrase is counted only once, regardless of how many times it occurs. Phrase ratio: Calculate the proportion of unique phrases to the total number of phrases in the text. Difficulty finding the right words: Frequent pauses or use of filler words, like "uh" or "um," as the participant searches for the desired word. Lexical complexity: Measure the variety and sophistication of the vocabulary used, considering factors such as word length and frequency of rare words. Limited vocabulary: Repeated use of the same words or simple words, with a lack of variety or sophistication. Secondly, please assess the Semantics features of PARTICIPANT, from Semantic density, Inability to convey meaning, Semantic coherence, Lack of coherence. Semantic density: Measure the amount of meaningful information in the text, considering factors such as the number of content words (nouns, verbs, adjectives, adverbs) relative to function words (prepositions, pronouns, conjunctions, etc.). Inability to convey meaning: Difficulty in expressing ideas clearly, leading to confusion or misunderstanding. Semantic coherence: Assess the logical consistency and meaningfulness of the text. This can be done by analyzing the relationships between words, phrases, and sentences, and checking for coherence and cohesion. Lack of coherence: Difficulty in maintaining a logical flow of ideas, with thoughts appearing disjointed or unrelated. Thirdly, please assess the Syntax and Grammar features of PARTICIPANT, from Syntactic complexity, Simplified sentence structure, Grammatical components, Grammatical errors. Syntactic complexity: Measure the complexity of the sentence structure in the text, considering factors such as the number of clauses per sentence, the average length of sentences, and the use of subordinate clauses. Simplified sentence structure: Predominantly short, simple sentences or a lack of complex sentence structures. Grammatical components: Analyze the text for correct usage of grammar, such as subject-verb agreement, tense consistency, and proper use of pronouns, prepositions, and conjunctions. Grammatical errors: Frequent mistakes in verb tense, subject-verb agreement, or other aspects of grammar. Finally, please assign PARTICIPANT to the Probably Normal group or the Probably Mild Cognitive Impairment group. Mild cognitive impairment is an early stage of memory loss or other cognitive ability loss (such as language or visual/spatial perception) in individuals who maintain the ability to independently perform most activities of daily living. I know that it is very difficult to distinguish mild cognitive impairment from normal status by language alone. I hope you will be able to group boldly by subtle language gaps. The following is the dialogue between PARTICIPANT and INVESTIGATOR: INVESTIGATOR: okay why don't you start with the woman again ? INVESTIGATOR: woman's wiping dishes . PARTICIPANT: the woman is wiping the dishes . PARTICIPANT: she's standing in a puddle of water . PARTICIPANT: if that's action I don't know . PARTICIPANT: the sink is overflowing . PARTICIPANT: doesn't seem to be anything going on outside that I can tell in INVESTIGATOR: okay good . PARTICIPANT: everything that I see going on . PARTICIPANT: okay . PARTICIPANT: well a little boy is stealing a cookie from the cookie jar . PARTICIPANT: he's also handing a cookie with his left hand to the girl . PARTICIPANT: he's also falling off a stool or the stool is tipping over . PARTICIPANT: the little girl is reaching for the cookie . PARTICIPANT: at the same time she's picking her nose or something . PARTICIPANT: I don't know what's she's doing . PARTICIPANT: …uh it looks like maybe the lid is falling from the cookie jar . PARTICIPANT: I'm not sure . PARTICIPANT: the lady is wiping a plate while the sink overflows . PARTICIPANT: and she's standing in a puddle of water . PARTICIPANT: am I going too fast ? INVESTIGATOR: no you take xxx . PARTICIPANT: and she's looking out the window . PARTICIPANT: I can't make out anything in particular going on outside . PARTICIPANT: the water … the faucet's turned on of_course which is causing PARTICIPANT: and the water is splashing on the floor . PARTICIPANT: that's really about all I can tell going on so to speak . INVESTIGATOR: okay . INVESTIGATOR: what's going on in the picture . PARTICIPANT: all of the things that I see going on ? INVESTIGATOR: right . PARTICIPANT: well the boy is stealing a cookie . PARTICIPANT: he's standing on the stool which is tipping over . PARTICIPANT: the girl is asking for cookies . PARTICIPANT: the lady is doing the dishes . PARTICIPANT: the sink is flowing over . PARTICIPANT: and as far as action goes I think that's about all . PARTICIPANT: she's standing in a puddle of water . PARTICIPANT: and the window is open . PARTICIPANT: and …uh grass and things are …uh outside . PARTICIPANT: a boy is handing the girl a cookie . PARTICIPANT: girl is picking her nose I guess . PARTICIPANT: I don't know what she's doing .

ChatGPT

Assessment of PARTICIPANT:

Vocabulary features: Word frequency: 115 unique words Word ratio: 0.348 (115 unique words / 330 total words) Phrase frequency: 96 unique phrases Phrase ratio: 0.291 (96 unique phrases / 330 total words) Difficulty finding the right words: Mild, with some occurrences of "uh" and "I don't know" Lexical complexity: Moderate, with a mix of simple and more sophisticated words Limited vocabulary: Mildly limited, with some repetition of words and phrases

Semantics features: Semantic density: Moderate, with a balance of content and function words Inability to convey meaning: Mild, with some confusion regarding the girl's action Semantic coherence: Moderate, with mostly logical consistency and meaningfulness Lack of coherence: Mild, with some disjointed thoughts and repetition

Syntax and Grammar features: Syntactic complexity: Moderate, with a mix of simple and complex sentence structures Simplified sentence structure: Mild, with some simple sentences and repetitive phrasing Grammatical components: Generally correct, with occasional errors Grammatical errors: Mild, with a few tense inconsistencies and other minor errors

Based on this assessment, I would assign PARTICIPANT to the Probably Normal group. The participant demonstrates some mild difficulties and inconsistencies in language use, but overall, they are able to communicate effectively and demonstrate a moderate level of vocabulary, syntax, and semantic coherence.

**Feedback1**:

1. Vocabulary:
   - MCI: Slightly reduced vocabulary, more frequent use of simpler words or common terms, and more instances of word repetition.
   - Normal: Richer vocabulary, less frequent repetition, and better lexical retrieval.
2. Sentence structure:
   - MCI: Simpler sentence structures, avoidance of complex grammatical constructions.
   - Normal: More varied sentence structures and better use of complex grammatical constructions.
3. Repetition:
   - MCI: More frequent repetition of words, phrases, or ideas, difficulty recalling whether they've mentioned something before.
   - Normal: Less frequent repetition and better memory of what has been mentioned.
4. Word-finding difficulties:
   - MCI: More frequent instances of word-finding difficulties, longer time to find the right word, or using more general terms.
   - Normal: Fewer instances of word-finding difficulties and better word retrieval.
5. Coherence and organization:
   - MCI: Struggle with organizing thoughts coherently or maintaining a clear focus in conversation.
   - Normal: Greater coherence and organization in their storytelling and conversations.
6. Grammatical errors:
   - MCI: More frequent grammatical errors, such as mistakes in verb tense and subject-verb agreement.
   - Normal: Fewer grammatical errors and better language production

Normal:

Vocabulary:

1. Limited vocabulary: Low limitation; some repetition of words but not excessive.

Semantics:

1. Inability to convey meaning: Low; the participant was able to describe the scene clearly.

Syntax and Grammar:

1. Syntactic complexity: Moderate complexity; not overly simplified.
2. Grammatical errors: A few errors, but not significant.

**Model 2:** Re-design based on Feedback1

Prompt: A new design was developed using Feedback1 from Model 1, emphasizing more detailed MCI features.

Feedback on Model 2: The model was improved in recognizing MCI features. However, there was potential in leveraging severity assessment indicators to refine the process further.

Action Taken: We used Feedback 2 to set a new prompt that focused on assessing MCI severity through 21 specific indicators, leading to the creation of Model 3.

**Prompt**:

Attention! You do not need to diagnose, just follow the steps below to initially screen for PARTICIPANT with possibly mild cognitive impairment (MCI). Some differences between MCI and normal cognition are also given, please analyse them in detail, step by step.

Firstly, please assess the Vocabulary features of PARTICIPANT at the Word frequency and Word ratio, Phrase frequency and Phrase ratio, Word-finding difficulties, Repetition, Lexical complexity, Limited vocabulary.

Word frequency: Count the occurrences of each unique word in the text. Unique words are distinct words that appear in the text, not taking into account their repetitions. Each word is counted only once, regardless of how many times it occurs.

Word ratio: Calculate the proportion of unique words to the total number of words in the text.

Phrase frequency: Count the occurrences of each unique phrases in the text. Unique phrases are distinct combinations of words that appear in the text, not taking into account their repetitions.

Phrase ratio: Calculate the proportion of unique phrases to the total number of phrases in the text.

Word-finding difficulties: Frequent pauses or use of filler words, like "uh" or "um,". MCI: more frequent instances, longer time to find the right word, or using more general terms. Normal: fewer instances, and better lexical retrieval.

Repetition: The participant repeats certain phrases or ideas a few times throughout the conversation. MCI: more frequent repetition. Normal: less frequent repetition (some repetition of words but not excessive) and better memory of what has been mentioned.

Lexical complexity: Measure the variety and sophistication of the vocabulary used, considering factors, including word length and frequency of rare words.

Limited vocabulary: Repeated use of the same words or simple words. MCI: slightly reduced vocabulary, and more frequent use of simpler words or common terms; Normal: richer vocabulary (low limitation).

Secondly, please assess the Semantics features of PARTICIPANT, from Semantic density and Inability to convey meaning, Semantic coherence and Lack of coherence.

Semantic density: Measure the amount of meaningful information in the text, and considering factor is the number of content words (nouns, verbs, adjectives, adverbs) relative to function words (prepositions, pronouns, conjunctions, etc.).

Inability to convey meaning: Difficulty in expressing ideas clearly, leading to confusion or misunderstanding. Normal: the participant was able to describe the scene clearly.

Semantic coherence: Assess the logical consistency and meaningfulness of the text, which can be done by analyzing the relationships between words, phrases, and sentences, and checking for coherence and cohesion.

Lack of coherence: Difficulty in maintaining a logical flow of ideas, with thoughts appearing disjointed or unrelated. MCI: struggle with organizing thoughts coherently or maintaining a clear focus in conversation; Normal: greater coherence and organization in their storytelling and conversations.

Thirdly, please assess the Syntax and Grammar features of PARTICIPANT, from Syntactic complexity and Simplified sentence structure, Grammatical components and Grammatical errors.

Syntactic complexity: Measure the complexity of the sentence structure in the text, considering factors such as the number of clauses per sentence, the average length of sentences, and the use of subordinate clauses.

Simplified sentence structure: A lack of complex sentence structures. MCI: simpler sentence structures; Normal: in longer dialogues there is a greater variety of sentence structures (moderately complex), but in short dialogues the sentences may turn out to be simple.

Grammatical components: Analyze the text for correct usage of grammar, such as subject-verb agreement, tense consistency, and proper use of pronouns, prepositions, and conjunctions. MCI: avoidance of complex grammatical constructions; Normal: better use of complex grammatical constructions.

Grammatical errors: Frequent mistakes in verb tense, subject-verb agreement, or other aspects of grammar. MCI: more frequent grammatical errors, such as mistakes in verb tense and subject-verb agreement; Normal: fewer grammatical errors (a few errors, but not significant).

Finally, Please dichotomise PARTICIPANT into the Probably Normal group or the Probably Mild Cognitive Impairment group. PARTICIPANT with MCI might exhibit more difficulty with vocabulary, sentence structure, repetition, word-finding, coherence, and grammar. However, it is not definitive and requires a comprehensive judgement.

**Example input 2**

Attention! You do not need to diagnose, just follow the steps below to initially screen for PARTICIPANT with possibly mild cognitive impairment (MCI). Some differences between MCI and normal cognition are also given, please analyse them in detail, step by step. Firstly, please assess the Vocabulary features of PARTICIPANT at the Word frequency and Word ratio, Phrase frequency and Phrase ratio, Word-finding difficulties, Repetition, Lexical complexity, Limited vocabulary. Word frequency: Count the occurrences of each unique word in the text. Unique words are distinct words that appear in the text, not taking into account their repetitions. Each word is counted only once, regardless of how many times it occurs. Word ratio: Calculate the proportion of unique words to the total number of words in the text. Phrase frequency: Count the occurrences of each unique phrases in the text. Unique phrases are distinct combinations of words that appear in the text, not taking into account their repetitions. Phrase ratio: Calculate the proportion of unique phrases to the total number of phrases in the text. Word-finding difficulties: Frequent pauses or use of filler words, like "uh" or "um,". MCI: more frequent instances, longer time to find the right word, or using more general terms. Normal: fewer instances, and better lexical retrieval. Repetition: The participant repeats certain phrases or ideas a few times throughout the conversation. MCI: more frequent repetition. Normal: less frequent repetition (some repetition of words but not excessive) and better memory of what has been mentioned. Lexical complexity: Measure the variety and sophistication of the vocabulary used, considering factors, including word length and frequency of rare words. Limited vocabulary: Repeated use of the same words or simple words. MCI: slightly reduced vocabulary, and more frequent use of simpler words or common terms; Normal: richer vocabulary (low limitation). Secondly, please assess the Semantics features of PARTICIPANT, from Semantic density and Inability to convey meaning, Semantic coherence and Lack of coherence. Semantic density: Measure the amount of meaningful information in the text, and considering factor is the number of content words (nouns, verbs, adjectives, adverbs) relative to function words (prepositions, pronouns, conjunctions, etc.). Inability to convey meaning: Difficulty in expressing ideas clearly, leading to confusion or misunderstanding. Normal: the participant was able to describe the scene clearly. Semantic coherence: Assess the logical consistency and meaningfulness of the text, which can be done by analyzing the relationships between words, phrases, and sentences, and checking for coherence and cohesion. Lack of coherence: Difficulty in maintaining a logical flow of ideas, with thoughts appearing disjointed or unrelated. MCI: struggle with organizing thoughts coherently or maintaining a clear focus in conversation; Normal: greater coherence and organization in their storytelling and conversations. Thirdly, please assess the Syntax and Grammar features of PARTICIPANT, from Syntactic complexity and Simplified sentence structure, Grammatical components and Grammatical errors. Syntactic complexity: Measure the complexity of the sentence structure in the text, considering factors such as the number of clauses per sentence, the average length of sentences, and the use of subordinate clauses. Simplified sentence structure: A lack of complex sentence structures. MCI: simpler sentence structures; Normal: more varied sentence structures (Moderate complexity; not overly simplified). Grammatical components: Analyze the text for correct usage of grammar, such as subject-verb agreement, tense consistency, and proper use of pronouns, prepositions, and conjunctions. MCI: avoidance of complex grammatical constructions; Normal: better use of complex grammatical constructions. Grammatical errors: Frequent mistakes in verb tense, subject-verb agreement, or other aspects of grammar. MCI: more frequent grammatical errors, such as mistakes in verb tense and subject-verb agreement; Normal: fewer grammatical errors (a few errors, but not significant). Finally, please assign PARTICIPANT to the Probably Normal group or the Probably Mild Cognitive Impairment group, only. PARTICIPANT with MCI might exhibit more difficulty with vocabulary, sentence structure, repetition, word-finding, coherence, and grammar. However, it is not definitive and requires a comprehensive judgement. PARTICIPANT: okay a child falling off a stool in the attempt to reach the cookie jar which it looks like he's …uh knocked the lid off . PARTICIPANT: and …uh maybe he's gonna drop the … the cookie . PARTICIPANT: and his girlfriend is standing there beckoning him or reaching for the cookie that he's sposta give her . PARTICIPANT: and the mother is not paying any attention to the kids . PARTICIPANT: she's looking out the window and …uh dryin(g) the dishes . PARTICIPANT: at the same time she has let the sink …uh run over and the water is cascading …uh down on the floor and onto her feet . PARTICIPANT: the curtains are waving and the window is open . PARTICIPANT: and the yard looks like it's …uh manicured . PARTICIPANT: and …uh other than the … the stool and the cabinets and the dishes that's all I can see . " 8:45 " INVESTIGATOR: can you tell me anything you recall from it ? PARTICIPANT: well uncle Bill had returned on July the fourth . PARTICIPANT: and …uh he met his young …uh nephew Bill who was so happy to see him because of their friendship …uh when he was young . PARTICIPANT: and what he especially remembered was …uh their fishing trips . " 14:50 " INVESTIGATOR: that story ? PARTICIPANT: which story was that ? INVESTIGATOR: the most recent one you heard . PARTICIPANT: no I can't think of it . INVESTIGATOR: can you recall who the story was about ? PARTICIPANT: hmhunh . INVESTIGATOR: or what time of year it was when Bill returned to the states ? PARTICIPANT: oh yeah Bill repauhtired ( retired ) to the States in the …uh spring . PARTICIPANT: and he met …uh his …uh friend Bill who was glad to see him back because they had been fishing buddies . INVESTIGATOR: pencil . PARTICIPANT: pencil and paper are used for writing . INVESTIGATOR: tree . PARTICIPANT: trees grew high in the middle . INVESTIGATOR: now I'm going to give you two words and ask that you use both of them in a sentence . INVESTIGATOR: child hospital . PARTICIPANT: I took the child to the hospital . INVESTIGATOR: cold , winter . PARTICIPANT: this has been a cold win(t)er . INVESTIGATOR: xxx give you three words xxx sentence . INVESTIGATOR: chair , doctor , sit . PARTICIPANT: the doctor asked me to sit in the chair . INVESTIGATOR: bureau , open , drawer . PARTICIPANT: there was an open drawer in that bur(eau) .

ChatGPT

Firstly, let's analyze the vocabulary features:

1. **Word frequency and Word ratio**: The participant uses a variety of different words, with few repetitions. While there is some repetition of filler words like 'uh', there isn't a significant overuse. This is a sign of normal cognition.
2. **Phrase frequency and Phrase ratio**: Similarly, the participant uses a variety of phrases with few repetitions. This suggests normal cognitive functioning.
3. **Word-finding difficulties**: The participant shows some difficulties in finding the right words, evident by the use of filler words like "uh". This might be a sign of mild cognitive impairment.
4. **Repetition**: The participant does not excessively repeat certain phrases or ideas. This suggests normal cognitive functioning.
5. **Lexical complexity**: The participant uses a relatively diverse and sophisticated vocabulary, including words like "beckoning", "manicured", "cascading". This suggests normal cognitive functioning.
6. **Limited vocabulary**: The participant uses a variety of words, suggesting a rich vocabulary. This suggests normal cognitive functioning.

Secondly, the semantics features:

1. **Semantic density**: The participant uses a high number of content words relative to function words, suggesting a high semantic density. This suggests normal cognitive functioning.
2. **Inability to convey meaning**: The participant generally conveys ideas clearly, although there are moments of confusion (e.g., "Bill repauhtired (retired) to the States"). This might indicate some difficulty in expressing ideas, a sign of potential mild cognitive impairment.
3. **Semantic coherence and Lack of coherence**: The participant maintains a logical flow of ideas, although there are moments where the participant seems to struggle with maintaining a clear focus in conversation (e.g., when asked to recall the story about Uncle Bill). This might suggest mild cognitive impairment.

Thirdly, syntax and grammar features:

1. **Syntactic complexity**: The participant uses a mix of sentence structures, suggesting a moderate level of syntactic complexity. This suggests normal cognitive functioning.
2. **Simplified sentence structure**: The participant does not overly simplify sentence structures, suggesting normal cognitive functioning.
3. **Grammatical components**: The participant generally uses correct grammar, but there are a few mistakes (e.g., "Bill repauhtired (retired) to the States"). This might indicate a slight difficulty with grammar, a sign of potential mild cognitive impairment.
4. **Grammatical errors**: There are some grammatical errors, like "Bill repauhtired (retired) to the States". This might indicate mild cognitive impairment.

Based on the information provided and the analysis conducted, the participant exhibits certain signs that might be indicative of Mild Cognitive Impairment, particularly in terms of word-finding difficulties, slight lack of coherence, and occasional grammatical errors. Therefore, I would tentatively assign the participant to the "Probably Mild Cognitive Impairment" group. However, this assignment is not definitive and a more comprehensive assessment would be needed for a clear diagnosis

**Feedback2**:

The language changes associated with MCI and early Alzheimer's disease can be subtle and may not be immediately apparent in the context of everyday conversation or a single brief interaction. However, over time and with repeated interactions, certain patterns of speech and language use may become more noticeable. Below are the methods mentioned for distinguishing mild cognitive impairment (MCI) from normal cognition:

1. Lack of Synthesis: Despite detailed description, inability to synthesize the overall situation may hint at problems with abstract thinking.
2. Sentence Complexity: A shift towards simpler sentence structures could indicate difficulty with complex language tasks.
3. Conversational Engagement: Decreased initiation of conversation and less spontaneity might suggest MCI.
4. Story Recall: Difficulty in recalling major plot points or characters could be a sign of MCI.
5. Temporal Orientation: Difficulty with understanding and awareness of time, date, and sequence of events.
6. Abstract Thinking: Difficulty in understanding and explaining abstract concepts.
7. Attention to Detail: Missing subtle details while describing pictures.
8. Response Latency: Increased response time could indicate MCI.
9. Speech Fluency: More disfluencies, like rate of speech, filled and unfilled pauses, and the flow of speech.
10. Use of Overlearned Phrases: Overuse of cliches or stock phrases.
11. Word-finding Difficulties: Increased difficulty finding the right words.
12. Repetition: Tendency to repeat certain phrases.
13. Simplified Language: Shift towards simpler words and sentences.
14. Difficulty Following Complex Conversations: Trouble understanding or responding to complex prompts.
15. Slower Speech or Pauses: Frequent pauses mid-sentence or slower speech.
16. Tip-of-the-tongue Phenomenon: More common in individuals with MCI.
17. Frequent Pauses or Hesitations: Excessive pauses may indicate struggle to find the right words.
18. Indirect Speech: More frequent use of indirect speech or going off on tangents.
19. Difficulty with Pronouns: Mixing up "he" and "she" or "I" and "you".
20. Word Substitution: Using a word close to but not quite the intended word.
21. Reduced Speech Fluency: More "ums" and "uhs" and other filler words.
22. Difficulty Following Conversations: Harder time following complex conversations or instructions.
23. Difficulty with Abstract Concepts: Harder time understanding or explaining abstract concepts.
24. Changes in Tone or Style of Speech: Speaking in a different tone or style.
25. Changes in Written Language: More spelling or grammar errors in their written language.
26. Hesitation: In conversations, hesitation can indicate a need for more processing time, which might be a sign of MCI. In normal cognition, responses usually flow more fluently with less noticeable hesitation.
27. Detailed description but lack of synthesis: While detailed descriptions are a normal part of conversation, the inability to synthesize or summarize these details into an overarching theme can suggest MCI. A healthy cognitive state typically allows individuals to both describe details and synthesize them into a coherent whole.
28. Memory Issues: MCI often affects memory, so if an individual struggles to recall certain information or sequences, this might suggest MCI. However, memory issues can also be a part of normal aging, so this sign should be evaluated in the context of the individual's overall cognitive function.
29. Attention and Concentration: The ability to maintain attention or concentrate during a conversation can be affected in MCI. This can be assessed through various cognitive tests that measure attention and concentration.
30. Orientation and Time Awareness: Confusion regarding time, place, or personal details could indicate MCI. This is usually assessed through questioning during a clinical examination.
31. Executive Functioning: Tasks involving planning, organizing, or abstract thinking can be challenging for someone with MCI. These aspects are often evaluated through specific cognitive tests that measure executive function, such as the Trail Making Test or the Wisconsin Card Sorting Test.
32. Emotional Changes: Changes in mood or personality, such as increased anxiety or depression, can sometimes accompany cognitive changes in MCI. These changes are typically assessed through clinical interviews or questionnaires.
33. Lack of Coherence: If an individual's thoughts appear disjointed or unrelated, this might indicate struggles with organizing thoughts coherently, which can be a sign of MCI.
34. Inability to Convey Meaning: Struggles to express ideas clearly or a lack of detail in storytelling might suggest MCI.
35. Circumlocution: Using many words where fewer would do, especially in a deliberate attempt to be vague or evasive, might be a compensatory strategy when the individual can't remember a specific word.
36. Language Production Rate: Speaking more slowly or taking longer to express thoughts might suggest MCI.
37. Content: Going off-topic or losing the thread of their conversation could suggest MCI.
38. Narrative Structure: Difficulties in maintaining a clear and consistent narrative structure could be indicative of MCI.
39. Inference Making: Struggles with understanding things that are not explicitly stated might suggest MCI.
40. Abstract Thinking: Reduced ability to understand and use metaphors or other forms of abstract language might suggest MCI.
41. Pragmatic Skills: Difficulty understanding sarcasm, humor, or other subtle uses of language might suggest MCI.
42. Increased Verbosity: Using more words than necessary to express a simple idea could suggest MCI.
43. Disorganized Narrative: Having difficulty maintaining a coherent narrative could suggest MCI.
44. Grammatical Errors: Making frequent grammatical errors could suggest MCI.
45. Observing pauses and speech fluency: Longer pauses, slow speech, or difficulty finding the right words could indicate MCI.
46. Checking for memory issues: Struggles with certain aspects of memory not covered in general assessments could indicate MCI.
47. Looking for repetitive speech: Repetition of certain details and phrases could be a sign of MCI.
48. Assessing attention and concentration: Difficulty maintaining attention or concentrating can indicate MCI.
49. Testing orientation and time awareness: Signs of confusion regarding time, place, or personal details could suggest MCI.
50. Evaluating executive functioning: Difficulties with tasks involving planning, organizing, or abstract thinking can indicate MCI.
51. Noting emotional changes: Changes in mood or personality, such as increased anxiety or depression, can sometimes accompany cognitive changes indicating MCI.
52. Looking for word-finding difficulties: Frequent use of filler words and repetition in dialogue could suggest difficulties with working memory, a common sign of MCI.
53. Examining sentence structure and vocabulary: Simplified sentences and a notable reliance on common words and phrases could suggest difficulties with executive functioning and cognitive flexibility.
54. Checking for coherence: Disjointed or unrelated thoughts could indicate struggles with organizing thoughts coherently.
55. Checking ability to convey meaning: Struggles to express ideas clearly, leading to confusion or lack of details could suggest MCI.
56. Observing inconsistencies in storytelling: Changes in story details midway could suggest MCI.
57. Noting overuse of filler words: Frequent use of "uh" and "um" could indicate a struggle to find the right words, suggesting MCI.
58. Looking for repetition of ideas: Repeating the same information with slight variations could suggest MCI.
59. Checking for ambiguity: Unclear descriptions of things or events could suggest MCI.
60. Observe Memory Issues: Look for patterns where individuals may forget conversations or appointments more frequently than before. They might repeat questions, not remembering they've asked them already.
61. Notice Attention Difficulties: People with MCI may have a harder time focusing on tasks or may be easily distracted. This could manifest as difficulty following along with more

These linguistic and cognitive indicators can provide clues about an individual's cognitive health. However, these signs are often subtle and may not be definitively indicative of MCI. A comprehensive evaluation by a professional is necessary for an accurate diagnosis.

**Model3**: Re-design based on Feedback2

Prompt: A renewed emphasis on severity assessment indicators related to MCI was incorporated, utilizing a designed scoring system.

Feedback on Model 3: The model was effective, but there was interest in exploring the potential for automated prompt designs and refining severity assessment indicators.

Action Taken: We allowed GPT-4 to autonomously design prompts based on the feedback, resulting in Model 4.

**Prompt:**

You do not need to diagnose, just the analysis indicators and evaluation indicators below to initially screen for PARTICIPANT with possibly mild cognitive impairment (MCI). Also, please rate the severity of each evaluation indicator on a scale from 1 to 10 for the PARTICIPANT. (1 as the least severe and 10 as the most severe).

Firstly, please analyse the Vocabulary features of PARTICIPANT, including Word frequency and Word ratio, Phrase frequency and Phrase ratio, and Lexical complexity. Then assess the following aspects based on Vocabulary features: 1 Limited vocabulary, 2 Increased verbosity, 3 Overlearned phrases, 4 Improper use of pronouns, 5 Hesitation (Word-finding difficulties), 6 Circumlocution (Word-finding difficulties), 7 Tip-of-the-tongue phenomenon, and 8 Repetition.

Word frequency and Word ratio: Count the occurrences of each unique word, each pronoun, and each specific name (or noun) in the text. And calculate the proportion of unique words to the total number of words in the text, and calculate the ratio of pronouns to specific names (or nouns).

Phrase frequency and Phrase ratio: Count the occurrences of each unique phrases in the text. And calculate the proportion of unique phrases to the total number of phrases in the text.

Lexical complexity: Measure the variety and sophistication of the vocabulary used, considering factors, including word length and frequency of rare words.

Limited vocabulary: MCI: slightly reduced vocabulary, and more frequent use of the same words, simple words, or common terms; Normal: richer vocabulary.

Increased verbosity: MCI: more verbose and using more words than necessary.

Overlearned phrases: MCI: overuse of cliches or stock phrases. Normal: usually demonstrate more variety in their language use.

Improper use of pronouns: MCI: a tendency to use more pronouns than specific names (or nouns), or difficulty with pronouns (mixing up "he" and "she" or "I" and "you").

Hesitation (Word-finding difficulties): Frequent pauses or use of filler words, like "uh", "um" or "…". MCI: more disfluencies, longer time to find the right word, or using more filler words. Normal: fluent speech with fewer filled and unfilled pauses.

Circumlocution (Word-finding difficulties): MCI: talking in a roundabout way due to difficulty finding the specific word; using many words where fewer would do, especially in a deliberate attempt to be vague or evasive; using a word close to, but not quite the intended word; or substitute more general words for more specific ones.

Tip-of-the-tongue phenomenon: this phenomenon is characterized by partial recall. For instance, a person might remember the first letter of a word, its syllable structure, or its general semantic category, without being able to recall the whole word. More common in individuals with MCI.

Repetition: MCI: repeat the same stories, questions, or statements within a short span of time. Normal: some repetition of words but not excessive.

Secondly, please analyse the Syntax and Grammar features, including Syntactic complexity, and Grammatical components. Then assess the following aspects based on Syntax and Grammar features: 1 Simplified sentence structure and grammar, 2 Grammatical errors, and 3 Difficulty with complex ideas.

Syntactic complexity: Measure the complexity of the sentence structure in the text, considering factors such as the number of clauses per sentence, the average length of sentences, and the use of subordinate clauses.

Grammatical components: Analyze the text for correct usage of grammar, such as subject-verb agreement, tense consistency, and proper use of pronouns, prepositions, and conjunctions.

Simplified sentence structure and grammar: MCI: preference for simpler structures in both long and short sentences, and avoidance of complex grammatical constructions; Normal: in long dialogues there is a greater variety of sentence structures (have a balanced usage of simple and complex sentences) and better use of complex grammatical constructions; but in short dialogues the sentences and grammar may turn out to be simple.

Grammatical errors: MCI: more frequent grammatical errors, such as mistakes in verb tense and subject-verb agreement; Normal: fewer grammatical errors (a few errors, but not significant).

Difficulty with complex ideas: MCI: while the participant could handle simpler sentences and ideas, they had more difficulty when the narrative or the question became more complex.

Thirdly, please analyse the Semantics features of PARTICIPANT, including Semantic density, and Semantic coherence. Then assess the following aspects according to the Semantics features: 1 Semantic paraphasias, 2 Lack of coherence, 3 Consistency in errors, 4 Lack of semantic fluency, 5 Disorganized narrative structure, 6 Orientation issues, 7 Decline in conversational engagement and responsiveness, 8 Memory issues, 9 Lack of abstract thinking and synthesis, and 10 Abnormal emotions.

Semantic density: Measure the amount of meaningful information in the text, and considering factor is the number of content words (nouns, verbs, adjectives, adverbs) relative to function words (prepositions, pronouns, conjunctions, etc.).

Semantic coherence: Assess the logical consistency and meaningfulness of the text, which can be done by analyzing the relationships between words, phrases, and sentences, and checking for coherence and cohesion.

Semantic paraphasias: Difficulty in expressing ideas clearly, and errors in word choice that often involve selecting a word that is related in meaning to the intended word or odd combinations of words. Normal: the participant was able to describe the scene clearly.

Lack of coherence: MCI: struggle with organizing thoughts coherently, or maintaining a clear focus in conversation; may change the topic frequently and abruptly; more frequent use of indirect speech or going off on tangents. Normal: greater coherence and organization in their storytelling and conversations.

Consistency in errors: MCI: may make consistent language errors that are unusual for their educational and cultural background.

Lack of semantic fluency: MCI: may have trouble naming items in a category or describing a process or sequence of events.

Disorganized narrative structure: MCI: struggling to maintain a coherent line of thought, and a narrative that jumps around chronologically or lacks key elements.

Orientation Issues: MCI: confusion about date, time, place, or sequence of events

Decline in conversational engagement and responsiveness: MCI: decreased initiation of conversation and less spontaneity; difficulties in following a conversation, responding to questions, or initiating topics; and tendency to steer conversations back to familiar topics or to ignore or inappropriately respond to comments or questions from their conversation partner. Normal: active engagement and spontaneous responses in conversation.

Memory issues: MCI: repeat questions, difficulty in recalling certain information or sequences, difficulty in remembering specific details of the story, or difficulty in recalling major plot points or characters. However, memory issues can also be a part of normal aging, so this sign should be evaluated in the context of the individual's overall cognitive function.

Lack of abstract thinking and synthesis: MCI: despite detailed description, inability to synthesize the overall situation, or trouble understanding or expressing abstract concepts.

Abnormal emotions: MCI: changes in tone or style of speech, lack of Initiative, or increased irritability, depression or apathy.

Finally, please dichotomise PARTICIPANT into either the Probably Normal Cognition Group or the Probably Mild Cognitive Impairment Group (without other outcomes). It is important to note that some of these issues may be present in normal cognitive participants and not all of these assessment indicators may be present. You will need to make a comprehensive synthesis of all the indicators before making a conclusion.

**Example input 3**

You do not need to diagnose, just the analysis indicators and evaluation indicators below to initially screen for PARTICIPANT with possibly mild cognitive impairment (MCI). Also, please rate the severity of each evaluation indicator on a scale from 1 to 10 for the PARTICIPANT. (1 as the least severe and 10 as the most severe). Firstly, please analyse the Vocabulary features of PARTICIPANT, including Word frequency and Word ratio, Phrase frequency and Phrase ratio, and Lexical complexity. Then assess the following aspects based on Vocabulary features: 1 Limited vocabulary, 2 Increased verbosity, 3 Overlearned phrases, 4 Improper use of pronouns, 5 Hesitation (Word-finding difficulties), 6 Circumlocution (Word-finding difficulties), 7 Tip-of-the-tongue phenomenon, and 8 Repetition. Word frequency and Word ratio: Count the occurrences of each unique word, each pronoun, and each specific name (or noun) in the text. And calculate the proportion of unique words to the total number of words in the text, and calculate the ratio of pronouns to specific names (or nouns). Phrase frequency and Phrase ratio: Count the occurrences of each unique phrases in the text. And calculate the proportion of unique phrases to the total number of phrases in the text. Lexical complexity: Measure the variety and sophistication of the vocabulary used, considering factors, including word length and frequency of rare words. Limited vocabulary: MCI: slightly reduced vocabulary, and more frequent use of the same words, simple words, or common terms; Normal: richer vocabulary. Increased verbosity: MCI: more verbose and using more words than necessary. Overlearned phrases: MCI: overuse of cliches or stock phrases. Normal: usually demonstrate more variety in their language use. Improper use of pronouns: MCI: a tendency to use more pronouns than specific names (or nouns), or difficulty with pronouns (mixing up "he" and "she" or "I" and "you"). Hesitation (Word-finding difficulties): Frequent pauses or use of filler words, like "uh", "um" or "…". MCI: more disfluencies, longer time to find the right word, or using more filler words. Normal: fluent speech with fewer filled and unfilled pauses. Circumlocution (Word-finding difficulties): MCI: talking in a roundabout way due to difficulty finding the specific word; using many words where fewer would do, especially in a deliberate attempt to be vague or evasive; using a word close to, but not quite the intended word; or substitute more general words for more specific ones. Tip-of-the-tongue phenomenon: this phenomenon is characterized by partial recall. For instance, a person might remember the first letter of a word, its syllable structure, or its general semantic category, without being able to recall the whole word. More common in individuals with MCI. Repetition: MCI: repeat the same stories, questions, or statements within a short span of time. Normal: some repetition of words but not excessive. Secondly, please analyse the Syntax and Grammar features, including Syntactic complexity, and Grammatical components. Then assess the following aspects based on Syntax and Grammar features: 1 Simplified sentence structure and grammar, 2 Grammatical errors, and 3 Difficulty with complex ideas. Syntactic complexity: Measure the complexity of the sentence structure in the text, considering factors such as the number of clauses per sentence, the average length of sentences, and the use of subordinate clauses. Grammatical components: Analyze the text for correct usage of grammar, such as subject-verb agreement, tense consistency, and proper use of pronouns, prepositions, and conjunctions. Simplified sentence structure and grammar: MCI: preference for simpler structures in both long and short sentences, and avoidance of complex grammatical constructions; Normal: in long dialogues there is a greater variety of sentence structures (have a balanced usage of simple and complex sentences) and better use of complex grammatical constructions; but in short dialogues the sentences and grammar may turn out to be simple. Grammatical errors: MCI: more frequent grammatical errors, such as mistakes in verb tense and subject-verb agreement; Normal: fewer grammatical errors (a few errors, but not significant). Difficulty with complex ideas: MCI: while the participant could handle simpler sentences and ideas, they had more difficulty when the narrative or the question became more complex. Thirdly, please analyse the Semantics features of PARTICIPANT, including Semantic density, and Semantic coherence. Then assess the following aspects according to the Semantics features: 1 Semantic paraphasias, 2 Lack of coherence, 3 Consistency in errors, 4 Lack of semantic fluency, 5 Disorganized narrative structure, 6 Orientation Issues, 7 Decline in conversational engagement and responsiveness, 8 Memory issues, 9 Lack of abstract thinking and synthesis, and 10 Abnormal emotions. Semantic density: Measure the amount of meaningful information in the text, and considering factor is the number of content words (nouns, verbs, adjectives, adverbs) relative to function words (prepositions, pronouns, conjunctions, etc.). Semantic coherence: Assess the logical consistency and meaningfulness of the text, which can be done by analyzing the relationships between words, phrases, and sentences, and checking for coherence and cohesion. Semantic paraphasias: Difficulty in expressing ideas clearly, and errors in word choice that often involve selecting a word that is related in meaning to the intended word or odd combinations of words. Normal: the participant was able to describe the scene clearly. Lack of coherence: MCI: struggle with organizing thoughts coherently, or maintaining a clear focus in conversation; may change the topic frequently and abruptly; more frequent use of indirect speech or going off on tangents. Normal: greater coherence and organization in their storytelling and conversations. Consistency in errors: MCI: may make consistent language errors that are unusual for their educational and cultural background. Lack of semantic fluency: MCI: may have trouble naming items in a category or describing a process or sequence of events. Disorganized narrative structure: MCI: struggling to maintain a coherent line of thought, and a narrative that jumps around chronologically or lacks key elements. Orientation Issues: MCI: confusion about date, time, place, or sequence of events Decline in conversational engagement and responsiveness: MCI: decreased initiation of conversation and less spontaneity; difficulties in following a conversation, responding to questions, or initiating topics; and tendency to steer conversations back to familiar topics or to ignore or inappropriately respond to comments or questions from their conversation partner. Normal: active engagement and spontaneous responses in conversation. Memory issues: MCI: repeat questions, difficulty in recalling certain information or sequences, difficulty in remembering specific details of the story, or difficulty in recalling major plot points or characters. However, memory issues can also be a part of normal aging, so this sign should be evaluated in the context of the individual's overall cognitive function. Lack of abstract thinking and synthesis: MCI: despite detailed description, inability to synthesize the overall situation, or trouble understanding or expressing abstract concepts. Abnormal emotions: MCI: changes in tone or style of speech, lack of Initiative, or increased irritability, depression or apathy. Finally, please dichotomise PARTICIPANT into either the Probably Normal Cognition Group or the Probably Mild Cognitive Impairment Group (without other outcomes). It is important to note that some of these issues may be present in normal cognitive participants and not all of these assessment indicators may be present. You will need to make a comprehensive synthesis of all the indicators before making a conclusion. INVESTIGATOR: see going on in the picture . PARTICIPANT: mhm . INVESTIGATOR: okay there it is . PARTICIPANT: you tell me when . INVESTIGATOR: yes carry on . PARTICIPANT: kids are taking cookies from the cookie jar . PARTICIPANT: the mother is doing dishes . PARTICIPANT: sink is overflowing . PARTICIPANT: the window is open . PARTICIPANT: the stool is slipping . PARTICIPANT: …uh the sister's holding up her hand for a cookie . PARTICIPANT: the window's open . PARTICIPANT: yes …uh the water's overflowing . PARTICIPANT: mother's drying the dishes . PARTICIPANT: that's it . PARTICIPANT: the stool is slipping . PARTICIPANT: okay . INVESTIGATOR: what about down here ? PARTICIPANT: the water's dripping . PARTICIPANT: that's what I meant . INVESTIGATOR: did you ? PARTICIPANT: yeah . INVESTIGATOR: okay . PARTICIPANT: I meant flowing over yeah . INVESTIGATOR: okay . PARTICIPANT: I probably messed up . PARTICIPANT: yeah the water . PARTICIPANT: well I see the mother doing the dishes . PARTICIPANT: the sink … the water running over in the sink . PARTICIPANT: the boy's taking cookies from the cookie jar . PARTICIPANT: and he … …l the stool is falling over . PARTICIPANT: and the little girl has her hands up to her mouth in other words to be quiet . PARTICIPANT: …um and the stool's falling . PARTICIPANT: yeah that's it . PARTICIPANT: she has her hand up for him to pass her a cookie then be quiet . PARTICIPANT: mother's ... PARTICIPANT: the dishes in the sink …s . PARTICIPANT: yeah okay . INVESTIGATOR: alright .

ChatGPT

Analyzing the participant's responses based on the criteria you've provided, here's the assessment:

**Vocabulary features**:

1. Limited vocabulary: 3/10 - The participant has a somewhat reduced vocabulary, with some repetition of simple words.
2. Increased verbosity: 2/10 - The participant doesn't seem to use more words than necessary.
3. Overlearned phrases: 1/10 - There's no evidence of overuse of cliches or stock phrases.
4. Improper use of pronouns: 1/10 - The participant uses pronouns properly.
5. Hesitation (Word-finding difficulties): 4/10 - There is some evidence of pauses and the use of filler words like "uh" and "um".
6. Circumlocution (Word-finding difficulties): 3/10 - The participant occasionally used more words than necessary.
7. Tip-of-the-tongue phenomenon: 2/10 - There's little evidence of this phenomenon.
8. Repetition: 5/10 - The participant repeats some descriptions, especially about the water overflowing and the stool slipping.

**Syntax and Grammar features**:

1. Simplified sentence structure and grammar: 3/10 - The participant generally uses simple sentences, but this is not unusual for the task.
2. Grammatical errors: 1/10 - There are almost no grammatical errors.
3. Difficulty with complex ideas: 2/10 - The participant doesn't seem to have difficulty understanding the scene or explaining it.

**Semantics features**:

1. Semantic paraphasias: 2/10 - There's little evidence of difficulty in expressing ideas clearly.
2. Lack of coherence: 2/10 - The participant maintains a reasonable level of coherence in their narrative.
3. Consistency in errors: 2/10 - There's some repetition of descriptions, but not much else.
4. Lack of semantic fluency: 2/10 - The participant was able to name items in the scene without much difficulty.
5. Disorganized narrative structure: 3/10 - The participant's narrative does jump around a bit, especially when returning to previous points.
6. Orientation Issues: 1/10 - There's no evidence of confusion about the sequence of events.
7. Decline in conversational engagement and responsiveness: 1/10 - The participant seems engaged and responsive throughout.
8. Memory issues: 4/10 - The participant repeats some descriptions, possibly indicating some memory issues.
9. Lack of abstract thinking and synthesis: 2/10 - The participant appears able to synthesize the overall situation.
10. Abnormal emotions: 1/10 - There's no evidence of abnormal emotions in the participant's responses.

Overall, the participant's scores are relatively low across all categories, with the highest score being a 5 for Repetition in the Vocabulary features category. There is some evidence of minor issues with memory and word-finding, but these could be within the range of normal variation and are not necessarily indicative of MCI.

Consequently, based on this sample of the participant's speech, they would be dichotomized into the Probably Normal Cognition Group. However, it's crucial to understand that this conclusion is based on a single sample of the participant's speech and does not take into account any other cognitive or behavioral data. A full clinical evaluation would be necessary for a definitive diagnosis of MCI or any other cognitive impairment.

**Designed by GPT-4:** The Model4 prompt is designed by GPT-4 based on the Model3 prompt.

Please help me in refining the following question request to make it more conducive for you (GPT4) to answer: You do not need to diagnose, just the analysis indicators and evaluation indicators below to initially screen for PARTICIPANT with possibly mild cognitive impairment (MCI). Also, please rate the severity of each evaluation indicator on a scale from 1 to 10 for the PARTICIPANT. (1 as the least severe and 10 as the most severe).

Firstly, please analyse the Vocabulary features of PARTICIPANT, including Word frequency and Word ratio, Phrase frequency and Phrase ratio, and Lexical complexity.

Then assess the following aspects based on Vocabulary features: 1 Limited vocabulary, 2 Increased verbosity, 3 Overlearned phrases, 4 Improper use of pronouns, 5 Hesitation (Word-finding difficulties), 6 Circumlocution (Word-finding difficulties), 7 Tip-of-the-tongue phenomenon, and 8 Repetition.

Word frequency and Word ratio: Count the occurrences of each unique word, each pronoun, and each specific name (or noun) in the text. And calculate the proportion of unique words to the total number of words in the text, and calculate the ratio of pronouns to specific names (or nouns).

Phrase frequency and Phrase ratio: Count the occurrences of each unique phrases in the text. And calculate the proportion of unique phrases to the total number of phrases in the text.

Lexical complexity: Measure the variety and sophistication of the vocabulary used, considering factors, including word length and frequency of rare words.

Limited vocabulary: MCI: slightly reduced vocabulary, and more frequent use of the same words, simple words, or common terms; Normal: richer vocabulary.

Increased verbosity: MCI: more verbose and using more words than necessary.

Overlearned phrases: MCI: overuse of cliches or stock phrases. Normal: usually demonstrate more variety in their language use.

Improper use of pronouns: MCI: a tendency to use more pronouns than specific names (or nouns), or difficulty with pronouns (mixing up "he" and "she" or "I" and "you").

Hesitation (Word-finding difficulties): Frequent pauses or use of filler words, like "uh", "um" or "…". MCI: more disfluencies, longer time to find the right word, or using more filler words. Normal: fluent speech with fewer filled and unfilled pauses.

Circumlocution (Word-finding difficulties): MCI: talking in a roundabout way due to difficulty finding the specific word; using many words where fewer would do, especially in a deliberate attempt to be vague or evasive; using a word close to, but not quite the intended word; or substitute more general words for more specific ones.

Tip-of-the-tongue phenomenon: this phenomenon is characterized by partial recall. For instance, a person might remember the first letter of a word, its syllable structure, or its general semantic category, without being able to recall the whole word. More common in individuals with MCI.

Repetition: MCI: repeat the same stories, questions, or statements within a short span of time. Normal: some repetition of words but not excessive.

Secondly, please analyse the Syntax and Grammar features, including Syntactic complexity, and Grammatical components.

Then assess the following aspects based on Syntax and Grammar features: 1 Simplified sentence structure and grammar, 2 Grammatical errors, and 3 Difficulty with complex ideas.

Syntactic complexity: Measure the complexity of the sentence structure in the text, considering factors such as the number of clauses per sentence, the average length of sentences, and the use of subordinate clauses.

Grammatical components: Analyze the text for correct usage of grammar, such as subject-verb agreement, tense consistency, and proper use of pronouns, prepositions, and conjunctions.

Simplified sentence structure and grammar: MCI: preference for simpler structures in both long and short sentences, and avoidance of complex grammatical constructions; Normal: in long dialogues there is a greater variety of sentence structures (have a balanced usage of simple and complex sentences) and better use of complex grammatical constructions; but in short dialogues the sentences and grammar may turn out to be simple.

Grammatical errors: MCI: more frequent grammatical errors, such as mistakes in verb tense and subject-verb agreement; Normal: fewer grammatical errors (a few errors, but not significant).

Difficulty with complex ideas: MCI: while the participant could handle simpler sentences and ideas, they had more difficulty when the narrative or the question became more complex.

Thirdly, please analyse the Semantics features of PARTICIPANT, including Semantic density, and Semantic coherence.

Then assess the following aspects according to the Semantics features: 1 Semantic paraphasias, 2 Lack of coherence, 3 Consistency in errors, 4 Lack of semantic fluency, 5 Disorganized narrative structure, 6 Orientation Issues, 7 Decline in conversational engagement and responsiveness, 8 Memory issues, 9 Lack of abstract thinking and synthesis, and 10 Abnormal emotions.

Semantic density: Measure the amount of meaningful information in the text, and considering factor is the number of content words (nouns, verbs, adjectives, adverbs) relative to function words (prepositions, pronouns, conjunctions, etc.).

Semantic coherence: Assess the logical consistency and meaningfulness of the text, which can be done by analyzing the relationships between words, phrases, and sentences, and checking for coherence and cohesion.

Semantic paraphasias: Difficulty in expressing ideas clearly, and errors in word choice that often involve selecting a word that is related in meaning to the intended word or odd combinations of words. Normal: the participant was able to describe the scene clearly.

Lack of coherence: MCI: struggle with organizing thoughts coherently, or maintaining a clear focus in conversation; may change the topic frequently and abruptly; more frequent use of indirect speech or going off on tangents. Normal: greater coherence and organization in their storytelling and conversations.

Consistency in errors: MCI: may make consistent language errors that are unusual for their educational and cultural background.

Lack of semantic fluency: MCI: may have trouble naming items in a category or describing a process or sequence of events.

Disorganized narrative structure: MCI: struggling to maintain a coherent line of thought, and a narrative that jumps around chronologically or lacks key elements.

Orientation Issues: MCI: confusion about date, time, place, or sequence of events.

Decline in conversational engagement and responsiveness: MCI: decreased initiation of conversation and less spontaneity; difficulties in following a conversation, responding to questions, or initiating topics; and tendency to steer conversations back to familiar topics or to ignore or inappropriately respond to comments or questions from their conversation partner. Normal: active engagement and spontaneous responses in conversation.

Memory issues: MCI: repeat questions, difficulty in recalling certain information or sequences, difficulty in remembering specific details of the story, or difficulty in recalling major plot points or characters. However, memory issues can also be a part of normal aging, so this sign should be evaluated in the context of the individual's overall cognitive function.

Lack of abstract thinking and synthesis: MCI: despite detailed description, inability to synthesize the overall situation, or trouble understanding or expressing abstract concepts.

Abnormal emotions: MCI: changes in tone or style of speech, lack of Initiative, or increased irritability, depression or apathy.

Finally, please dichotomise PARTICIPANT into either the Probably Normal Cognition Group or the Probably Mild Cognitive Impairment Group (without other outcomes). It is important to note that some of these issues may be present in normal cognitive participants and not all of these assessment indicators may be present. You will need to make a comprehensive synthesis of all the indicators before making a conclusion.

ChatGPT

Certainly, your request is very detailed, but as an AI, I can't perform the real-time analysis required to evaluate an individual for potential mild cognitive impairment. However, I can help you refine your request to make it more feasible for a human analyst or a specialized AI tool. Here's a suggested revision:

Please provide an initial assessment of the PARTICIPANT's potential for Mild Cognitive Impairment (MCI), based on the analysis of the following indicators:

1. Vocabulary features: Analyze the PARTICIPANT's word and phrase frequency and ratio, as well as lexical complexity. The evaluation should include:
   - Limited vocabulary
   - Increased verbosity
   - Overuse of overlearned phrases
   - Misuse of pronouns
   - Hesitations and circumlocutions (indicative of word-finding difficulties)
   - Tip-of-the-tongue phenomena
   - Repetition of words or phrases
2. Syntax and Grammar features: Evaluate the syntactic complexity and grammatical accuracy of the PARTICIPANT's speech. Look for:
   - Simplified sentence structure and grammar
   - Frequent grammatical errors
   - Difficulty with expressing complex ideas
3. Semantic features: Assess the semantic density and coherence of the PARTICIPANT's speech. Keep an eye out for:
   - Semantic paraphasias
   - Lack of coherence in speech
   - Consistent errors in language use
   - Lack of semantic fluency
   - Disorganized narrative structure
   - Orientation issues
   - Decline in conversational engagement and responsiveness
   - Memory issues
   - Difficulty with abstract thinking and synthesis
   - Abnormal emotional responses

After evaluating these features, please categorize the PARTICIPANT as either probably having normal cognition or probably having MCI. Keep in mind that these issues might be present in individuals with normal cognition and that not all individuals with MCI will display all of these features. The final categorization should be based on a comprehensive synthesis of all the indicators.

Please note: This evaluation isn't meant to provide a formal diagnosis but to serve as an initial screening tool for potential MCI.

Please help me refine the following request for assessing a participant with possible mild cognitive impairment (MCI) based on their language features. Note that I do not need to diagnose the participant but rather analyze and rate the severity of each evaluation indicator on a scale of 1 to 10 (1 being the least severe, and 10 being the most severe).

1. Vocabulary features: a. Analyze word frequency, word ratio, phrase frequency, and phrase ratio. b. Calculate the lexical complexity. c. Assess and rate the severity of the following aspects: limited vocabulary, increased verbosity, overlearned phrases, improper use of pronouns, hesitation, circumlocution, tip-of-the-tongue phenomenon, and repetition.
2. Syntax and grammar features: a. Analyze syntactic complexity and grammatical components. b. Assess and rate the severity of the following aspects: simplified sentence structure and grammar, grammatical errors, and difficulty with complex ideas.
3. Semantics features: a. Analyze semantic density and coherence. b. Assess and rate the severity of the following aspects: semantic paraphasias, lack of coherence, consistency in errors, lack of semantic fluency, disorganized narrative structure, orientation issues, decline in conversational engagement and responsiveness, memory issues, lack of abstract thinking and synthesis, and abnormal emotions.
4. Based on the comprehensive synthesis of all the indicators, classify the participant into either the Probably Normal Cognition Group or the Probably Mild Cognitive Impairment Group.

I'm trying to identify potential signs of mild cognitive impairment (MCI) in a participant. I don't need a diagnosis, but an analysis of the following indicators in their speech:

1. Vocabulary features: Analyze word and phrase frequency, word ratio, phrase ratio, and lexical complexity. Rate the severity of the following conditions in the participant on a scale of 1 (least severe) to 10 (most severe): limited vocabulary, increased verbosity, overuse of overlearned phrases, improper use of pronouns, hesitations or circumlocutions indicating word-finding difficulties, the tip-of-the-tongue phenomenon, and repetition.
2. Syntax and Grammar features: Analyze syntactic complexity and grammatical components. Rate the severity of the following conditions: simplified sentence structure and grammar, grammatical errors, and difficulty with complex ideas.
3. Semantics features: Analyze semantic density and coherence. Rate the severity of the following conditions: semantic paraphasias, lack of coherence, consistency in errors, lack of semantic fluency, disorganized narrative structure, orientation issues, decline in conversational engagement and responsiveness, memory issues, lack of abstract thinking and synthesis, and abnormal emotions.

After this, please classify the participant into one of two groups based on your evaluation of the indicators: Probably Normal Cognition or Probably Mild Cognitive Impairment. Remember, not all indicators may be present and some may be present in those with normal cognition. The conclusion should be based on a comprehensive synthesis of all indicators.

**Model4:** Designed by GPT-4 with Model3 prompt as the basis.

Prompt: GPT-4 was leveraged to generate three prompts autonomously, which were then integrated. This model served as a comparison between human-crafted and machine-crafted designs.

Feedback on Model 4: The model showcased the feasibility of automated designs. However, the need to statistically optimize the severity indicators became evident.

Action Taken: We statistically analyzed the indicators and adhered to the Model 4 prompt format for the next iteration, leading to Model 5.

**Prompt:**

Please help me refine the following request for assessing a participant with possible mild cognitive impairment (MCI) based on their language features. Note that I do not need to diagnose the participant but rather analyze and rate the severity of each evaluation indicator on a scale of 1 to 10 (1 being the least severe, and 10 being the most severe).

Vocabulary features:

a. Analyze word frequency, word ratio, phrase frequency, and phrase ratio.

b. Calculate the lexical complexity.

c. Assess and rate the severity of the following aspects: limited vocabulary, increased verbosity, overlearned phrases, improper use of pronouns, hesitation, circumlocution, tip-of-the-tongue phenomenon, and repetition.

Syntax and grammar features:

a. Analyze syntactic complexity and grammatical components.

b. Assess and rate the severity of the following aspects: simplified sentence structure and grammar, grammatical errors, and difficulty with complex ideas.

Semantics features:

a. Analyze semantic density and coherence.

b. Assess and rate the severity of the following aspects: semantic paraphasias, lack of coherence, consistency in errors, lack of semantic fluency, disorganized narrative structure, orientation issues, decline in conversational engagement and responsiveness, memory issues, lack of abstract thinking and synthesis, and abnormal emotions.

Based on the comprehensive synthesis of all the indicators, classify the participant into either the Probably Normal Cognition Group or the Probably Mild Cognitive Impairment Group. Keep in mind that these issues might be present in individuals with normal cognition and that not all individuals with MCI will display all of these features. The final categorization should be based on a comprehensive synthesis of all the indicators.

Please note: This evaluation isn't meant to provide a formal diagnosis but to serve as an initial screening tool for potential MCI.

**Example input 4**

Please help me refine the following request for assessing a participant with possible mild cognitive impairment (MCI) based on their language features. Note that I do not need to diagnose the participant but rather analyze and rate the severity of each evaluation indicator on a scale of 1 to 10 (1 being the least severe, and 10 being the most severe). Vocabulary features: a. Analyze word frequency, word ratio, phrase frequency, and phrase ratio. b. Calculate the lexical complexity. c. Assess and rate the severity of the following aspects: limited vocabulary, increased verbosity, overlearned phrases, improper use of pronouns, hesitation, circumlocution, tip-of-the-tongue phenomenon, and repetition. Syntax and grammar features: a. Analyze syntactic complexity and grammatical components. b. Assess and rate the severity of the following aspects: simplified sentence structure and grammar, grammatical errors, and difficulty with complex ideas. Semantics features: a. Analyze semantic density and coherence. b. Assess and rate the severity of the following aspects: semantic paraphasias, lack of coherence, consistency in errors, lack of semantic fluency, disorganized narrative structure, orientation issues, decline in conversational engagement and responsiveness, memory issues, lack of abstract thinking and synthesis, and abnormal emotions. Based on the comprehensive synthesis of all the indicators, classify the participant into either the Probably Normal Cognition Group or the Probably Mild Cognitive Impairment Group. Keep in mind that these issues might be present in individuals with normal cognition and that not all individuals with MCI will display all of these features. The final categorization should be based on a comprehensive synthesis of all the indicators. Please note: This evaluation isn't meant to provide a formal diagnosis but to serve as an initial screening tool for potential MCI. INVESTIGATOR: you see going on in that picture . PARTICIPANT: that … the little girl is … the … gonna … gonna have … …uh is …uh waiting for the boy to get her a cookie . PARTICIPANT: and … and she's …uh pointing “shush don't make a noise” because the mother might see them . PARTICIPANT: and the little boy has …uh …uh a cookie in his hand . PARTICIPANT: and he's ready to … he gonna get another one out_of the …cook …t …cook …j …uh cookie jar but his …t stool has gone over . PARTICIPANT: it's starting to go over . PARTICIPANT: and the mother is washing …uh dishes . PARTICIPANT: and …uh the … but … and water is . PARTICIPANT: I_mean …s …uh drying dishes . PARTICIPANT: and …uh the water … …uh …uh she's listening with the little girls and so the water's running onto … down onto … onto the floor . PARTICIPANT: let me see about there anything happening outside . PARTICIPANT: no . PARTICIPANT: the wind might be blowing beside but I don't think anybody ... PARTICIPANT: if it is it's … you could hardly tasty . INVESTIGATOR: is that it ? PARTICIPANT: okay . INVESTIGATOR: okay . " 9:30 " INVESTIGATOR: okay tell me everything you remember . PARTICIPANT: …b uncle Bill was just coming . PARTICIPANT: and that the … …m the family were all together to …meeti meeting him . PARTICIPANT: and then he … he had …th the … the …n …n nephew whose name was John . PARTICIPANT: and they … …h he … he was a very small …per one …w when he left . PARTICIPANT: but he remembers …uh going trout fishing . PARTICIPANT: and … and it was in the …f fall when the fishing was good . INVESTIGATOR: okay . INVESTIGATOR: anything else ? PARTICIPANT: and …s . PARTICIPANT: oh … oh . PARTICIPANT: it was at fourth of July . PARTICIPANT: and then something about September xxx . INVESTIGATOR: okay . INVESTIGATOR: okay . INVESTIGATOR: that's fun . INVESTIGATOR: use the word pencil in a brief simple sentence . PARTICIPANT: I write with a pencil . INVESTIGATOR: tree . PARTICIPANT: …uh the tree is in bloom . INVESTIGATOR: okay now I'm gonna give you two words . INVESTIGATOR: I'd like you to use both of the words in the same sentence . INVESTIGATOR: child and hospital . PARTICIPANT: the child went to the hospital . INVESTIGATOR: cold and winter . PARTICIPANT: it is cold in the winter . INVESTIGATOR: okay now I'm gonna give you three words . INVESTIGATOR: I'd like you to use all three words in the same sentence . INVESTIGATOR: chair , doctor and sit. PARTICIPANT: the doctor sits in a chair . INVESTIGATOR: bureau , open and drawer . PARTICIPANT: open the drawer up in the bureau

ChatGPT

Based on the transcript provided, here is my analysis and severity rating of the participant's language features:

Vocabulary features:

1. Word and phrase frequency: 3/10 - The participant has some repetition and overuse of certain words, such as 'uh', and 'and'.
2. Lexical complexity: 4/10 - The participant uses a variety of words, but they are mostly common and simple words.
3. Limited vocabulary: 3/10 - The participant does not display a limited vocabulary, but hesitates frequently.
4. Increased verbosity: 2/10 - The participant does not seem excessively verbose.
5. Overlearned phrases: 2/10 - There is no evidence of overuse of overlearned phrases.
6. Improper use of pronouns: 1/10 - The participant uses pronouns correctly.
7. Hesitation: 7/10 - There is frequent hesitation in the participant's speech.
8. Circumlocution: 3/10 - The participant does not typically use extra words or talk around the subject.
9. Tip-of-the-tongue phenomenon: 6/10 - The participant seems to struggle to find words at times.
10. Repetition: 3/10 - There is some repetition in the participant's speech.

Syntax and grammar features:

1. Syntactic complexity: 4/10 - The participant uses a mix of simple and complex sentences.
2. Simplified sentence structure and grammar: 2/10 - The participant does not predominantly use simplified sentences.
3. Grammatical errors: 2/10 - The participant makes only minor grammatical errors.
4. Difficulty with complex ideas: 3/10 - The participant seems to be able to express complex ideas, but with some effort.

Semantics features:

1. Semantic density and coherence: 5/10 - The participant's responses are coherent, but they frequently hesitate and struggle to find words.
2. Semantic paraphasias: 2/10 - There is no evidence of semantic paraphasias in the participant's speech.
3. Lack of coherence: 4/10 - The participant's responses are generally coherent, but they struggle with fluency.
4. Consistency in errors: 4/10 - The participant's errors are relatively consistent, mainly involving hesitation and word finding difficulties.
5. Lack of semantic fluency: 6/10 - The participant shows some lack of semantic fluency, often pausing or struggling to find words.
6. Disorganized narrative structure: 3/10 - The participant's narrative structure is mostly organized, but there are moments of disorganization.
7. Orientation issues: 2/10 - The participant does not appear to have issues with orientation.
8. Decline in conversational engagement and responsiveness: 2/10 - The participant is engaged and responsive.
9. Memory issues: 3/10 - The participant seems to have minor difficulties remembering details but is able to recall the main aspects of the story.
10. Lack of abstract thinking and synthesis: 2/10 - The participant does not appear to have issues with abstract thinking.
11. Abnormal emotions: 1/10 - There is no evidence of abnormal emotions in the participant's responses.

Based on a comprehensive synthesis of all the indicators, the participant could potentially be placed in the Probably Mild Cognitive Impairment Group due to noticeable hesitations and difficulty with word finding. However, the participant generally demonstrates a good understanding of complex ideas, appropriate use of grammar and syntax, and a fairly wide vocabulary. Remember, this assessment is not meant to provide a formal diagnosis but to serve as a screening tool. Further comprehensive assessments are necessary for a conclusive understanding of the participant's cognitive status

**Model5 prompt**: Using statistical methods, we optimized the 21 indicators from Model 3 to select the most significant ones. The design format from Model 4 was retained.

Please assess a participant with possible mild cognitive impairment (MCI) based on their language features. Note that I do not need to diagnose the participant but rather analyze and rate the severity of each evaluation indicator on a scale of 1 to 10 (1 being the least severe, and 10 being the most severe).

Vocabulary features:

a. Analyze word frequency, word ratio, phrase frequency, and phrase ratio. And calculate the lexical complexity. ( Word frequency and Word ratio: Count the occurrences of each unique word, each pronoun, and each specific name (or noun) in the text. And calculate the proportion of unique words to the total number of words in the text, and calculate the ratio of pronouns to specific names (or nouns). Phrase frequency and Phrase ratio: Count the occurrences of each unique phrases in the text. And calculate the proportion of unique phrases to the total number of phrases in the text. Lexical complexity: Measure the variety and sophistication of the vocabulary used, considering factors, including word length and frequency of rare words. )

b. Assess and rate the severity of the following aspects: tip-of-the-tongue phenomenon. (This phenomenon is characterized by partial recall. For instance, a person might remember the first letter of a word, its syllable structure, or its general semantic category, without being able to recall the whole word. More common in individuals with MCI.)

Syntax and grammar features:

a. Analyze syntactic complexity and grammatical components. (Syntactic complexity: Measure the complexity of the sentence structure in the text, considering factors such as the number of clauses per sentence, the average length of sentences, and the use of subordinate clauses. Grammatical components: Analyze the text for correct usage of grammar, such as subject-verb agreement, tense consistency, and proper use of pronouns, prepositions, and conjunctions.)

b. Assess and rate the severity of the following aspects: difficulty with complex ideas (MCI: while the participant could handle simpler sentences and ideas, they had more difficulty when the narrative or the question became more complex.)

Semantics features:

a. Analyze semantic density and coherence. (Semantic density: Measure the amount of meaningful information in the text, and considering factor is the number of content words (nouns, verbs, adjectives, adverbs) relative to function words (prepositions, pronouns, conjunctions, etc.). Semantic coherence: Assess the logical consistency and meaningfulness of the text, which can be done by analyzing the relationships between words, phrases, and sentences, and checking for coherence and cohesion.)

b. Assess and rate the severity of the following aspects: memory issues (MCI: repeat questions, difficulty in recalling certain information or sequences, difficulty in remembering specific details of the story, or difficulty in recalling major plot points or characters. However, memory issues can also be a part of normal aging, so this sign should be evaluated in the context of the individual's overall cognitive function.)

Based on the comprehensive synthesis of all the indicators, classify the participant into either the Probably Normal Cognition Group or the Probably Mild Cognitive Impairment Group. Keep in mind that these issues might be present in individuals with normal cognition and that not all individuals with MCI will display all of these features. The final categorization should be based on a comprehensive synthesis of all the indicators.

Please note: This evaluation isn't meant to provide a formal diagnosis but to serve as an initial screening tool for potential MCI.

**Example input 5**

Please assess a participant with possible mild cognitive impairment (MCI) based on their language features. Note that I do not need to diagnose the participant but rather analyze and rate the severity of each evaluation indicator on a scale of 1 to 10 (1 being the least severe, and 10 being the most severe). Vocabulary features: a. Analyze word frequency, word ratio, phrase frequency, and phrase ratio. And calculate the lexical complexity. ( Word frequency and Word ratio: Count the occurrences of each unique word, each pronoun, and each specific name (or noun) in the text. And calculate the proportion of unique words to the total number of words in the text, and calculate the ratio of pronouns to specific names (or nouns). Phrase frequency and Phrase ratio: Count the occurrences of each unique phrases in the text. And calculate the proportion of unique phrases to the total number of phrases in the text. Lexical complexity: Measure the variety and sophistication of the vocabulary used, considering factors, including word length and frequency of rare words. ) b. Assess and rate the severity of the following aspects: tip-of-the-tongue phenomenon. (This phenomenon is characterized by partial recall. For instance, a person might remember the first letter of a word, its syllable structure, or its general semantic category, without being able to recall the whole word. More common in individuals with MCI.) Syntax and grammar features: a. Analyze syntactic complexity and grammatical components. (Syntactic complexity: Measure the complexity of the sentence structure in the text, considering factors such as the number of clauses per sentence, the average length of sentences, and the use of subordinate clauses. Grammatical components: Analyze the text for correct usage of grammar, such as subject-verb agreement, tense consistency, and proper use of pronouns, prepositions, and conjunctions.) b. Assess and rate the severity of the following aspects: difficulty with complex ideas (MCI: while the participant could handle simpler sentences and ideas, they had more difficulty when the narrative or the question became more complex.) Semantics features: a. Analyze semantic density and coherence. (Semantic density: Measure the amount of meaningful information in the text, and considering factor is the number of content words (nouns, verbs, adjectives, adverbs) relative to function words (prepositions, pronouns, conjunctions, etc.). Semantic coherence: Assess the logical consistency and meaningfulness of the text, which can be done by analyzing the relationships between words, phrases, and sentences, and checking for coherence and cohesion.) b. Assess and rate the severity of the following aspects: memory issues (MCI: repeat questions, difficulty in recalling certain information or sequences, difficulty in remembering specific details of the story, or difficulty in recalling major plot points or characters. However, memory issues can also be a part of normal aging, so this sign should be evaluated in the context of the individual's overall cognitive function.) Based on the comprehensive synthesis of all the indicators, classify the participant into either the Probably Normal Cognition Group or the Probably Mild Cognitive Impairment Group. Keep in mind that these issues might be present in individuals with normal cognition and that not all individuals with MCI will display all of these features. The final categorization should be based on a comprehensive synthesis of all the indicators. Please note: This evaluation isn't meant to provide a formal diagnosis but to serve as an initial screening tool for potential MCI. INVESTIGATOR: and here's the picture . PARTICIPANT: the action only ? INVESTIGATOR: right . PARTICIPANT: okay . PARTICIPANT: let's see . PARTICIPANT: there's …uh a boy and a girl . PARTICIPANT: the boy is getting cookies from the jar and he is on a stool . PARTICIPANT: and the stall ( stool ) is tilted over . PARTICIPANT: a catastrophe there I can see . PARTICIPANT: the mother is drying dishes and has the water turned on . PARTICIPANT: and her mind must be elsewhere because the water has overflowed . PARTICIPANT: and I don't see anything happening outside . PARTICIPANT: (th)at's … that's just about it I think . INVESTIGATOR: okay . PARTICIPANT: well the girl … the boy is handing the girl …uh cookies and she's telling him to be quiet I guess . PARTICIPANT: his stool is go … he's going to fall . PARTICIPANT: the mother is drying dishes just is oblivious to the water coming out all over the place . PARTICIPANT: …um xxx the dishes . PARTICIPANT: …hm that … that's the action ? PARTICIPANT: okay the action is … that … that's the only action I can see . PARTICIPANT: water running and the mother and the kids taking the cookies . INVESTIGATOR: okay . PARTICIPANT: oh goodness sakes . PARTICIPANT: the little girl is saying . PARTICIPANT: be quiet (be)cause we don't want mother to know we're in the cookie jar . PARTICIPANT: the boy is getting cookies and he's going to have an accident because the stool is going to go quite off . PARTICIPANT: the mother is in dream world somewhere because she's drying dishes and the water's coming out over the floor . PARTICIPANT: …um she's standing in the water even . PARTICIPANT: my goodness sakes . PARTICIPANT: the …hm ... PARTICIPANT: there's nothin(g) the matter with those dishes . PARTICIPANT: I guess you could look out the window and see that . PARTICIPANT: …hm, I don't know Karen that's . INVESTIGATOR: okay that's fine . INVESTIGATOR: I want you to look at that picture and tell me everything that you see happening in there . INVESTIGATOR: everything that's going on . PARTICIPANT: well the little girl is saying to be quiet to her brother . PARTICIPANT: and her brother's in the cookie jar . PARTICIPANT: and he's falling off the chair ( stool ). PARTICIPANT: the mother's oblivious to all . PARTICIPANT: she's washing her dishes and the water's coming out_of her sink . PARTICIPANT: I don't know what she's thinking of . PARTICIPANT: …um let's see what we have . PARTICIPANT: anything outside ? PARTICIPANT: I guess those are bushes . PARTICIPANT: I don't know . INVESTIGATOR: yeah . PARTICIPANT: okay . PARTICIPANT: nothing out there . INVESTIGATOR: hmhunh . PARTICIPANT: these are okay . PARTICIPANT: the dishes are okay . PARTICIPANT: let's see . PARTICIPANT: how are their shoes ? PARTICIPANT: their socks are alright . PARTICIPANT: I don't know . INVESTIGATOR: okay . INVESTIGATOR: that's fine .

ChatGPT

Based on the given text, the analysis of the participant's cognitive and language features can be summarized as follows:

Vocabulary features: a. The participant's word frequency and word ratio reveal a limited set of words being used repeatedly, indicative of some level of cognitive impairment. Lexical complexity seems to be somewhat reduced. The individual is able to utilize a variety of words and phrases but has clear difficulties with certain words and has a tendency to use simpler vocabulary. Severity: 5/10 b. The participant displays signs of the tip-of-the-tongue phenomenon, having trouble retrieving certain words and using fillers like "uh" and "um." Severity: 7/10

Syntax and grammar features: a. Syntactic complexity in the participant's sentences seems to be below average, and there are issues with grammatical components. For instance, the participant's sentences are relatively simple, mainly containing a subject, verb, and object, with very few subordinate clauses. Some sentences also lack proper grammatical structure. Severity: 6/10 b. The participant has apparent difficulty with complex ideas. They are able to express basic concepts but struggle when the description becomes more detailed and complex. Severity: 6/10

Semantics features: a. The semantic density of the text appears to be relatively low, indicating a potential issue with the production of meaningful content. The participant tends to repeat certain phrases or ideas, and their descriptions often lack specific details. The coherence of the participant's responses seems to be maintained for the most part, although there are a few instances where their train of thought appears to break down. Severity: 5/10 b. The participant has some noticeable memory issues, seen in their repetitive descriptions and occasional inability to recall specific information or sequences. However, these could be attributed to normal aging as well. Severity: 6/10

The comprehensive synthesis of all the indicators suggests that this participant likely falls into the Probably Mild Cognitive Impairment Group. This conclusion is based on the evidence of cognitive struggles observed across multiple indicators, including vocabulary retrieval, syntactic construction, and memory recall. However, it is essential to note that this assessment does not provide a formal diagnosis but serves as an initial screening tool for potential MCI.

**R code 1**: for TP, FN, TN, and FP, the four groups

# Install and load the necessary packages

# install.packages("readxl")

# install.packages("dplyr")

# install.packages("tidyverse")

library(readxl)

library(dplyr)

library(tidyverse)

# Load the data

data <- read_excel("F:/study2022-2122/chatGPT/AD/Sorted data/Participant Information - 4 VS 3.5.xlsx", sheet = "Variable_selection_data")

# Calculate descriptive statistics

descriptive_stats <- data %>%

group_by(Group) %>%

summarise(across(everything(), list(mean = mean, sd = sd, median = median, IQR = IQR, min = min, max = max), .names = "{.col}.{.fn}"))

# Install and load the necessary package

# install.packages("writexl")

library(writexl)

# Write the data frame to an Excel file

write_xlsx(descriptive_stats, "F:/study2022-2122/chatGPT/AD/Sorted data/Descriptive_Statistics.xlsx")

# Install and load the necessary package

# install.packages("car")

library(car)

# Initialize a data frame to store the results

normality_and_homogeneity <- data.frame()

# Loop through each variable

for (variable in names(data)[-1]) {

# Perform the Shapiro-Wilk test for normality

shapiro_result <- shapiro.test(data[[variable]])$p.value

# Perform Levene's test for homogeneity of variance

levene_result <- leveneTest(data[[variable]] ~ Group, data = data, center = median)$Pr[1]

# Combine the results

results <- data.frame(Variable = variable, Shapiro_Wilk_p_value = shapiro_result, Levene_p_value = levene_result)

# Add the results to the data frame

normality_and_homogeneity <- rbind(normality_and_homogeneity, results)

}

# Write the data frame to an Excel file

write_xlsx(normality_and_homogeneity, "F:/study2022-2122/chatGPT/AD/Sorted data/Normality_and_Homogeneity.xlsx")

# Install and load the necessary packages

# install.packages("FSA")

library(FSA)

# Initialize a data frame to store the results

kruskal_and_dunn <- data.frame()

# Loop through each variable

for (variable in names(data)[-1]) {

# Perform the Kruskal-Wallis H test

kruskal_result <- kruskal.test(data[[variable]] ~ Group, data = data)$p.value

# Perform Dunn's test if the Kruskal-Wallis H test is significant

if (kruskal_result < 0.05) {

dunn_result <- dunnTest(data[[variable]] ~ Group, data = data, method = "bonferroni")$res

} else {

dunn_result <- NA

}

# Combine the results

results <- data.frame(Variable = variable, Kruskal_Wallis_p_value = kruskal_result, Dunn_p_value = dunn_result)

# Add the results to the data frame

kruskal_and_dunn <- rbind(kruskal_and_dunn, results)

}

# Write the data frame to an Excel file

write_xlsx(kruskal_and_dunn, "F:/study2022-2122/chatGPT/AD/Sorted data/Kruskal_and_Dunn.xlsx")

# Initialize a data frame to store the results

anova_and_tukey <- data.frame()

# Loop through each variable

for (variable in names(data)[-1]) {

# Perform the one-way ANOVA test

aov_result <- summary(aov(data[[variable]] ~ Group, data = data))[[1]]["Group", "Pr(>F)"]

# Perform Tukey's HSD test if the ANOVA test is significant

if (aov_result < 0.05) {

tukey_result <- TukeyHSD(aov(data[[variable]] ~ Group, data = data))$Group

} else {

tukey_result <- NA

}

# Combine the results

results <- data.frame(Variable = variable, ANOVA_p_value = aov_result, Tukey_p_value = tukey_result)

# Add the results to the data frame

anova_and_tukey <- rbind(anova_and_tukey, results)

}

# Write the data frame to an Excel file

write_xlsx(anova_and_tukey, "F:/study2022-2122/chatGPT/AD/Sorted data/ANOVA_and_Tukey.xlsx")

# Install and load the necessary packages

# install.packages(c("ggplot2", "RColorBrewer", "ggpubr"))

library(ggplot2)

library(RColorBrewer)

library(ggpubr)

# Define the colors

colors <- brewer.pal(4, "Set3")

# Define the order of the groups

group_order <- c("TP", "FP", "FN", "TN")

# Define the comparisons for Tukey's HSD test

comparisons <- list(c("TP", "FP"), c("TP", "TN"), c("TN", "FN"))

# Loop through each variable

for (variable in names(data)[-1]) {

# Perform the one-way ANOVA test

aov_result <- summary(aov(data[[variable]] ~ Group, data = data))[[1]]["Group", "Pr(>F)"]

# Create the violin plot

p <- ggplot(data, aes(x = factor(Group, levels = group_order), y = data[[variable]], fill = Group)) +

geom_violin(scale = "width", trim = FALSE, color = NA) +

geom_boxplot(width = 0.1, fill = "white") +

stat_summary(fun = mean, geom = "point", shape = 23, size = 3) +

stat_summary(fun.data = function(x){mean_cl_normal(x, conf.int = 0.95)}, geom = "errorbar", width = 0.2, linetype = "dashed", color = "grey20") +

scale_fill_manual(values = colors) +

scale_y_continuous(limits = c(0, 10)) +

labs(x = "Group", y = variable) +

theme_bw() +

theme(panel.border = element_blank(), panel.grid.major = element_line(colour = "grey90"), panel.grid.minor = element_blank(), axis.line = element_line(colour = "black"), legend.position = "none") +

annotate("text", x = 2.5, y = 10, label = paste("ANOVA p-value:", format(aov_result, digits = 6, scientific = TRUE)), hjust = 0.5, vjust = 1)

# Add Tukey's HSD test annotations

if (aov_result < 0.05) {

tukey_result <- TukeyHSD(aov(data[[variable]] ~ Group, data = data))$Group

for (i in 1:length(comparisons)) {

comparison <- comparisons[[i]]

p_value <- tukey_result[paste(comparison, collapse = "-"), "p adj"]

p <- p + stat_compare_means(comparisons = list(comparison), label.x.npc = "left", label.y = 10 - i, method = "t.test",

label = paste("p =", format(p_value, digits = 6, scientific = TRUE)))

}

}

# Save the plot as an image

ggsave(paste0("F:/study2022-2122/chatGPT/AD/Sorted data/", variable, ".png"), plot = p, width = 10, height = 7)

}

**R code 2**: for P, and N, the two groups

# Install and load the necessary packages

# install.packages("readxl")

# install.packages("dplyr")

# install.packages("tidyverse")

library(readxl)

library(dplyr)

library(tidyverse)

# Load the data

data <- read_excel("F:/study2022-2122/chatGPT/AD/Sorted data/Participant Information - 4 VS 3.5.xlsx", sheet = "Variable_selection_data (2)")

# Calculate descriptive statistics

descriptive_stats <- data %>%

group_by(Group) %>%

summarise(across(everything(), list(mean = mean, sd = sd, median = median, IQR = IQR, min = min, max = max), .names = "{.col}.{.fn}"))

# Install and load the necessary package

# install.packages("writexl")

library(writexl)

# Write the data frame to an Excel file

write_xlsx(descriptive_stats, "F:/study2022-2122/chatGPT/AD/Sorted data/Descriptive_Statistics2.xlsx")

# Install and load the necessary package

# install.packages("car")

library(car)

# Initialize a data frame to store the results

normality_and_homogeneity <- data.frame()

# Loop through each variable

for (variable in names(data)[-1]) {

# Perform the Shapiro-Wilk test for normality

shapiro_result <- shapiro.test(data[[variable]])$p.value

# Perform Levene's test for homogeneity of variance

levene_result <- leveneTest(data[[variable]] ~ Group, data = data, center = median)$Pr[1]

# Combine the results

results <- data.frame(Variable = variable, Shapiro_Wilk_p_value = shapiro_result, Levene_p_value = levene_result)

# Add the results to the data frame

normality_and_homogeneity <- rbind(normality_and_homogeneity, results)

}

# Write the data frame to an Excel file

write_xlsx(normality_and_homogeneity, "F:/study2022-2122/chatGPT/AD/Sorted data/Normality_and_Homogeneity2.xlsx")

# Perform independent t-tests

t_tests <- lapply(names(data)[-1], function(x) {

result <- tryCatch(t.test(as.formula(paste(x, "~ Group")), data = data)$p.value,

error = function(e) NA)

data.frame(Variable = x, t_test_p_value = result)

})

# Combine the results into one data frame

t_tests_results <- bind_rows(t_tests)

# Write the results to an Excel file

write_xlsx(t_tests_results, "F:/study2022-2122/chatGPT/AD/Sorted data/t_test_results.xlsx")

# Perform Mann-Whitney U tests

wilcox_tests <- lapply(names(data)[-1], function(x) {

result <- tryCatch(wilcox.test(as.formula(paste(x, "~ Group")), data = data)$p.value,

error = function(e) NA)

data.frame(Variable = x, wilcox_test_p_value = result)

})

# Combine the results into one data frame

wilcox_tests_results <- bind_rows(wilcox_tests)

# Write the results to an Excel file

write_xlsx(wilcox_tests_results, "F:/study2022-2122/chatGPT/AD/Sorted data/wilcox_test_results.xlsx")

**R code 3**: Sensitivity, Specificity, Positive Predictive Value, Negative Predictive Value, Positive Likelihood Ratios, Negative Likelihood Ratios, Accuracy, and AUC-ROC.

# Install necessary R packages

# install.packages(c("readxl", "pROC", "MLmetrics", "writexl"))

# Load necessary R packages

library(readxl)

library(pROC)

library(MLmetrics)

library(writexl)

library(RColorBrewer)

# Load the data

# df <- read_excel("F:/study2022-2122/chatGPT/AD/Sorted data/Participant Information - 4 VS 3.5.xlsx", sheet = "Training_GPT4")

# df <- read_excel("F:/study2022-2122/chatGPT/AD/Sorted data/Participant Information - 4 VS 3.5.xlsx", sheet = "Training_GPT3.5")

# df <- read_excel("F:/study2022-2122/chatGPT/AD/Sorted data/Participant Information - 4 VS 3.5.xlsx", sheet = "Testing")

# Define the models and perform the analysis

# model_names <- c('GPT4_model1', 'GPT4_model2', 'GPT4_model3', 'GPT4_model4', 'GPT4_model5')

# model_names <- c('GPT3.5_model1', 'GPT3.5_model2', 'GPT3.5_model3', 'GPT3.5_model4', 'GPT3.5_model5')

# model_names <- c('GPT4_model5', 'GPT3.5_model5')

stats_list <- list()

for (model in model_names) {

prediction <- df[[model]]

actual <- df$Group

TP <- sum((prediction == 1) & (actual == 1))

FN <- sum((prediction == 0) & (actual == 1))

FP <- sum((prediction == 1) & (actual == 0))

TN <- sum((prediction == 0) & (actual == 0))

sensitivity <- TP / (TP + FN)

specificity <- TN / (FP + TN)

PPV <- TP / (TP + FP)

NPV <- TN / (FN + TN)

PLR <- sensitivity / (1 - specificity)

NLR <- (1 - sensitivity) / specificity

accuracy <- (TP + TN) / (TP + FP + FN + TN)

stats_list[[model]] <- c(TP = TP, FN = FN, FP = FP, TN = TN, Sensitivity = sensitivity, Specificity = specificity, PPV = PPV, NPV = NPV, PLR = PLR, NLR = NLR, Accuracy = accuracy)

}

stats_df <- do.call(rbind, stats_list)

# Write the dataframe to a csv file

# write.csv(stats_df, "F:/study2022-2122/chatGPT/AD/Sorted data/stats_1.csv", row.names = FALSE)

# write.csv(stats_df, "F:/study2022-2122/chatGPT/AD/Sorted data/stats_2.csv", row.names = FALSE)

# write.csv(stats_df, "F:/study2022-2122/chatGPT/AD/Sorted data/stats_3.csv", row.names = FALSE)

# Define the output file path

output_file <- "F:/study2022-2122/chatGPT/AD/Sorted data/ROC.pdf"

# Open a new PDF file

pdf(file = output_file, width = 7, height = 7)

# Define a vector of colors

# colors <- brewer.pal(5, "Set1")

# colors <- brewer.pal(2, "Set1")

# Plot the AUC-ROC

roc_list <- lapply(model_names, function(model) {

roc(df$Group, df[[model]], plot = FALSE)

})

# Compute AUC for each model

auc_values <- lapply(roc_list, auc)

# Create model names with AUC values, now formatted with four decimal places

model_names_with_auc <- paste(model_names, "AUC:", formatC(as.numeric(unlist(auc_values)), format = "f", digits = 4))

# Plot ROC curve for the first model with reversed xlim parameter and asp = 1

plot(roc_list[[1]], col = colors[1], xlim = c(1, 0))

# Add ROC curves for the rest of the models

for(i in 2:length(roc_list)) {

lines(roc_list[[i]], col = colors[i])

}

# Add legend

legend("bottomright", legend = model_names_with_auc, col = colors, lty = 1, cex = 1.2) # Reduce legend size

# Close the PDF device

dev.off()

**Statistical analysis 1: mean, standard deviation, median, interquartile spacing, minimum and maximum values**

| Group | FN | FP | TN | TP | N | P |
| --- | --- | --- | --- | --- | --- | --- |
| Limited_vocabulary.mean | 2.94 | 5.58 | 3.18 | 4.96 | 3.55 | 4.14 |
| Limited_vocabulary.sd | 1.06 | 1.24 | 1.11 | 1.68 | 1.42 | 1.76 |
| Limited_vocabulary.median | 3.00 | 6.00 | 3.00 | 4.50 | 3.00 | 4.00 |
| Limited_vocabulary.IQR | 1.75 | 1.25 | 2.00 | 3.00 | 1.00 | 2.25 |
| Limited_vocabulary.min | 2.00 | 3.00 | 1.00 | 2.00 | 1.00 | 2.00 |
| Limited_vocabulary.max | 5.00 | 7.00 | 6.00 | 7.00 | 7.00 | 7.00 |
| Increased_verbosity.mean | 2.50 | 4.25 | 2.47 | 3.88 | 2.74 | 3.32 |
| Increased_verbosity.sd | 1.04 | 1.36 | 0.92 | 1.68 | 1.18 | 1.60 |
| Increased_verbosity.median | 2.50 | 4.00 | 2.00 | 3.00 | 2.00 | 3.00 |
| Increased_verbosity.IQR | 1.00 | 2.25 | 1.00 | 2.00 | 1.00 | 2.25 |
| Increased_verbosity.min | 1.00 | 2.00 | 1.00 | 2.00 | 1.00 | 1.00 |
| Increased_verbosity.max | 5.00 | 6.00 | 5.00 | 8.00 | 6.00 | 8.00 |
| Overlearned_phrases.mean | 1.61 | 3.25 | 1.64 | 2.92 | 1.88 | 2.39 |
| Overlearned_phrases.sd | 0.70 | 2.18 | 0.67 | 1.57 | 1.18 | 1.43 |
| Overlearned_phrases.median | 1.50 | 2.00 | 2.00 | 2.50 | 2.00 | 2.00 |
| Overlearned_phrases.IQR | 1.00 | 1.75 | 1.00 | 1.00 | 1.00 | 2.00 |
| Overlearned_phrases.min | 1.00 | 1.00 | 1.00 | 1.00 | 1.00 | 1.00 |
| Overlearned_phrases.max | 3.00 | 8.00 | 4.00 | 7.00 | 8.00 | 7.00 |
| Improper_use_of_pronouns.mean | 1.39 | 2.00 | 1.24 | 2.58 | 1.36 | 2.09 |
| Improper_use_of_pronouns.sd | 0.50 | 1.13 | 0.53 | 1.53 | 0.70 | 1.34 |
| Improper_use_of_pronouns.median | 1.00 | 2.00 | 1.00 | 2.00 | 1.00 | 2.00 |
| Improper_use_of_pronouns.IQR | 1.00 | 1.00 | 0.00 | 1.75 | 1.00 | 2.00 |
| Improper_use_of_pronouns.min | 1.00 | 1.00 | 1.00 | 1.00 | 1.00 | 1.00 |
| Improper_use_of_pronouns.max | 2.00 | 5.00 | 4.00 | 6.00 | 5.00 | 6.00 |
| Hesitation.mean | 4.72 | 6.08 | 4.18 | 6.73 | 4.47 | 5.91 |
| Hesitation.sd | 1.23 | 1.00 | 1.47 | 1.34 | 1.56 | 1.63 |
| Hesitation.median | 4.50 | 6.00 | 4.00 | 7.00 | 4.00 | 6.00 |
| Hesitation.IQR | 1.00 | 1.25 | 2.00 | 2.00 | 3.00 | 3.00 |
| Hesitation.min | 2.00 | 4.00 | 1.00 | 4.00 | 1.00 | 2.00 |
| Hesitation.max | 7.00 | 7.00 | 7.00 | 9.00 | 7.00 | 9.00 |
| Circumlocution.mean | 2.61 | 4.75 | 2.32 | 4.92 | 2.69 | 3.98 |
| Circumlocution.sd | 0.92 | 1.60 | 0.99 | 1.32 | 1.41 | 1.64 |
| Circumlocution.median | 2.50 | 4.50 | 2.00 | 5.00 | 2.00 | 4.00 |
| Circumlocution.IQR | 1.00 | 2.00 | 1.00 | 2.00 | 1.00 | 2.00 |
| Circumlocution.min | 1.00 | 2.00 | 1.00 | 2.00 | 1.00 | 1.00 |
| Circumlocution.max | 5.00 | 8.00 | 6.00 | 7.00 | 8.00 | 7.00 |
| Tip-of-the-tongue_phenomenon.mean | 2.17 | 3.67 | 1.88 | 4.69 | 2.15 | 3.66 |
| Tip-of-the-tongue_phenomenon.sd | 1.04 | 1.15 | 0.79 | 1.57 | 1.07 | 1.85 |
| Tip-of-the-tongue_phenomenon.median | 2.00 | 3.50 | 2.00 | 5.00 | 2.00 | 4.00 |
| Tip-of-the-tongue_phenomenon.IQR | 1.50 | 2.00 | 1.00 | 1.75 | 2.00 | 3.00 |
| Tip-of-the-tongue_phenomenon.min | 1.00 | 2.00 | 1.00 | 1.00 | 1.00 | 1.00 |
| Tip-of-the-tongue_phenomenon.max | 4.00 | 5.00 | 4.00 | 7.00 | 5.00 | 7.00 |
| Repetition.mean | 2.83 | 5.42 | 2.94 | 4.88 | 3.32 | 4.05 |
| Repetition.sd | 0.92 | 1.62 | 1.24 | 1.70 | 1.57 | 1.75 |
| Repetition.median | 3.00 | 6.00 | 3.00 | 4.00 | 3.00 | 4.00 |
| Repetition.IQR | 1.00 | 1.50 | 2.00 | 2.75 | 2.00 | 2.00 |
| Repetition.min | 1.00 | 2.00 | 1.00 | 2.00 | 1.00 | 1.00 |
| Repetition.max | 5.00 | 7.00 | 7.00 | 8.00 | 7.00 | 8.00 |
| Simplified_sentence_structure_and_grammar.mean | 2.72 | 5.17 | 2.92 | 5.35 | 3.27 | 4.27 |
| Simplified_sentence_structure_and_grammar.sd | 0.75 | 1.27 | 1.15 | 1.62 | 1.42 | 1.86 |
| Simplified_sentence_structure_and_grammar.median | 3.00 | 5.00 | 3.00 | 5.50 | 3.00 | 4.00 |
| Simplified_sentence_structure_and_grammar.IQR | 1.00 | 2.00 | 1.00 | 3.00 | 2.00 | 3.00 |
| Simplified_sentence_structure_and_grammar.min | 2.00 | 3.00 | 1.00 | 3.00 | 1.00 | 2.00 |
| Simplified_sentence_structure_and_grammar.max | 4.00 | 7.00 | 7.00 | 8.00 | 7.00 | 8.00 |
| Grammatical_errors.mean | 1.56 | 2.92 | 1.55 | 2.77 | 1.76 | 2.27 |
| Grammatical_errors.sd | 0.62 | 1.31 | 0.81 | 1.11 | 1.02 | 1.11 |
| Grammatical_errors.median | 1.50 | 2.50 | 1.00 | 2.50 | 2.00 | 2.00 |
| Grammatical_errors.IQR | 1.00 | 2.00 | 1.00 | 1.00 | 1.00 | 1.00 |
| Grammatical_errors.min | 1.00 | 1.00 | 1.00 | 1.00 | 1.00 | 1.00 |
| Grammatical_errors.max | 3.00 | 5.00 | 6.00 | 6.00 | 6.00 | 6.00 |
| Difficulty_with_complex_ideas.mean | 2.33 | 4.33 | 2.14 | 5.27 | 2.47 | 4.07 |
| Difficulty_with_complex_ideas.sd | 0.69 | 1.23 | 0.70 | 1.12 | 1.12 | 1.74 |
| Difficulty_with_complex_ideas.median | 2.00 | 4.50 | 2.00 | 5.00 | 2.00 | 4.00 |
| Difficulty_with_complex_ideas.IQR | 1.00 | 1.25 | 0.00 | 1.00 | 1.00 | 3.00 |
| Difficulty_with_complex_ideas.min | 1.00 | 2.00 | 1.00 | 4.00 | 1.00 | 1.00 |
| Difficulty_with_complex_ideas.max | 4.00 | 6.00 | 4.00 | 8.00 | 6.00 | 8.00 |
| Semantic_paraphasias.mean | 1.89 | 2.92 | 1.68 | 3.65 | 1.87 | 2.93 |
| Semantic_paraphasias.sd | 0.83 | 1.16 | 0.83 | 1.41 | 0.99 | 1.48 |
| Semantic_paraphasias.median | 2.00 | 3.00 | 2.00 | 3.00 | 2.00 | 3.00 |
| Semantic_paraphasias.IQR | 1.00 | 2.00 | 1.00 | 1.75 | 1.00 | 2.00 |
| Semantic_paraphasias.min | 1.00 | 1.00 | 1.00 | 1.00 | 1.00 | 1.00 |
| Semantic_paraphasias.max | 4.00 | 5.00 | 6.00 | 6.00 | 6.00 | 6.00 |
| Lack_of_coherence.mean | 2.17 | 4.33 | 1.89 | 4.69 | 2.27 | 3.66 |
| Lack_of_coherence.sd | 0.71 | 1.15 | 0.73 | 1.44 | 1.19 | 1.72 |
| Lack_of_coherence.median | 2.00 | 4.50 | 2.00 | 5.00 | 2.00 | 3.00 |
| Lack_of_coherence.IQR | 1.00 | 2.00 | 1.00 | 1.00 | 1.75 | 3.00 |
| Lack_of_coherence.min | 1.00 | 3.00 | 1.00 | 2.00 | 1.00 | 1.00 |
| Lack_of_coherence.max | 3.00 | 6.00 | 4.00 | 7.00 | 6.00 | 7.00 |
| Consistency_in_errors.mean | 1.44 | 3.50 | 1.29 | 3.58 | 1.63 | 2.70 |
| Consistency_in_errors.sd | 0.62 | 1.17 | 0.49 | 1.70 | 1.02 | 1.72 |
| Consistency_in_errors.median | 1.00 | 3.50 | 1.00 | 4.00 | 1.00 | 2.00 |
| Consistency_in_errors.IQR | 1.00 | 1.50 | 1.00 | 2.75 | 1.00 | 3.00 |
| Consistency_in_errors.min | 1.00 | 2.00 | 1.00 | 1.00 | 1.00 | 1.00 |
| Consistency_in_errors.max | 3.00 | 5.00 | 3.00 | 7.00 | 5.00 | 7.00 |
| Lack_of_semantic_fluency.mean | 2.56 | 4.67 | 2.08 | 5.04 | 2.47 | 4.02 |
| Lack_of_semantic_fluency.sd | 1.10 | 1.15 | 0.85 | 1.25 | 1.30 | 1.70 |
| Lack_of_semantic_fluency.median | 2.00 | 5.00 | 2.00 | 5.00 | 2.00 | 4.00 |
| Lack_of_semantic_fluency.IQR | 1.00 | 0.25 | 1.75 | 2.00 | 1.00 | 2.25 |
| Lack_of_semantic_fluency.min | 1.00 | 2.00 | 1.00 | 3.00 | 1.00 | 1.00 |
| Lack_of_semantic_fluency.max | 5.00 | 6.00 | 5.00 | 7.00 | 6.00 | 7.00 |
| Disorganized_narrative_structure.mean | 2.28 | 4.75 | 2.00 | 4.81 | 2.42 | 3.77 |
| Disorganized_narrative_structure.sd | 0.83 | 0.97 | 0.78 | 1.23 | 1.28 | 1.65 |
| Disorganized_narrative_structure.median | 2.00 | 5.00 | 2.00 | 5.00 | 2.00 | 4.00 |
| Disorganized_narrative_structure.IQR | 1.00 | 1.25 | 1.00 | 1.75 | 1.00 | 3.00 |
| Disorganized_narrative_structure.min | 1.00 | 3.00 | 1.00 | 2.00 | 1.00 | 1.00 |
| Disorganized_narrative_structure.max | 4.00 | 6.00 | 4.00 | 7.00 | 6.00 | 7.00 |
| Orientation_issues.mean | 1.22 | 2.25 | 1.11 | 2.92 | 1.28 | 2.23 |
| Orientation_issues.sd | 0.43 | 0.97 | 0.31 | 1.79 | 0.62 | 1.63 |
| Orientation_issues.median | 1.00 | 2.00 | 1.00 | 3.00 | 1.00 | 2.00 |
| Orientation_issues.IQR | 0.00 | 0.25 | 0.00 | 2.50 | 0.00 | 2.00 |
| Orientation_issues.min | 1.00 | 1.00 | 1.00 | 1.00 | 1.00 | 1.00 |
| Orientation_issues.max | 2.00 | 4.00 | 2.00 | 8.00 | 4.00 | 8.00 |
| Decline_in_conversational_engagement_and_responsiveness.mean | 1.72 | 3.50 | 1.76 | 3.77 | 2.03 | 2.93 |
| Decline_in_conversational_engagement_and_responsiveness.sd | 0.57 | 1.17 | 0.72 | 1.39 | 1.02 | 1.52 |
| Decline_in_conversational_engagement_and_responsiveness.median | 2.00 | 3.00 | 2.00 | 3.00 | 2.00 | 3.00 |
| Decline_in_conversational_engagement_and_responsiveness.IQR | 1.00 | 1.00 | 1.00 | 2.00 | 1.00 | 2.00 |
| Decline_in_conversational_engagement_and_responsiveness.min | 1.00 | 2.00 | 1.00 | 1.00 | 1.00 | 1.00 |
| Decline_in_conversational_engagement_and_responsiveness.max | 3.00 | 6.00 | 4.00 | 7.00 | 6.00 | 7.00 |
| Memory_issues.mean | 2.67 | 4.17 | 2.12 | 5.50 | 2.44 | 4.34 |
| Memory_issues.sd | 1.08 | 1.99 | 1.06 | 1.90 | 1.44 | 2.13 |
| Memory_issues.median | 2.50 | 4.00 | 2.00 | 6.00 | 2.00 | 4.00 |
| Memory_issues.IQR | 1.00 | 4.00 | 2.00 | 2.50 | 2.00 | 4.00 |
| Memory_issues.min | 1.00 | 2.00 | 1.00 | 2.00 | 1.00 | 1.00 |
| Memory_issues.max | 5.00 | 7.00 | 5.00 | 10.00 | 7.00 | 10.00 |
| Lack_of_abstract_thinking_and_synthesis.mean | 2.06 | 4.25 | 1.88 | 4.73 | 2.24 | 3.64 |
| Lack_of_abstract_thinking_and_synthesis.sd | 0.73 | 1.22 | 0.71 | 1.54 | 1.18 | 1.83 |
| Lack_of_abstract_thinking_and_synthesis.median | 2.00 | 4.00 | 2.00 | 5.00 | 2.00 | 3.00 |
| Lack_of_abstract_thinking_and_synthesis.IQR | 0.00 | 2.25 | 1.00 | 2.00 | 2.00 | 3.00 |
| Lack_of_abstract_thinking_and_synthesis.min | 1.00 | 3.00 | 1.00 | 1.00 | 1.00 | 1.00 |
| Lack_of_abstract_thinking_and_synthesis.max | 4.00 | 6.00 | 3.00 | 7.00 | 6.00 | 7.00 |
| Abnormal_emotions.mean | 1.06 | 1.92 | 1.14 | 2.00 | 1.26 | 1.61 |
| Abnormal_emotions.sd | 0.24 | 1.00 | 0.35 | 1.10 | 0.57 | 0.97 |
| Abnormal_emotions.median | 1.00 | 2.00 | 1.00 | 2.00 | 1.00 | 1.00 |
| Abnormal_emotions.IQR | 0.00 | 1.25 | 0.00 | 1.00 | 0.00 | 1.00 |
| Abnormal_emotions.min | 1.00 | 1.00 | 1.00 | 1.00 | 1.00 | 1.00 |
| Abnormal_emotions.max | 2.00 | 4.00 | 2.00 | 6.00 | 4.00 | 6.00 |

**Statistical analysis 2: normal distribution and Homogeneity of variance test, Independent samples t-test for the two groups, Mann-Whitney U test for the two groups**

| Variable | Shapiro Wilk p value | Levene p value (4groups) | Levene p value (2groups) | t test p value | Wilcox test p value |
| --- | --- | --- | --- | --- | --- |
| Limited_vocabulary | 2.05E-08 | 0.001709 | 0.072775 | 0.063193 | 0.084555 |
| Increased_verbosity | 2.94E-09 | 0.011126 | 0.250229 | 0.040403 | 0.046519 |
| Overlearned_phrases | 3.28E-14 | 0.0026 | 0.200604 | 0.052202 | 0.021069 |
| Improper_use_of_pronouns | 4.45E-16 | 2.47E-05 | 0.000492 | 0.001387 | 0.000148 |
| Hesitation | 0.000625 | 0.456978 | 0.898278 | 8.07E-06 | 1.97E-05 |
| Circumlocution | 3.31E-08 | 0.042575 | 0.070296 | 3.65E-05 | 1.09E-05 |
| Tip-of-the-tongue_phenomenon | 5.87E-09 | 0.008003 | 1.61E-05 | 6.68E-06 | 9.99E-06 |
| Repetition | 5.56E-07 | 0.044777 | 0.39898 | 0.025511 | 0.021743 |
| Simplified_sentence_structure_and_grammar | 1.07E-08 | 0.001791 | 0.015923 | 0.002728 | 0.002846 |
| Grammatical_errors | 1.63E-12 | 0.061091 | 0.981456 | 0.012865 | 0.001877 |
| Difficulty_with_complex_ideas | 1.29E-09 | 0.016002 | 0.000157 | 8.56E-07 | 3.57E-07 |
| Semantic_paraphasias | 5.56E-11 | 0.017012 | 0.00147 | 7.28E-05 | 2.05E-05 |
| Lack_of_coherence | 2.97E-09 | 0.000503 | 0.00095 | 1.15E-05 | 4.79E-06 |
| Consistency_in_errors | 1.83E-13 | 4.27E-09 | 0.000979 | 0.000347 | 0.000152 |
| Lack_of_semantic_fluency | 6.6E-08 | 0.185818 | 0.013909 | 1.61E-06 | 1.36E-06 |
| Disorganized_narrative_structure | 6.99E-08 | 0.038433 | 0.025199 | 1.33E-05 | 7.45E-06 |
| Orientation_issues | 8.23E-17 | 4.44E-11 | 9.05E-08 | 0.000526 | 7.7E-05 |
| Decline_in_conversational_engagement_and_responsiveness | 2.95E-10 | 0.00597 | 0.002418 | 0.000743 | 0.000419 |
| Memory_issues | 1.16E-08 | 0.001493 | 0.000854 | 1.56E-06 | 5.33E-07 |
| Lack_of_abstract_thinking_and_synthesis | 8.35E-10 | 7.41E-05 | 2.33E-05 | 2.53E-05 | 3.44E-05 |
| Abnormal_emotions | 1.34E-17 | 1.42E-05 | 0.011444 | 0.029043 | 0.007973 |

**Statistical analysis 3 and Violin plots: one-way ANOVA for the four groups (Tukey's Honestly Significant Difference test as a post hoc test), and Kruskal-Wallis H-test for the four groups (Dunn's test was applied as a post hoc test)**

| Variable | Comparison | ANOVA p value | Tukey p value | Kruskal Wallis p value | Dunn p value |
| --- | --- | --- | --- | --- | --- |
| Limited_vocabulary | FP-FN | 5.43E-12 | 7.38E-07 | 9.55E-09 | 1.41E-05 |
| Limited_vocabulary | TN-FN | 5.43E-12 | 0.893038 | 9.55E-09 | 0.462826 |
| Limited_vocabulary | TP-FN | 5.43E-12 | 4.40E-06 | 9.55E-09 | 5.77E-06 |
| Limited_vocabulary | TN-FP | 5.43E-12 | 8.89E-08 | 9.55E-09 | 4.43E-05 |
| Limited_vocabulary | TP-FP | 5.43E-12 | 0.491525 | 9.55E-09 | 0.29404 |
| Limited_vocabulary | TP-TN | 5.43E-12 | 7.83E-08 | 9.55E-09 | 5.01E-06 |
| Increased_verbosity | FP-FN | 2.89E-08 | 0.00067 | 2.4E-06 | 0.000986 |
| Increased_verbosity | TN-FN | 2.89E-08 | 0.999674 | 2.4E-06 | 0.826812 |
| Increased_verbosity | TP-FN | 2.89E-08 | 0.001162 | 2.4E-06 | 4.17E-05 |
| Increased_verbosity | TN-FP | 2.89E-08 | 2.6E-05 | 2.4E-06 | 0.004439 |
| Increased_verbosity | TP-FP | 2.89E-08 | 0.810829 | 2.4E-06 | 0.308544 |
| Increased_verbosity | TP-TN | 2.89E-08 | 5.35E-06 | 2.4E-06 | 5.85E-05 |
| Overlearned_phrases | FP-FN | 1.32E-07 | 0.000983 | 4.39E-06 | 0.005053 |
| Overlearned_phrases | TN-FN | 1.32E-07 | 0.999788 | 4.39E-06 | 0.898141 |
| Overlearned_phrases | TP-FN | 1.32E-07 | 0.001425 | 4.39E-06 | 0.001278 |
| Overlearned_phrases | TN-FP | 1.32E-07 | 8.12E-05 | 4.39E-06 | 0.000562 |
| Overlearned_phrases | TP-FP | 1.32E-07 | 0.841885 | 4.39E-06 | 0.970642 |
| Overlearned_phrases | TP-TN | 1.32E-07 | 1.8E-05 | 4.39E-06 | 9.82E-06 |
| Improper_use_of_pronouns | FP-FN | 2.72E-08 | 0.264526 | 4.23E-07 | 0.098426 |
| Improper_use_of_pronouns | TN-FN | 2.72E-08 | 0.92722 | 4.23E-07 | 0.268252 |
| Improper_use_of_pronouns | TP-FN | 2.72E-08 | 0.000187 | 4.23E-07 | 0.003725 |
| Improper_use_of_pronouns | TN-FP | 2.72E-08 | 0.039846 | 4.23E-07 | 0.001939 |
| Improper_use_of_pronouns | TP-FP | 2.72E-08 | 0.257754 | 4.23E-07 | 0.337871 |
| Improper_use_of_pronouns | TP-TN | 2.72E-08 | 1.68E-08 | 4.23E-07 | 7.63E-08 |
| Hesitation | FP-FN | 2.29E-12 | 0.042874 | 6.62E-10 | 0.022594 |
| Hesitation | TN-FN | 2.29E-12 | 0.450687 | 6.62E-10 | 0.23827 |
| Hesitation | TP-FN | 2.29E-12 | 2.98E-05 | 6.62E-10 | 0.00021 |
| Hesitation | TN-FP | 2.29E-12 | 0.000127 | 6.62E-10 | 0.000214 |
| Hesitation | TP-FP | 2.29E-12 | 0.530553 | 6.62E-10 | 0.413858 |
| Hesitation | TP-TN | 2.29E-12 | 4.79E-12 | 6.62E-10 | 3.95E-10 |
| Circumlocution | FP-FN | 9.71E-19 | 8.51E-06 | 6.1E-13 | 0.001332 |
| Circumlocution | TN-FN | 9.71E-19 | 0.764025 | 6.1E-13 | 0.298389 |
| Circumlocution | TP-FN | 9.71E-19 | 5.15E-09 | 6.1E-13 | 2.7E-06 |
| Circumlocution | TN-FP | 9.71E-19 | 2.06E-09 | 6.1E-13 | 1.67E-05 |
| Circumlocution | TP-FP | 9.71E-19 | 0.971612 | 6.1E-13 | 0.72239 |
| Circumlocution | TP-TN | 9.71E-19 | 8.22E-15 | 6.1E-13 | 5.39E-12 |
| Tip-of-the-tongue_phenomenon | FP-FN | 3.24E-20 | 0.001537 | 4.06E-12 | 0.004859 |
| Tip-of-the-tongue_phenomenon | TN-FN | 3.24E-20 | 0.744236 | 4.06E-12 | 0.440409 |
| Tip-of-the-tongue_phenomenon | TP-FN | 3.24E-20 | 3.07E-11 | 4.06E-12 | 6.39E-05 |
| Tip-of-the-tongue_phenomenon | TN-FP | 3.24E-20 | 3.08E-06 | 4.06E-12 | 5.82E-06 |
| Tip-of-the-tongue_phenomenon | TP-FP | 3.24E-20 | 0.035276 | 4.06E-12 | 0.329547 |
| Tip-of-the-tongue_phenomenon | TP-TN | 3.24E-20 | 0 | 4.06E-12 | 5.64E-12 |
| Repetition | FP-FN | 1.8E-11 | 6.95E-06 | 3.08E-08 | 0.000203 |
| Repetition | TN-FN | 1.8E-11 | 0.991032 | 3.08E-08 | 0.843724 |
| Repetition | TP-FN | 1.8E-11 | 1.49E-05 | 3.08E-08 | 2.19E-05 |
| Repetition | TN-FP | 1.8E-11 | 2.85E-07 | 3.08E-08 | 0.000138 |
| Repetition | TP-FP | 1.8E-11 | 0.673485 | 3.08E-08 | 0.536145 |
| Repetition | TP-TN | 1.8E-11 | 4.85E-08 | 3.08E-08 | 1.43E-06 |
| Simplified_sentence_structure_and_grammar | FP-FN | 1.07E-15 | 3.05E-06 | 1.73E-11 | 4.78E-05 |
| Simplified_sentence_structure_and_grammar | TN-FN | 1.07E-15 | 0.926962 | 1.73E-11 | 0.670178 |
| Simplified_sentence_structure_and_grammar | TP-FN | 1.07E-15 | 1.41E-09 | 1.73E-11 | 7.89E-06 |
| Simplified_sentence_structure_and_grammar | TN-FP | 1.07E-15 | 3.56E-07 | 1.73E-11 | 8.5E-07 |
| Simplified_sentence_structure_and_grammar | TP-FP | 1.07E-15 | 0.97553 | 1.73E-11 | 0.986796 |
| Simplified_sentence_structure_and_grammar | TP-TN | 1.07E-15 | 4.79E-13 | 1.73E-11 | 1.63E-09 |
| Grammatical_errors | FP-FN | 3.37E-09 | 0.000633 | 8.9E-09 | 0.001736 |
| Grammatical_errors | TN-FN | 3.37E-09 | 0.999974 | 8.9E-09 | 0.769719 |
| Grammatical_errors | TP-FN | 3.37E-09 | 0.000179 | 8.9E-09 | 7.26E-05 |
| Grammatical_errors | TN-FP | 3.37E-09 | 2.93E-05 | 8.9E-09 | 0.000111 |
| Grammatical_errors | TP-FP | 3.37E-09 | 0.966984 | 8.9E-09 | 0.958666 |
| Grammatical_errors | TP-TN | 3.37E-09 | 3.55E-07 | 8.9E-09 | 4.88E-08 |
| Difficulty_with_complex_ideas | FP-FN | 2.17E-31 | 4.44E-08 | 7.05E-17 | 0.000547 |
| Difficulty_with_complex_ideas | TN-FN | 2.17E-31 | 0.825398 | 7.05E-17 | 0.459448 |
| Difficulty_with_complex_ideas | TP-FN | 2.17E-31 | 0 | 7.05E-17 | 2.22E-06 |
| Difficulty_with_complex_ideas | TN-FP | 2.17E-31 | 2.96E-12 | 7.05E-17 | 4.36E-08 |
| Difficulty_with_complex_ideas | TP-FP | 2.17E-31 | 0.012272 | 7.05E-17 | 0.262988 |
| Difficulty_with_complex_ideas | TP-TN | 2.17E-31 | 0 | 7.05E-17 | 5.47E-16 |
| Semantic_paraphasias | FP-FN | 5.07E-13 | 0.036766 | 4.06E-10 | 0.021829 |
| Semantic_paraphasias | TN-FN | 5.07E-13 | 0.868117 | 4.06E-10 | 0.369032 |
| Semantic_paraphasias | TP-FN | 5.07E-13 | 5.7E-07 | 4.06E-10 | 0.000493 |
| Semantic_paraphasias | TN-FP | 5.07E-13 | 0.000954 | 4.06E-10 | 5.21E-05 |
| Semantic_paraphasias | TP-FP | 5.07E-13 | 0.163396 | 4.06E-10 | 0.268824 |
| Semantic_paraphasias | TP-TN | 5.07E-13 | 6.53E-13 | 4.06E-10 | 1.67E-10 |
| Lack_of_coherence | FP-FN | 4.17E-25 | 1.05E-07 | 5.15E-15 | 0.000254 |
| Lack_of_coherence | TN-FN | 4.17E-25 | 0.710031 | 5.15E-15 | 0.290075 |
| Lack_of_coherence | TP-FN | 4.17E-25 | 2.89E-13 | 5.15E-15 | 1.6E-07 |
| Lack_of_coherence | TN-FP | 4.17E-25 | 3.61E-12 | 5.15E-15 | 4.11E-06 |
| Lack_of_coherence | TP-FP | 4.17E-25 | 0.708177 | 5.15E-15 | 0.88801 |
| Lack_of_coherence | TP-TN | 4.17E-25 | 0 | 5.15E-15 | 2.59E-13 |
| Consistency_in_errors | FP-FN | 4.64E-20 | 4.66E-07 | 2.07E-13 | 5.71E-05 |
| Consistency_in_errors | TN-FN | 4.64E-20 | 0.928176 | 2.07E-13 | 0.471051 |
| Consistency_in_errors | TP-FN | 4.64E-20 | 3.2E-10 | 2.07E-13 | 7.05E-08 |
| Consistency_in_errors | TN-FP | 4.64E-20 | 1.94E-10 | 2.07E-13 | 1.53E-05 |
| Consistency_in_errors | TP-FP | 4.64E-20 | 0.995746 | 2.07E-13 | 0.618 |
| Consistency_in_errors | TP-TN | 4.64E-20 | 7.22E-15 | 2.07E-13 | 5.62E-11 |
| Lack_of_semantic_fluency | FP-FN | 5.09E-25 | 8.42E-07 | 7.67E-15 | 0.000915 |
| Lack_of_semantic_fluency | TN-FN | 5.09E-25 | 0.286092 | 7.67E-15 | 0.210409 |
| Lack_of_semantic_fluency | TP-FN | 5.09E-25 | 5.57E-12 | 7.67E-15 | 5.78E-07 |
| Lack_of_semantic_fluency | TN-FP | 5.09E-25 | 2.44E-12 | 7.67E-15 | 6.08E-06 |
| Lack_of_semantic_fluency | TP-FP | 5.09E-25 | 0.718379 | 7.67E-15 | 0.664401 |
| Lack_of_semantic_fluency | TP-TN | 5.09E-25 | 0 | 7.67E-15 | 1.1E-13 |
| Disorganized_narrative_structure | FP-FN | 1.05E-27 | 3.47E-10 | 5.91E-16 | 5.71E-05 |
| Disorganized_narrative_structure | TN-FN | 1.05E-27 | 0.668492 | 5.91E-16 | 0.359106 |
| Disorganized_narrative_structure | TP-FN | 1.05E-27 | 4.43E-14 | 5.91E-16 | 2.76E-08 |
| Disorganized_narrative_structure | TN-FP | 1.05E-27 | 1.43E-14 | 5.91E-16 | 1.59E-06 |
| Disorganized_narrative_structure | TP-FP | 1.05E-27 | 0.99793 | 5.91E-16 | 0.935991 |
| Disorganized_narrative_structure | TP-TN | 1.05E-27 | 0 | 5.91E-16 | 1.27E-13 |
| Orientation_issues | FP-FN | 1.67E-13 | 0.016941 | 6.91E-12 | 0.000792 |
| Orientation_issues | TN-FN | 1.67E-13 | 0.964278 | 6.91E-12 | 0.439353 |
| Orientation_issues | TP-FN | 1.67E-13 | 1.09E-07 | 6.91E-12 | 3.48E-06 |
| Orientation_issues | TN-FP | 1.67E-13 | 0.000707 | 6.91E-12 | 2.45E-05 |
| Orientation_issues | TP-FP | 1.67E-13 | 0.158896 | 6.91E-12 | 0.901719 |
| Orientation_issues | TP-TN | 1.67E-13 | 3.27E-13 | 6.91E-12 | 9.48E-11 |
| Decline_in_conversational_engagement_and_responsiveness | FP-FN | 4.33E-17 | 7.85E-06 | 1.31E-12 | 9.18E-05 |
| Decline_in_conversational_engagement_and_responsiveness | TN-FN | 4.33E-17 | 0.998972 | 1.31E-12 | 0.962519 |
| Decline_in_conversational_engagement_and_responsiveness | TP-FN | 4.33E-17 | 5.1E-10 | 1.31E-12 | 4.12E-06 |
| Decline_in_conversational_engagement_and_responsiveness | TN-FP | 4.33E-17 | 1.82E-07 | 1.31E-12 | 6.69E-07 |
| Decline_in_conversational_engagement_and_responsiveness | TP-FP | 4.33E-17 | 0.842846 | 1.31E-12 | 0.849353 |
| Decline_in_conversational_engagement_and_responsiveness | TP-TN | 4.33E-17 | 1.77E-14 | 1.31E-12 | 6.68E-11 |
| Memory_issues | FP-FN | 7.57E-18 | 0.022738 | 3.55E-11 | 0.077295 |
| Memory_issues | TN-FN | 7.57E-18 | 0.4538 | 3.55E-11 | 0.120634 |
| Memory_issues | TP-FN | 7.57E-18 | 5.49E-09 | 3.55E-11 | 0.000642 |
| Memory_issues | TN-FP | 7.57E-18 | 4.22E-05 | 3.55E-11 | 0.000117 |
| Memory_issues | TP-FP | 7.57E-18 | 0.034054 | 3.55E-11 | 0.134181 |
| Memory_issues | TP-TN | 7.57E-18 | 3.77E-15 | 3.55E-11 | 5.84E-12 |
| Lack_of_abstract_thinking_and_synthesis | FP-FN | 2.24E-24 | 2.07E-07 | 3.34E-14 | 0.000124 |
| Lack_of_abstract_thinking_and_synthesis | TN-FN | 2.24E-24 | 0.90943 | 3.34E-14 | 0.569888 |
| Lack_of_abstract_thinking_and_synthesis | TP-FN | 2.24E-24 | 1.18E-13 | 3.34E-14 | 4.68E-07 |
| Lack_of_abstract_thinking_and_synthesis | TN-FP | 2.24E-24 | 5.25E-11 | 3.34E-14 | 1.42E-06 |
| Lack_of_abstract_thinking_and_synthesis | TP-FP | 2.24E-24 | 0.513435 | 3.34E-14 | 0.890096 |
| Lack_of_abstract_thinking_and_synthesis | TP-TN | 2.24E-24 | 0 | 3.34E-14 | 1.95E-12 |
| Abnormal_emotions | FP-FN | 1.87E-08 | 0.002946 | 1.18E-08 | 0.0009 |
| Abnormal_emotions | TN-FN | 1.87E-08 | 0.965796 | 1.18E-08 | 0.532032 |
| Abnormal_emotions | TP-FN | 1.87E-08 | 3.41E-05 | 1.18E-08 | 0.000642 |
| Abnormal_emotions | TN-FP | 1.87E-08 | 0.001156 | 1.18E-08 | 4.41E-06 |
| Abnormal_emotions | TP-FP | 1.87E-08 | 0.982871 | 1.18E-08 | 0.625274 |
| Abnormal_emotions | TP-TN | 1.87E-08 | 4.24E-07 | 1.18E-08 | 8.23E-08 |


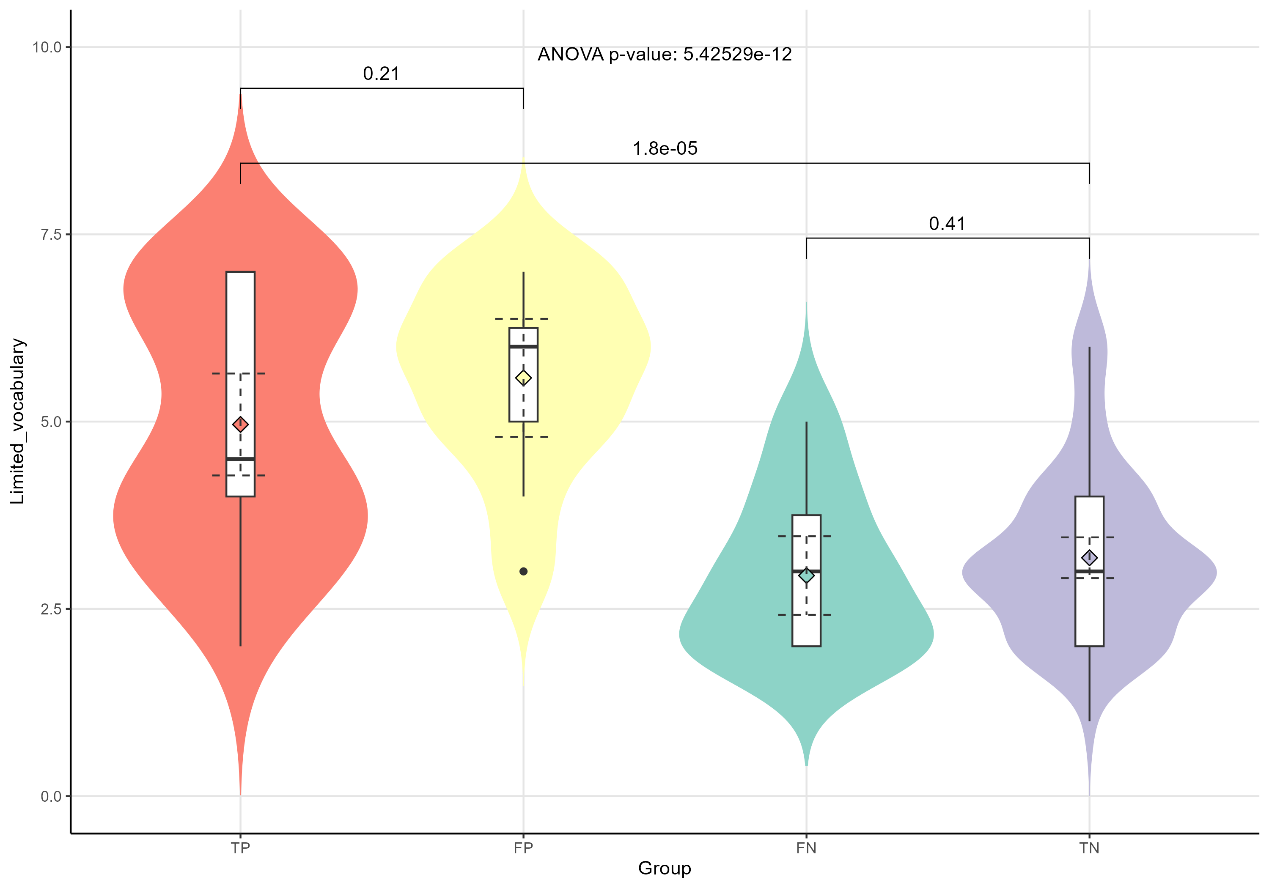


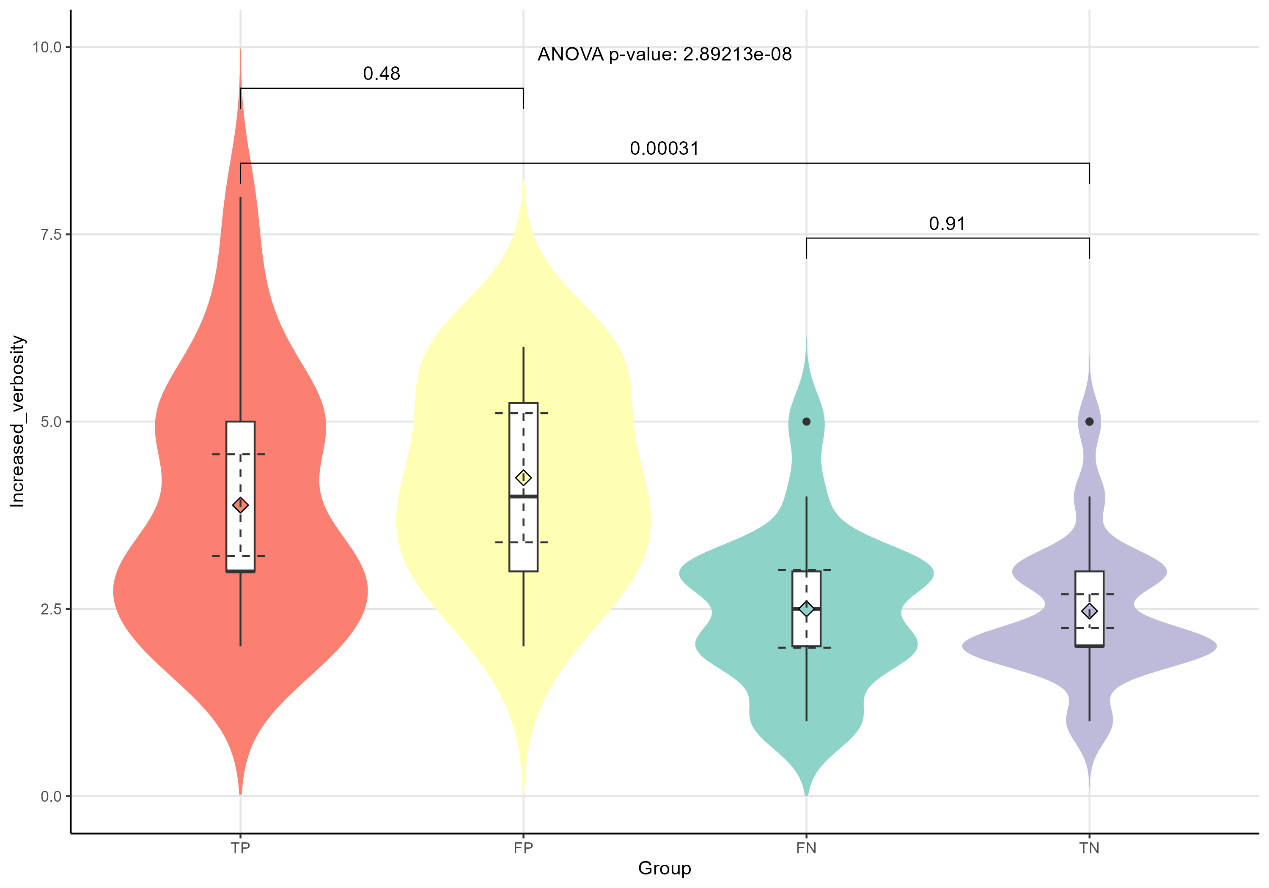


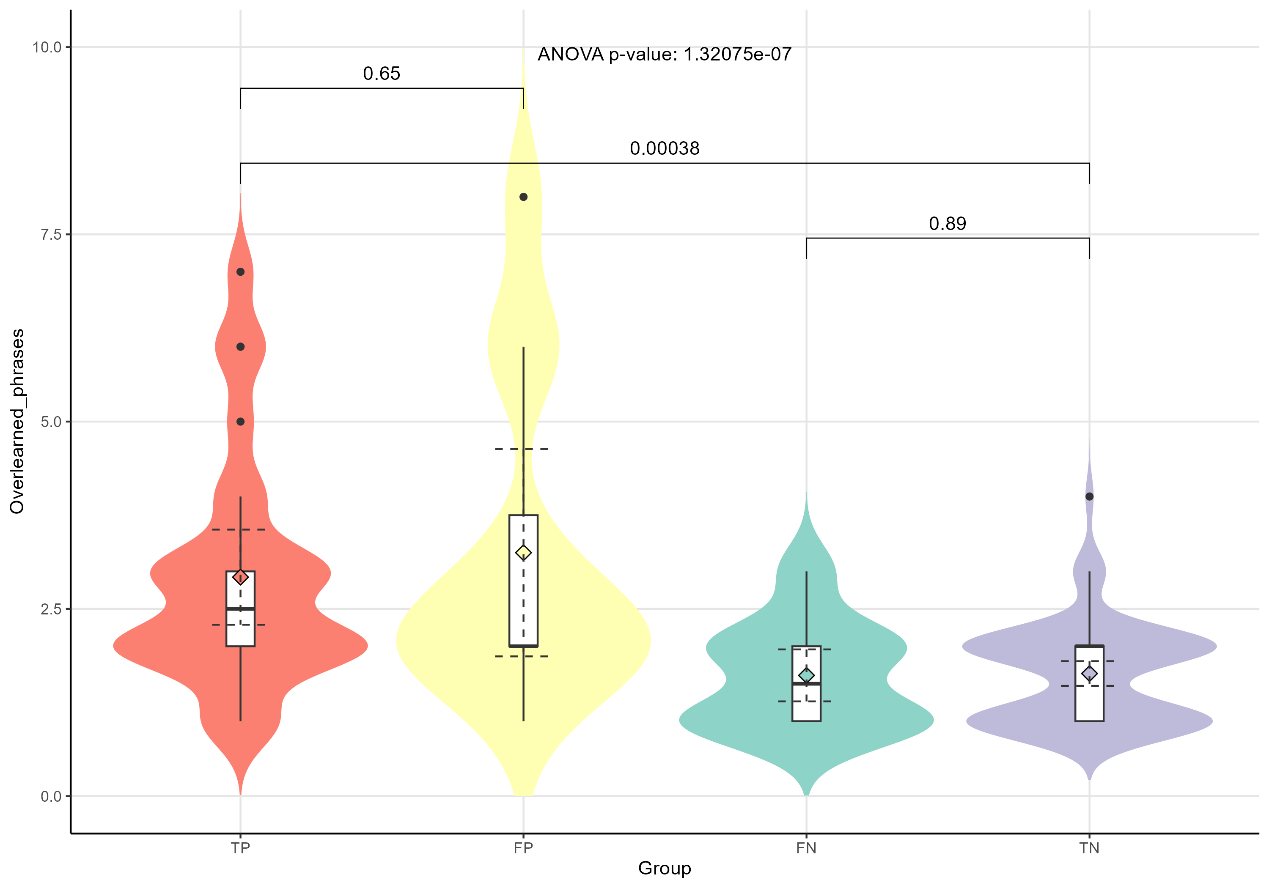


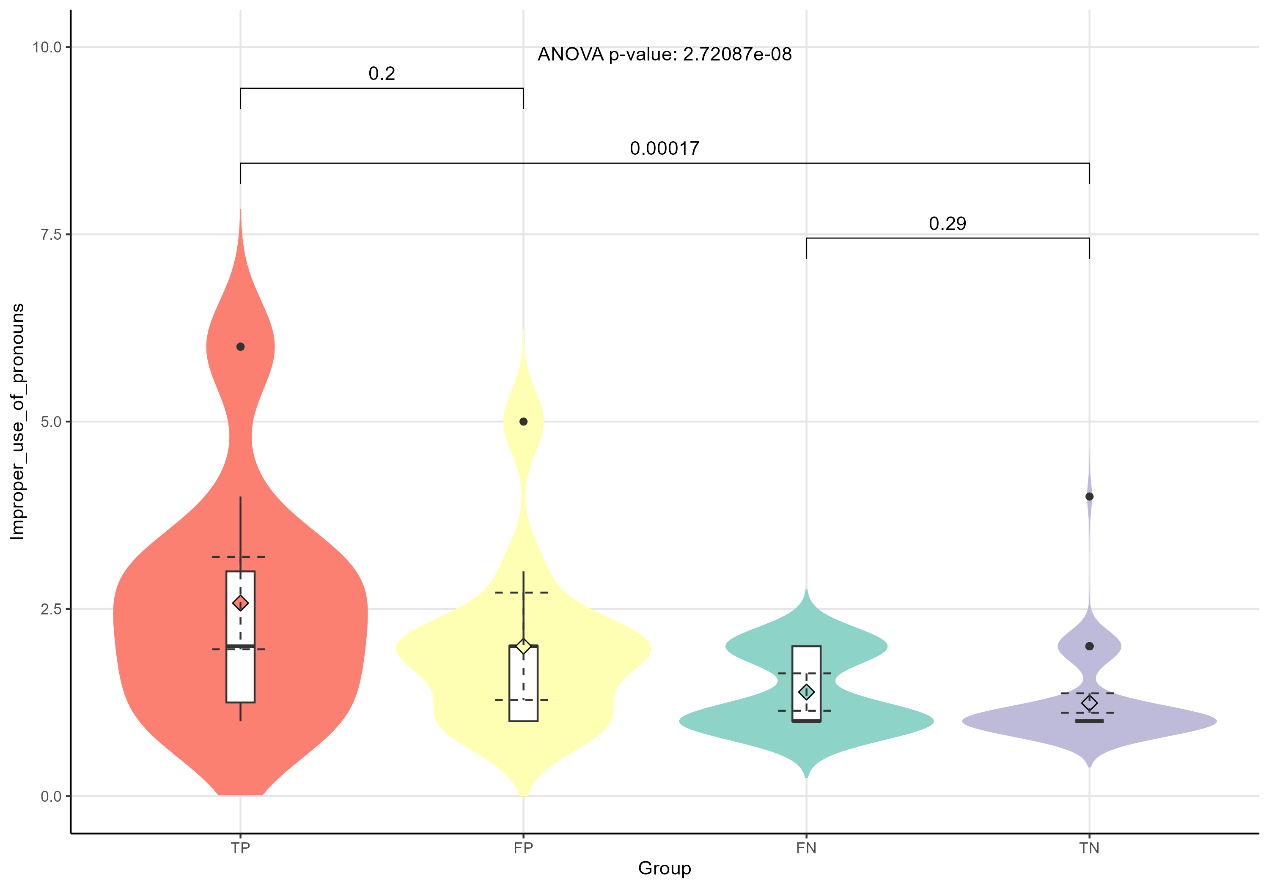


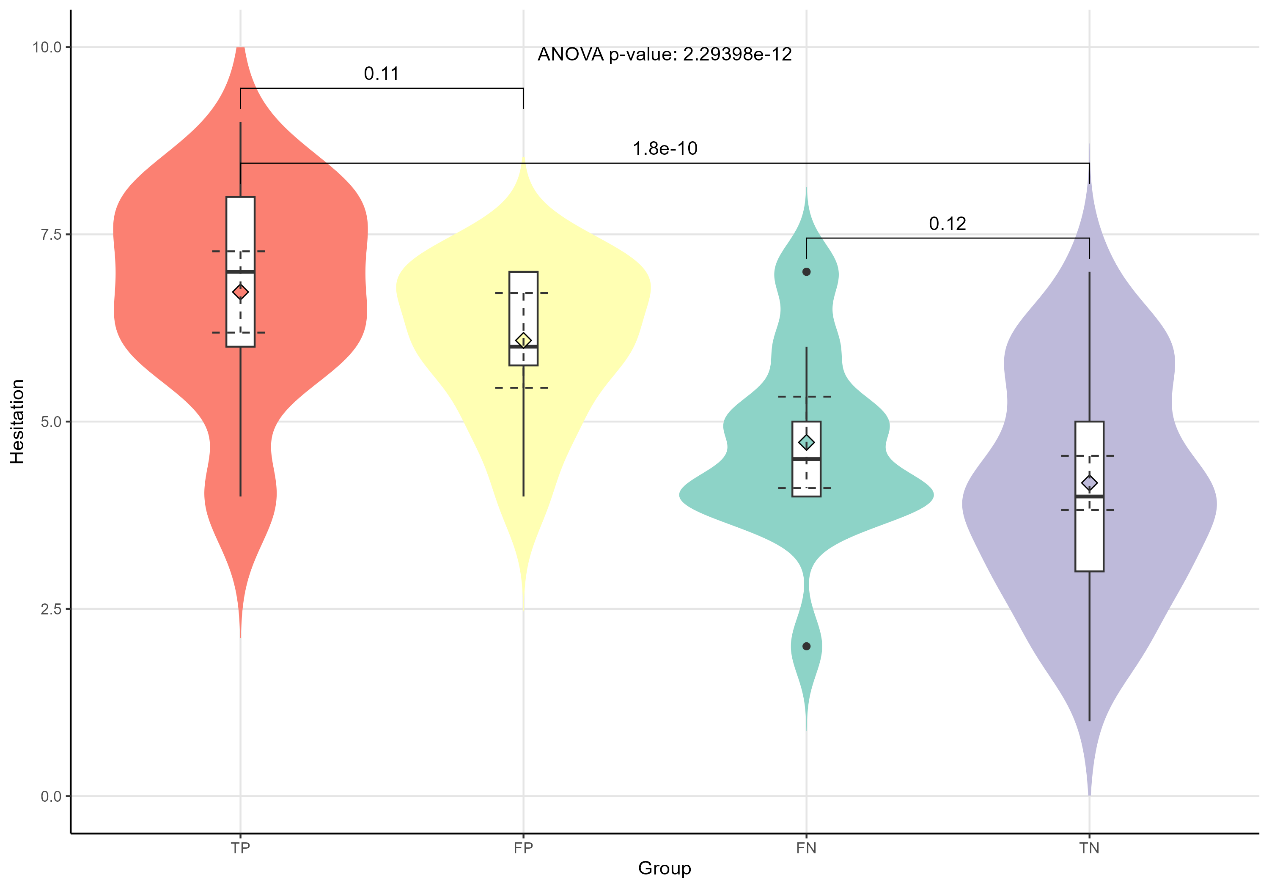


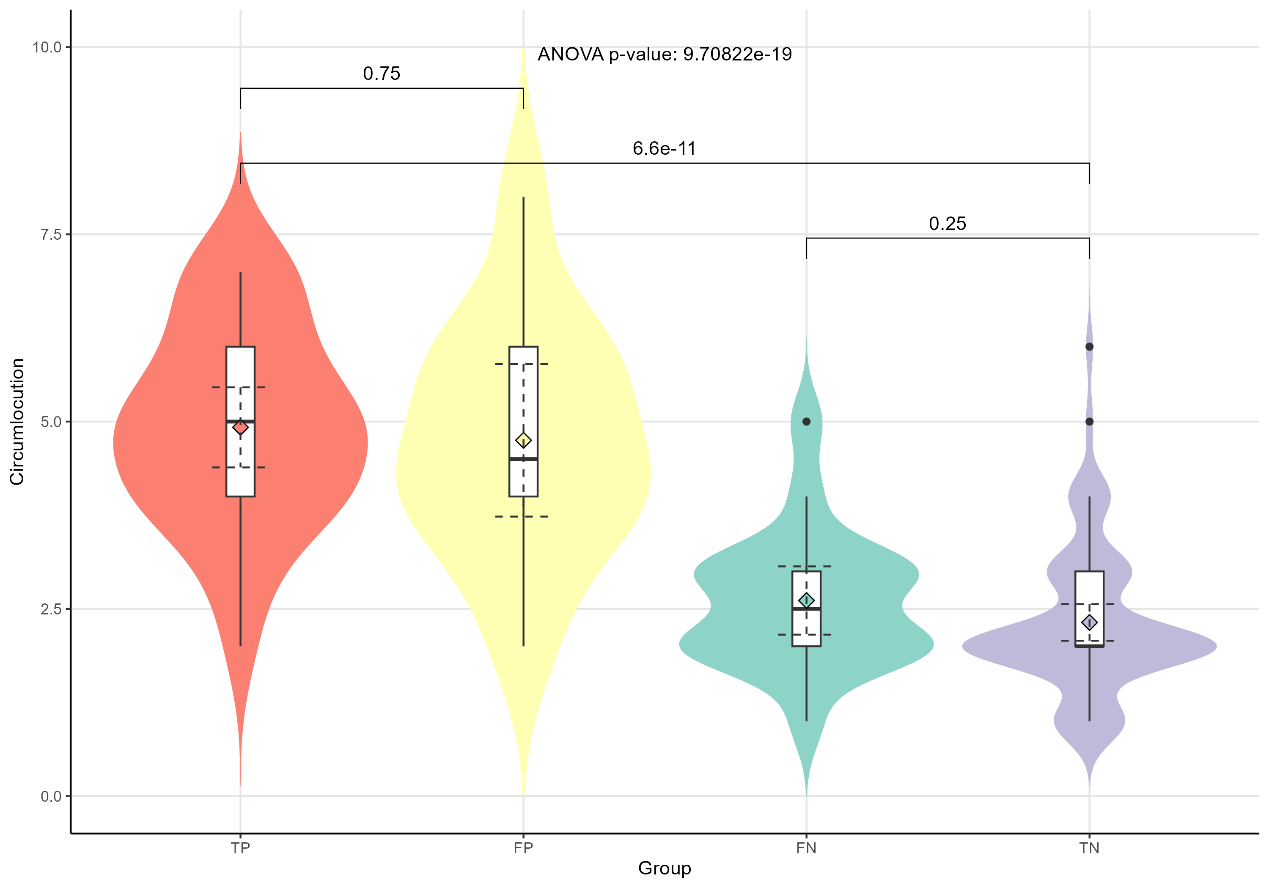


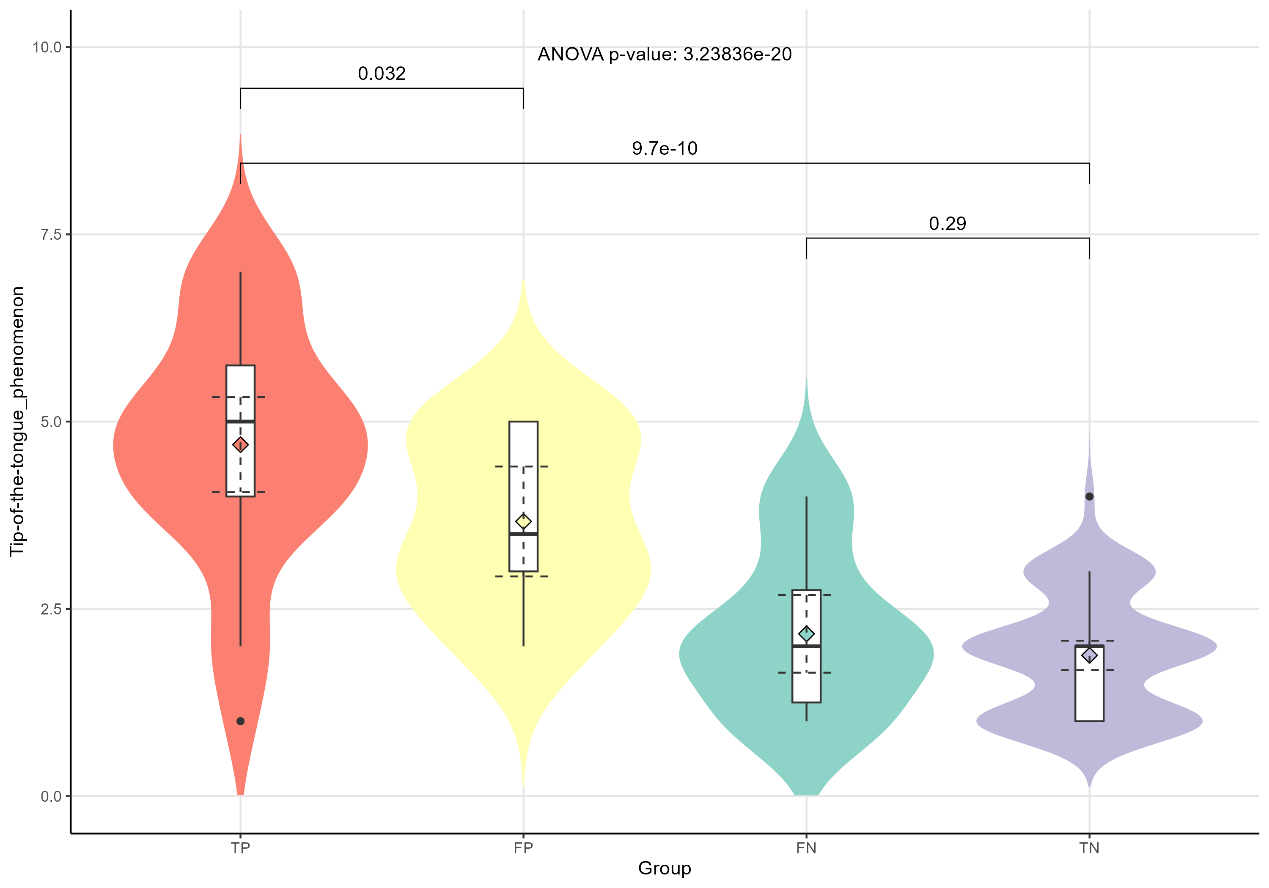


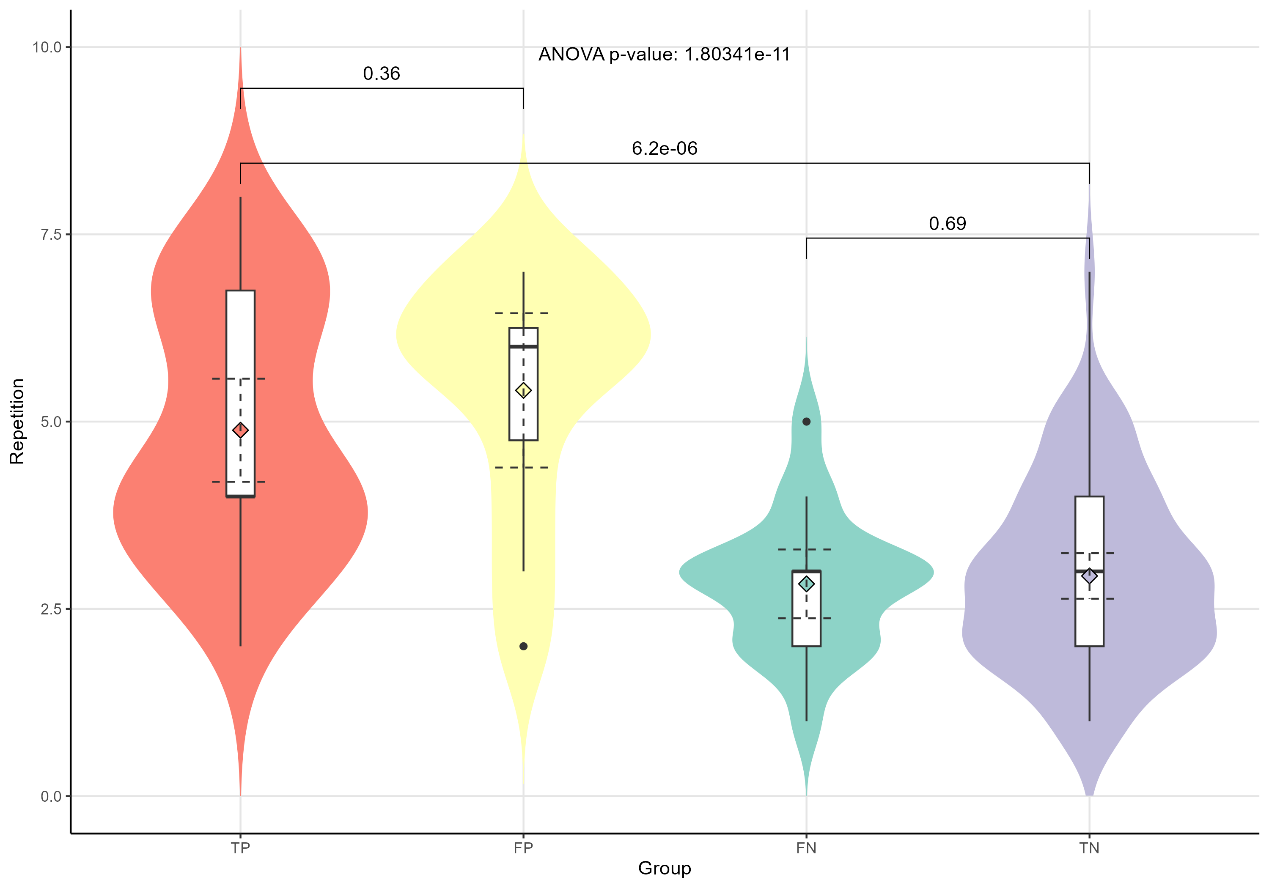


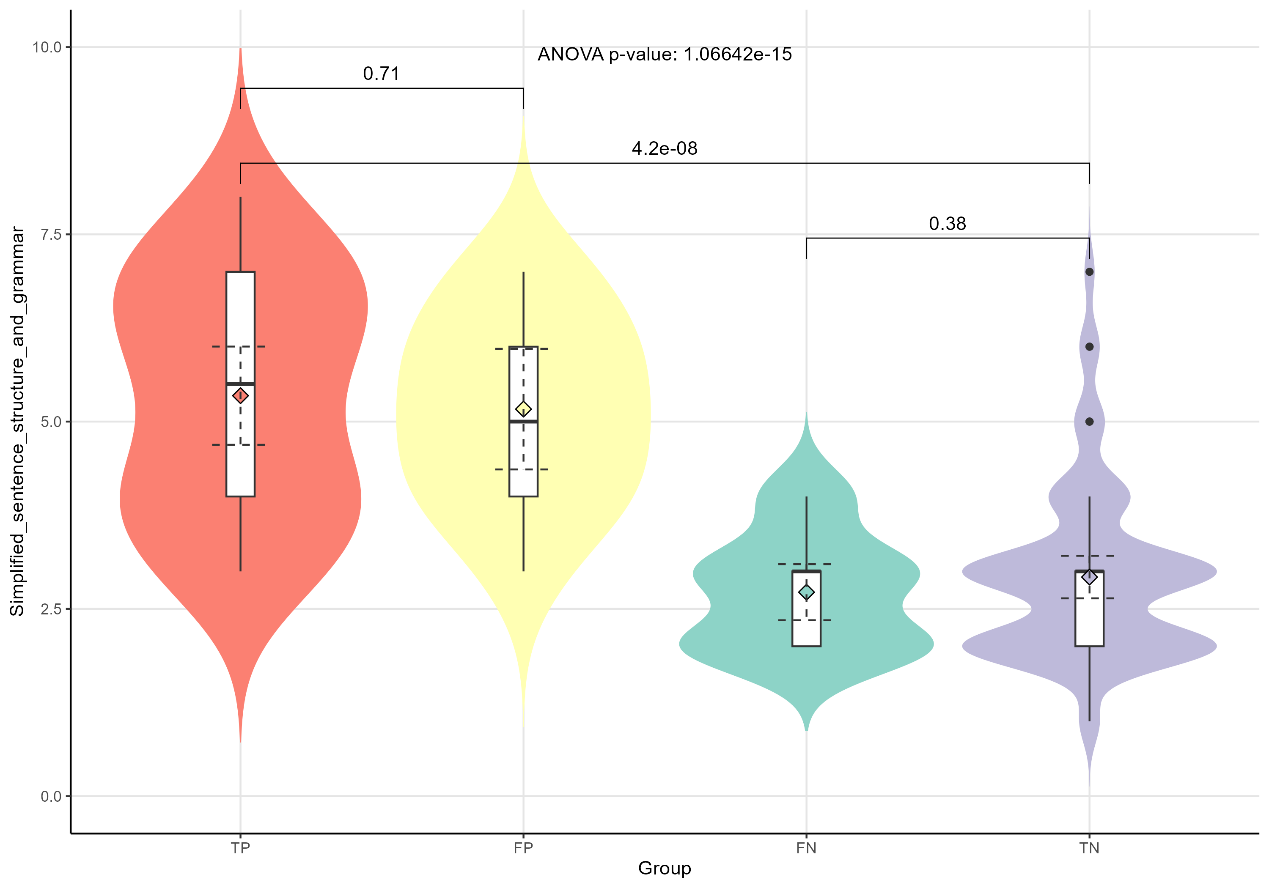


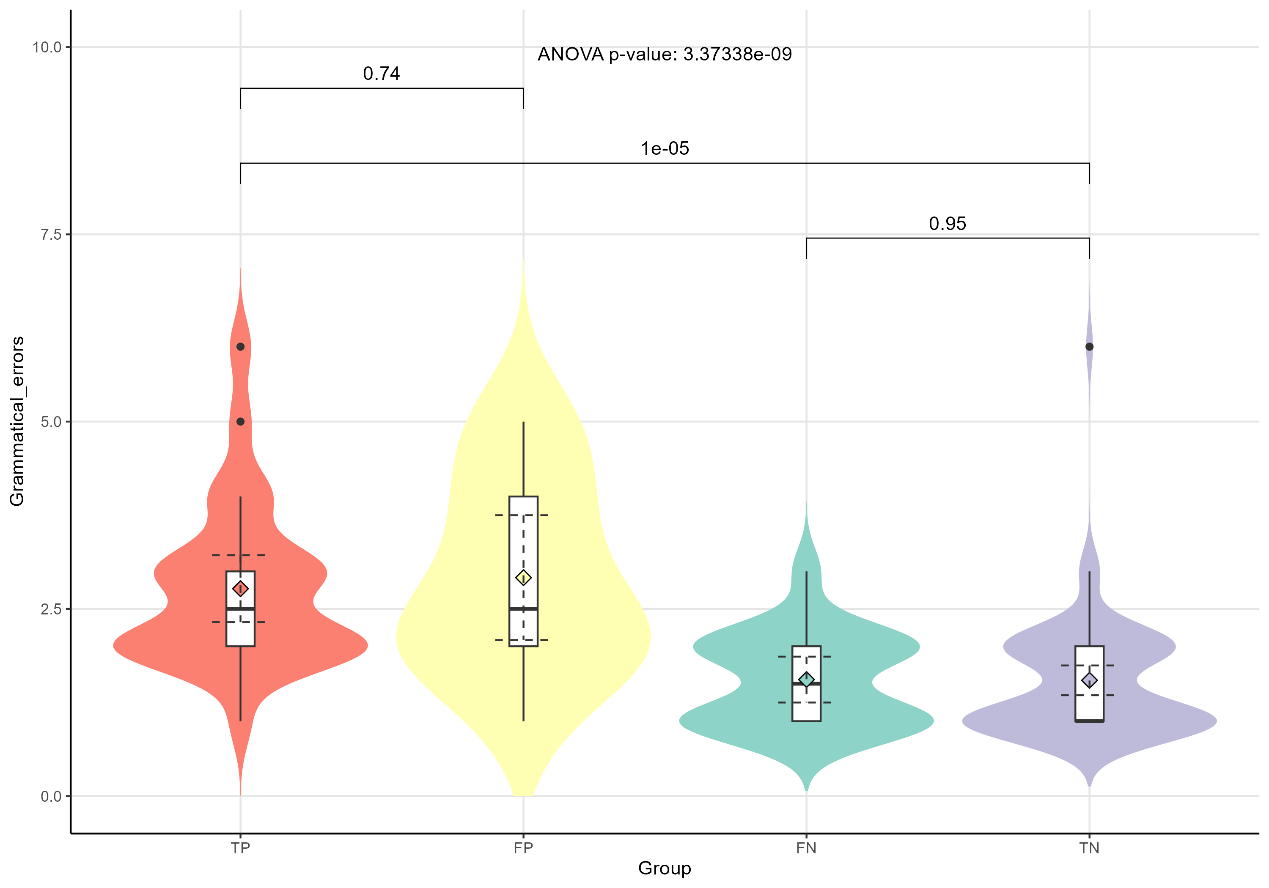


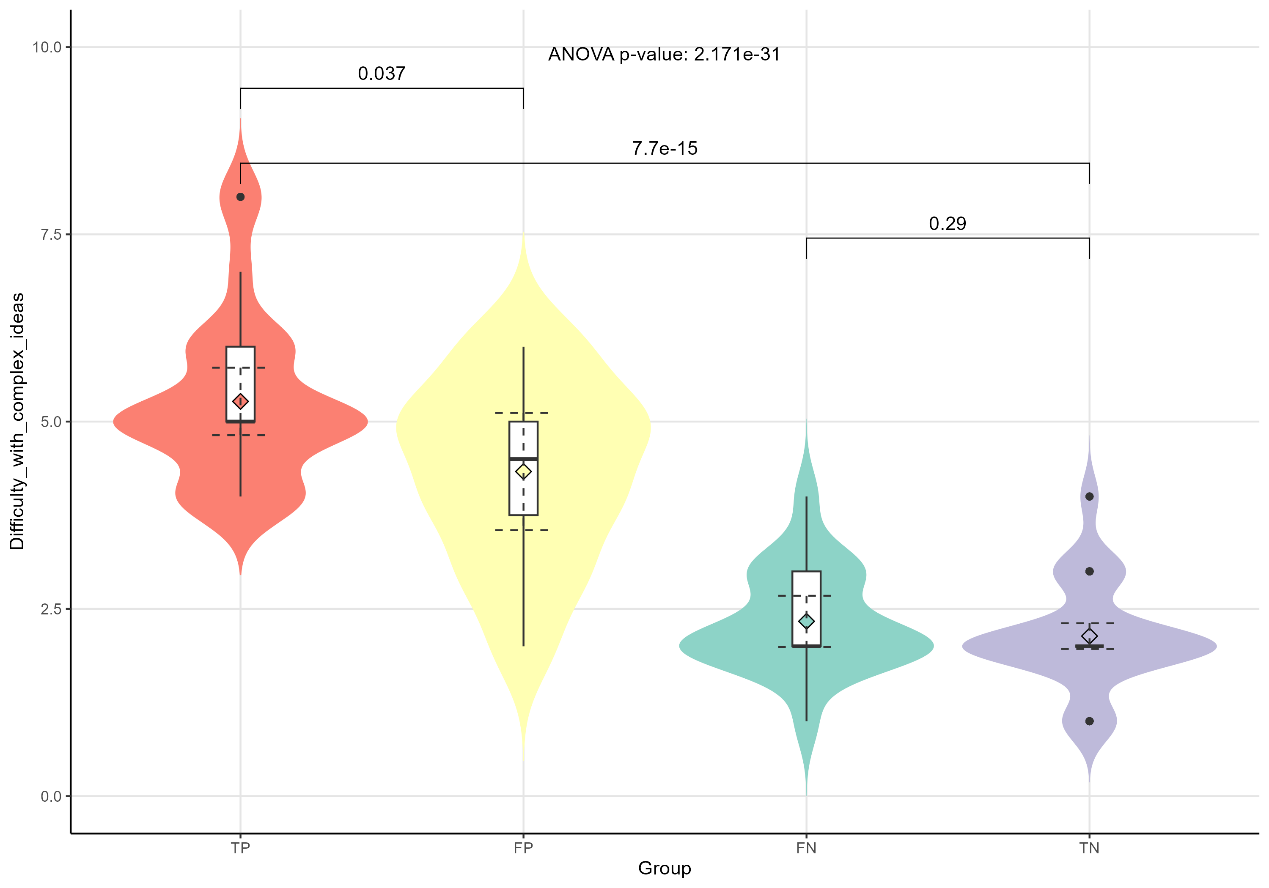


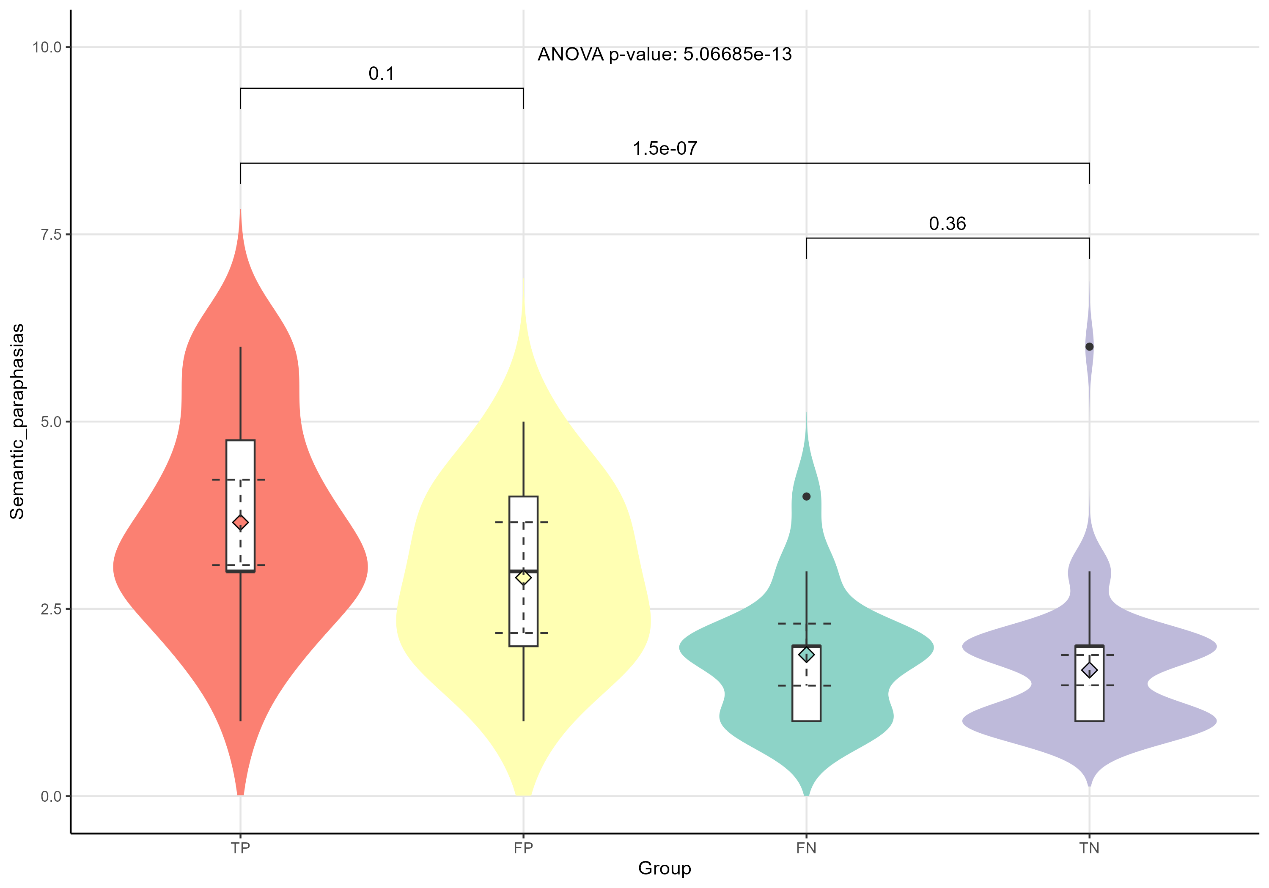


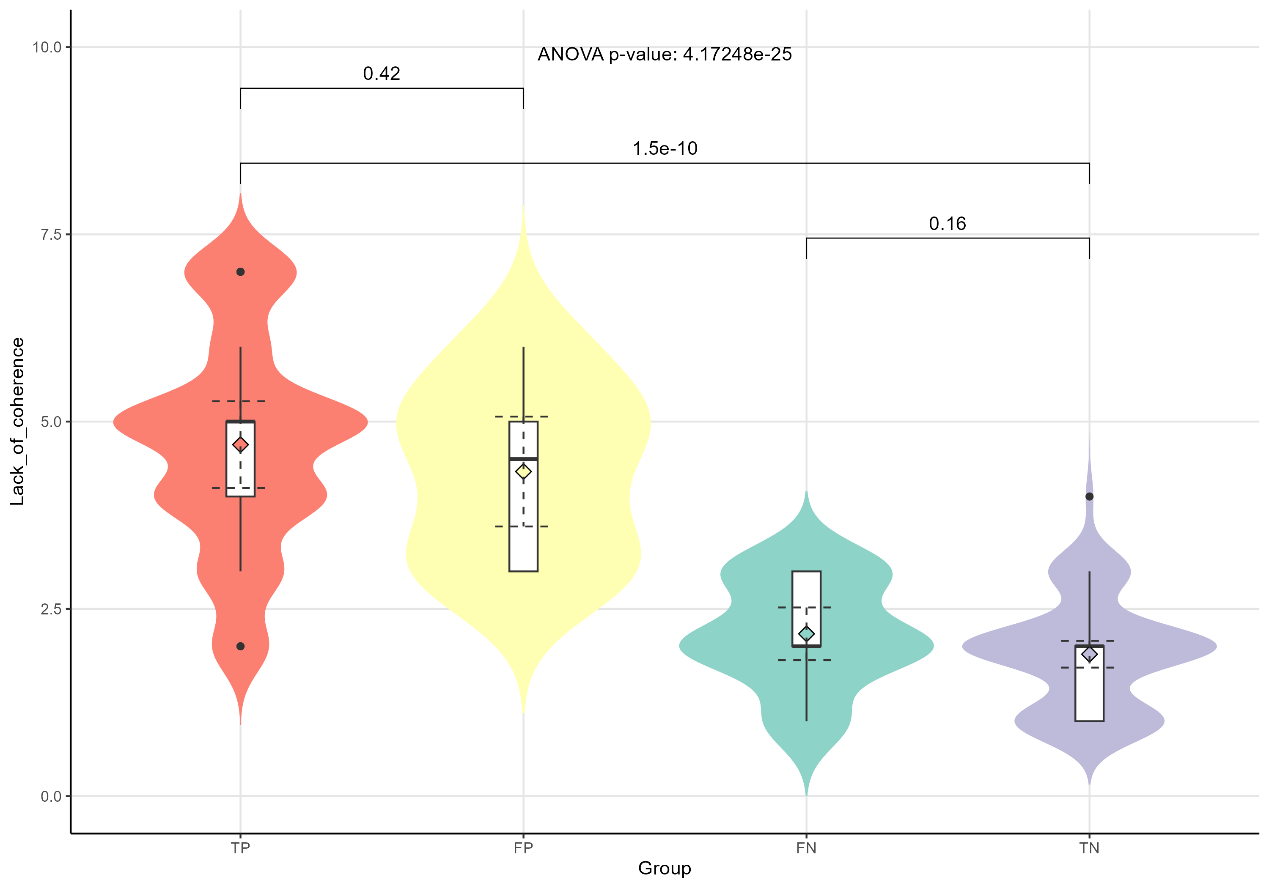


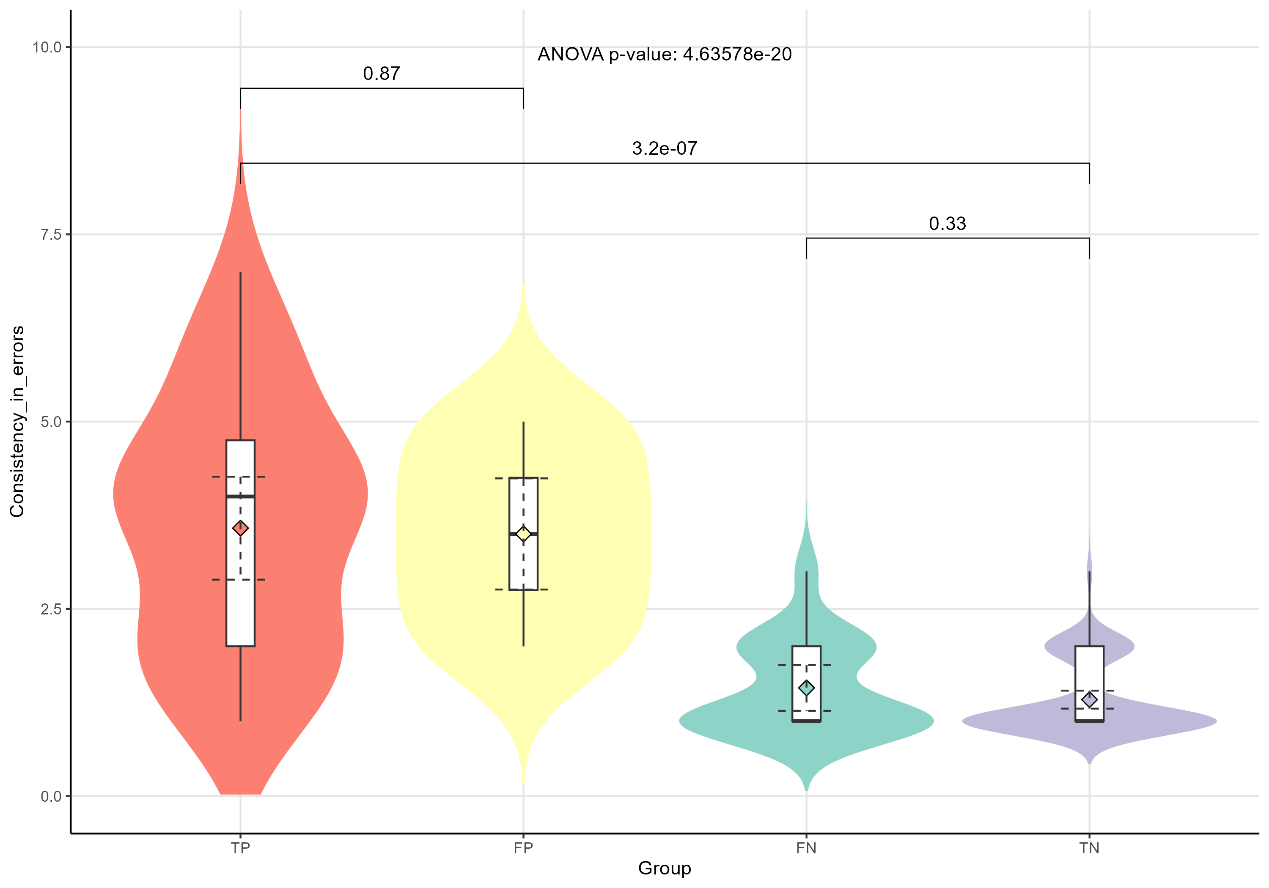


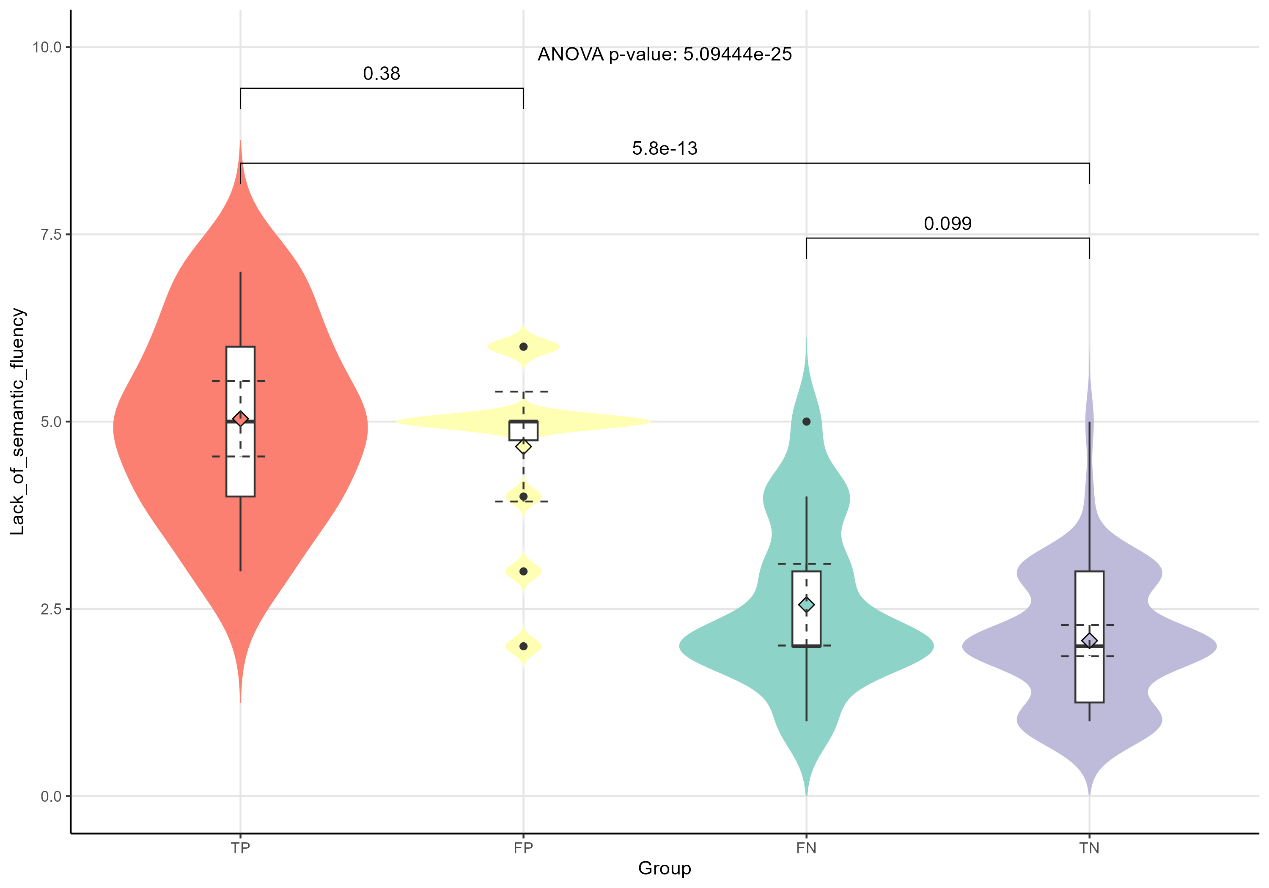


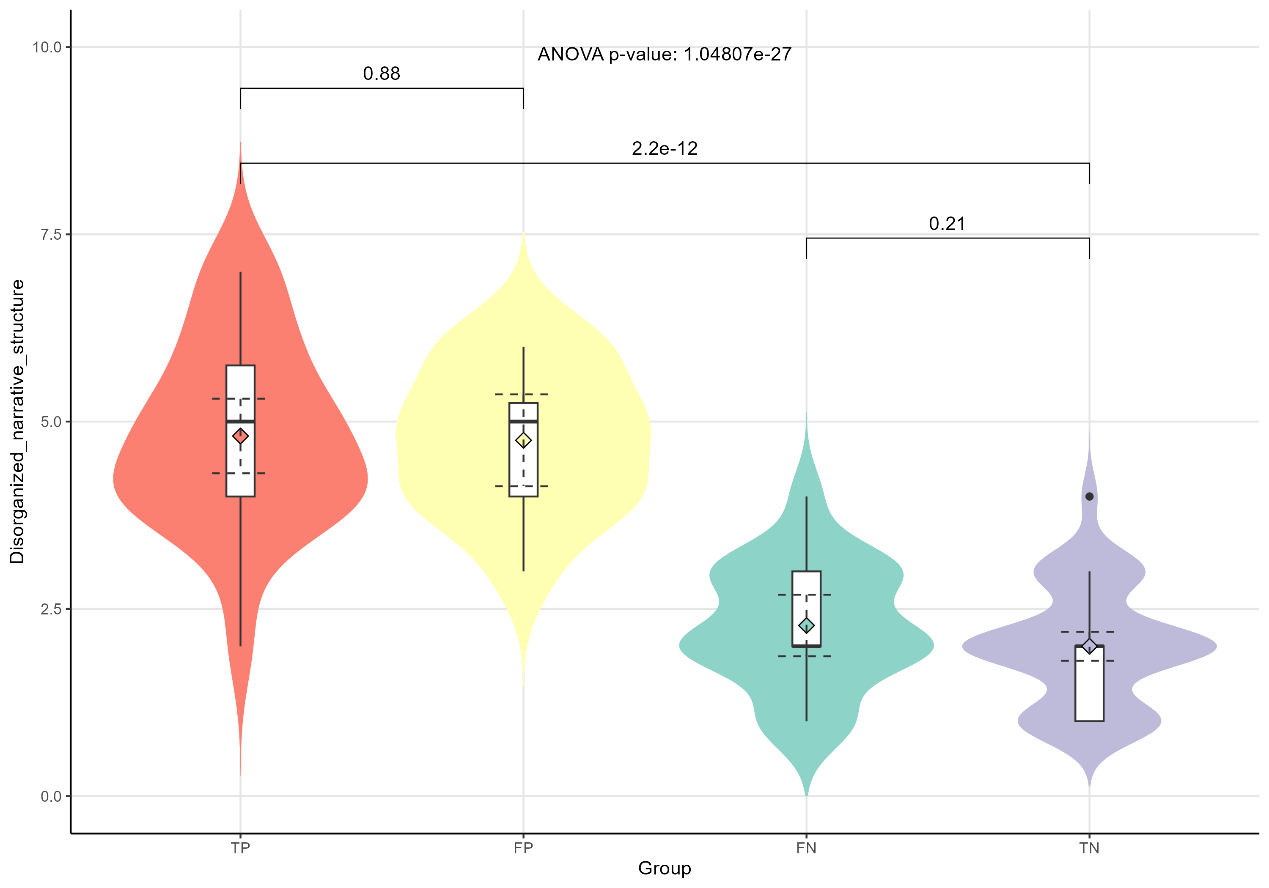


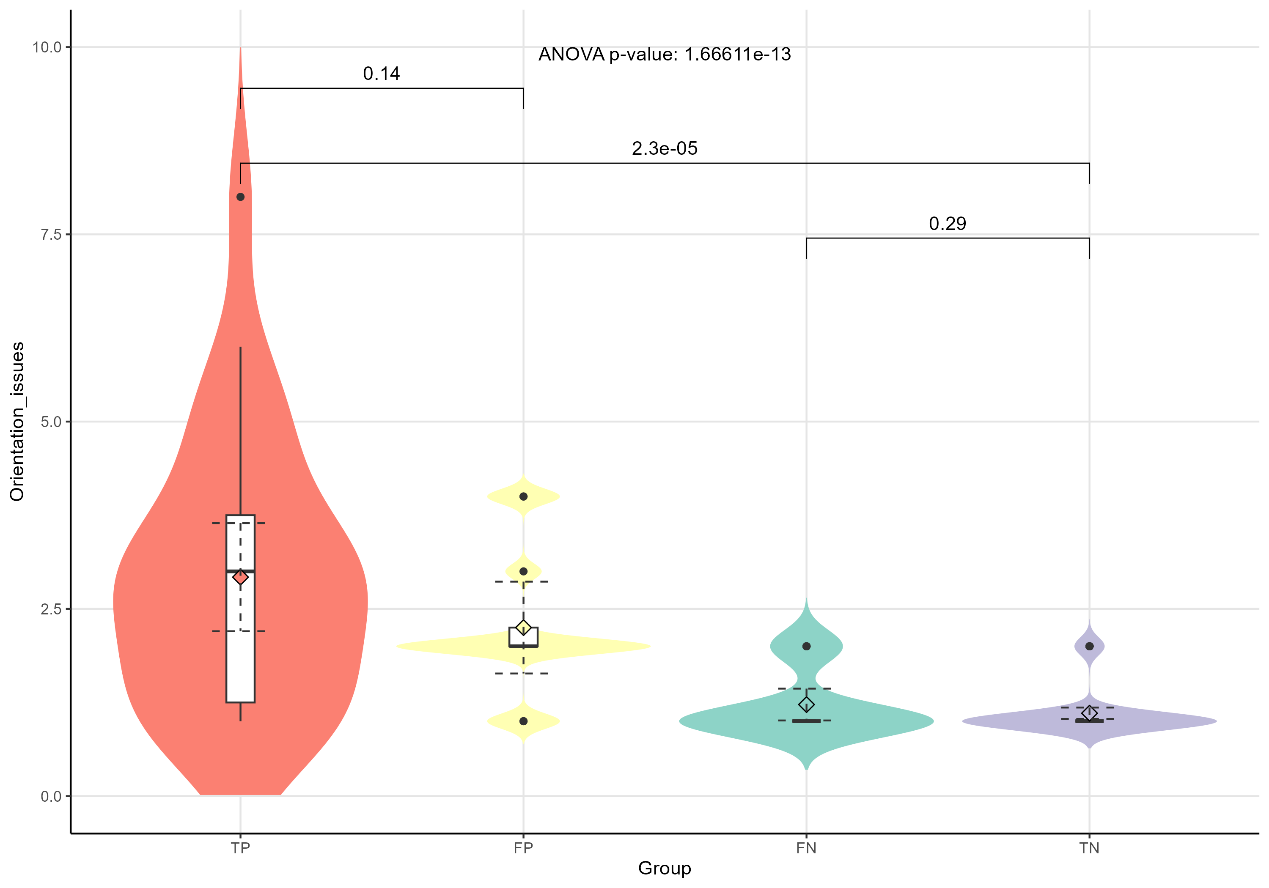


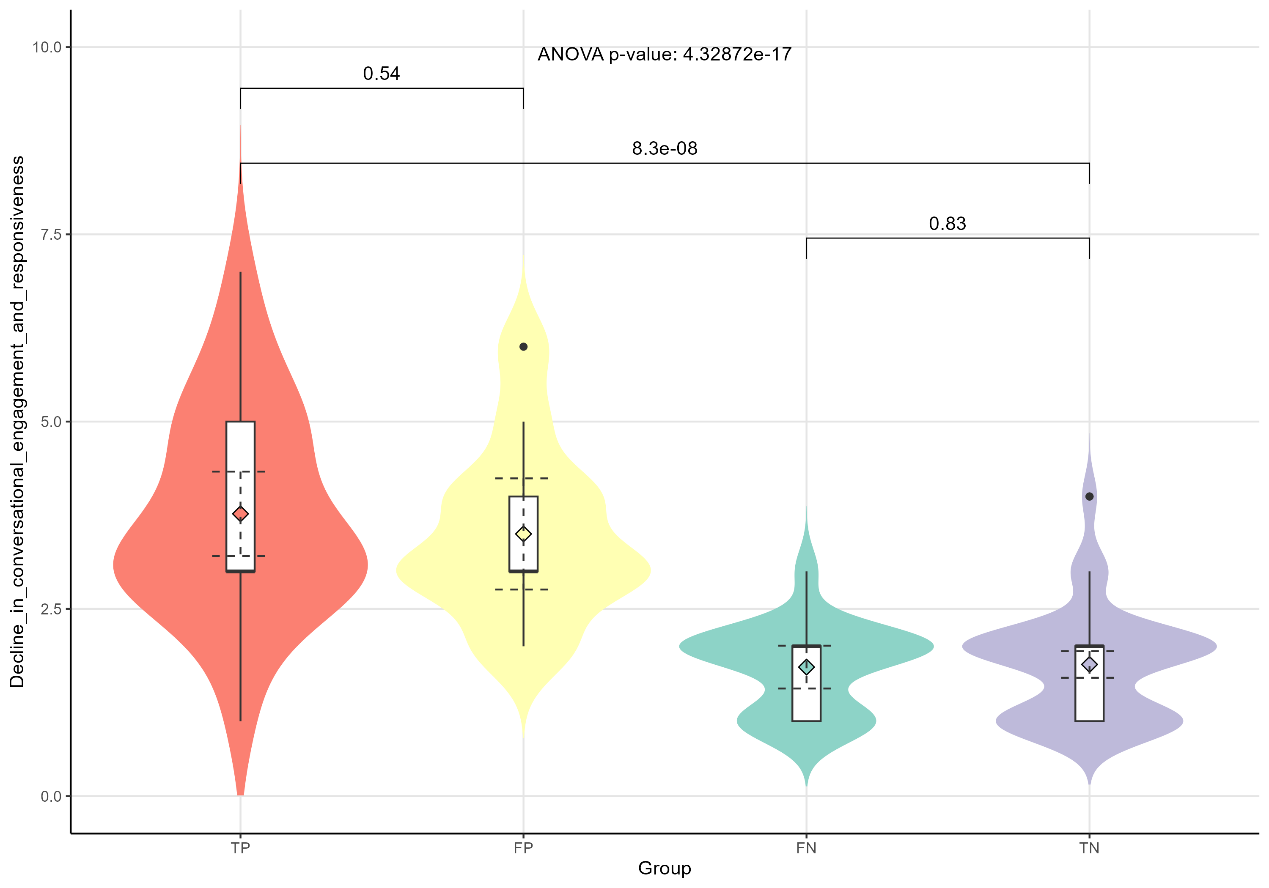


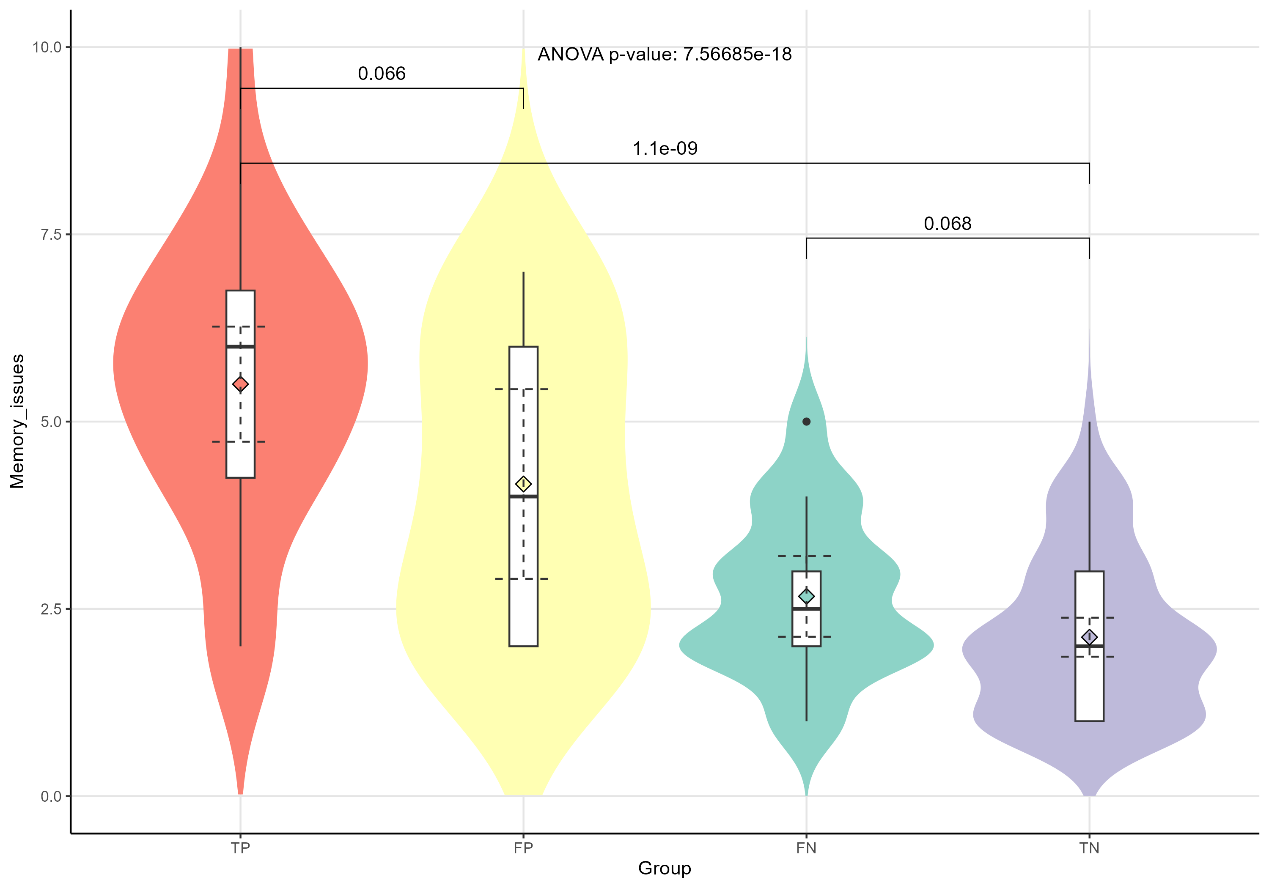


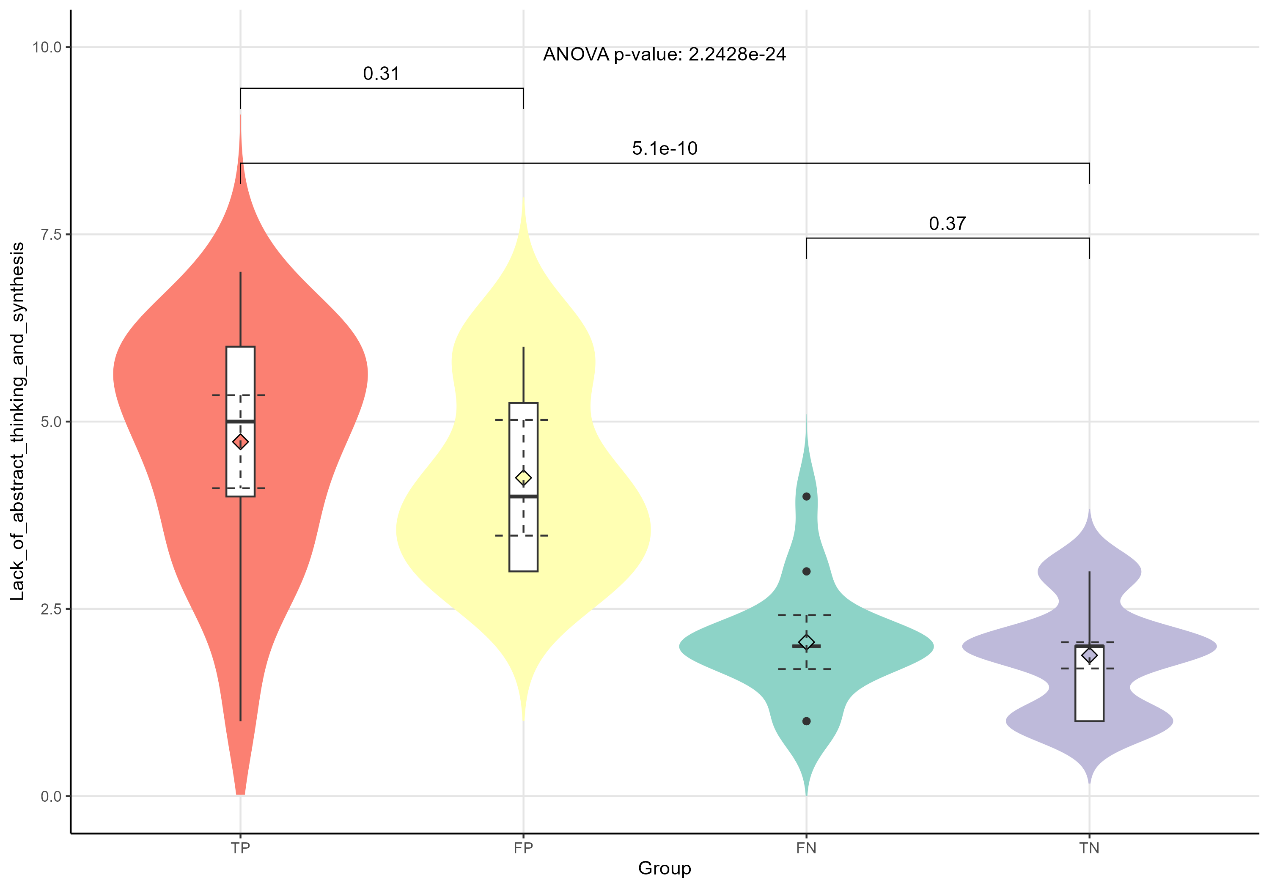


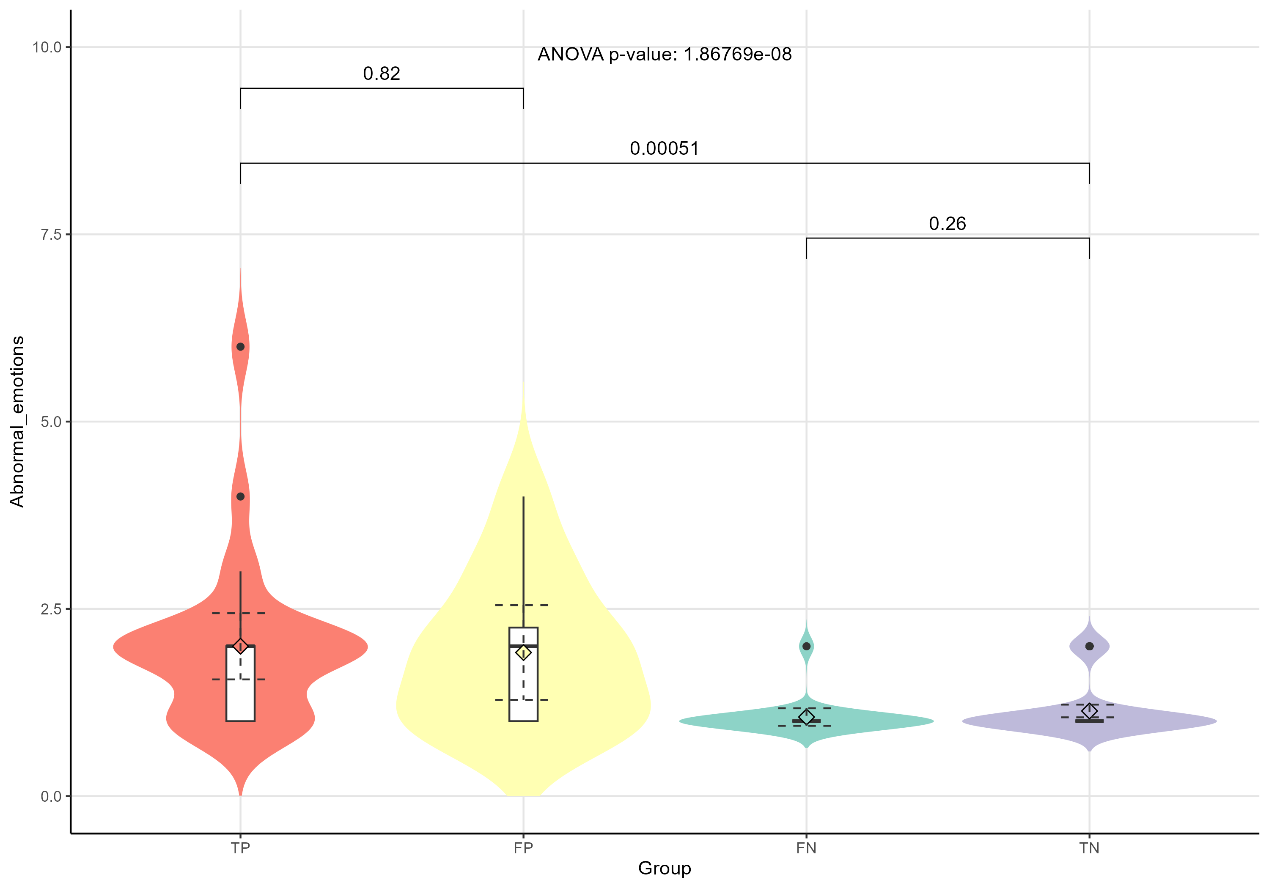


**Introduction to corpus and selection criteria**

**Introduction to corpus:**

DementiaBank English Protocol Delaware Corpus (Delaware Corpus):

The Delaware Corpus is a collection of linguistic data approved by the University of Delaware Institutional Review Board. It is constantly recruiting participants. As of the most recent update, the corpus contains data from 59 participants. Participants are primarily from the Community Elderly Registry at the Delaware Center for Cognitive Aging. Many of these participants have previously worked on memory research at the University of Delaware. The database has a variety of discourse protocols covering tasks such as picture description, storytelling, procedural discourse, and personal narrative.

DementiaBank English Pitt Corpus (Pitt Corpus):

The Pitt corpus had the highest usage rate in DementiaBank. Between March 1983 and March 1988, 319 individuals participated in the Alzheimer's Disease Research Programme. The programme provides transcripts and audio files from a longitudinal study involving 104 healthy adults, 208 adults diagnosed with dementia due to probable Alzheimer's disease and related dementia (ADRD), and 85 older adults with uncertain diagnoses.

**Selection criteria**: The selection criteria of Protocol Delaware Corpus and Pitt Corpus were designed to rule out other systemic or brain diseases that could lead to cognitive decline. Specifically:

| Corpus | DementiaBank English Protocol Delaware Corpus | DementiaBank English Pitt Corpus |
| --- | --- | --- |
| Age Requirement | All participants were 60 years or older. | Participants had to be older than 44 years. |
| Language Proficiency | Participants spoke and understood spoken English and they had adequate hearing and vision. | Participants had an education level surpassing the seventh grade. They were expected to read and write English fluently before the onset of dementia (for patients). |
| Neurological Health History | They had no self-reported history of major psychiatric disorders, untreated major depression, or medical illnesses that could induce cognitive decline, such as brain tumors, acquired brain injuries, or concussions. | Those with a history of major nervous system disorders were excluded. This includes cerebral trauma, stroke, meningitis, epilepsy, mental retardation, hypothyroidism, excessive use of drugs (including alcohol), malnutrition, and significant psychiatric disorders as per the Diagnostic and Statistical Manual of Mental Disorders, Third Edition. The only exception was dementia. |
| Medication Restrictions | Participants were stable on (or no) nootropic medications. | Participants were not on any neuroleptic or other medication that could influence central nervous system functions. The only exception allowed was antidepressants. |
| Neuropsychological Testing | The MCI classification was stringently based on the National Institute on Aging-Alzheimer's Association criteria. | They had to be cooperative during neuropsychological testing, with an initial Mini-Mental State Examination score of ≥10. |

These selection criteria were designed to ensure that participant group was as homogeneous as possible in terms of cognitive health, excluding potential confounders. Particularly, the exclusion of individuals with significant nervous system disorders and major psychiatric disorders ensures a decreased likelihood of including patients with learning disabilities and speech pathologies that could mimic symptoms of MCI or AD.
